# A silicon analogue of a fused bicyclic borirene derivative†

**DOI:** 10.1039/d4sc05867d

**Published:** 2025-01-27

**Authors:** Si Jia Isabel Phang, Zheng-Feng Zhang, Chi-Shiun Wu, Zhen Xuan Wong, Ming-Der Su, Cheuk-Wai So

**Affiliations:** a School of Chemistry, Chemical Engineering and Biotechnology, Nanyang Technological University Singapore 637371 Singapore CWSo@ntu.edu.sg; b Department of Applied Chemistry, National Chiayi University Chiayi 60004 Taiwan; c Department of Medicinal and Applied Chemistry, Kaohsiung Medical University Kaohsiung 80708 Taiwan

## Abstract

The replacement of all carbon atoms in aromatic rings with main-group elements to afford inorganic ring systems is highly desirable due to their distinct aromatic character. However, fused polycyclic main-group element rings are rare and the feasibility of aromaticity in such compounds has yet to be explored. To explore aromaticity in fused polycyclic main-group element rings, a stable di-silicon analogue of fused bicyclic borirene, namely bicyclo[1.1.0]-2,4-diborylenyldisil-1(3)-ene 5 was synthesized from an *N*-phosphinoamidinato chlorosilylene 3. Compound 5 consists of a bridgehead Si=Si double bond bonded with two bridging borons resulting in an unsaturated fused bicyclic skeleton. The bridgehead Si=Si σ- and π-electrons and bridging Si–B σ-electrons are stabilized by both σ- and π-aromatic delocalization on the Si_2_B_2_ fused bicyclic ring.

## Abstract 2

σ- and π-double aromatic delocalization is feasible in the smallest fused bicyclo[1.1.0]-2,4-diborylenyldisil-1(3)-ene composed of a bridgehead Si=Si double bond bonded with two bridging *N*-phosphinoamidinato borons.

## Graphical abstract

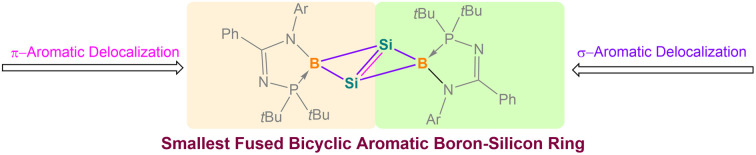

## Introduction

**Scheme 1:**
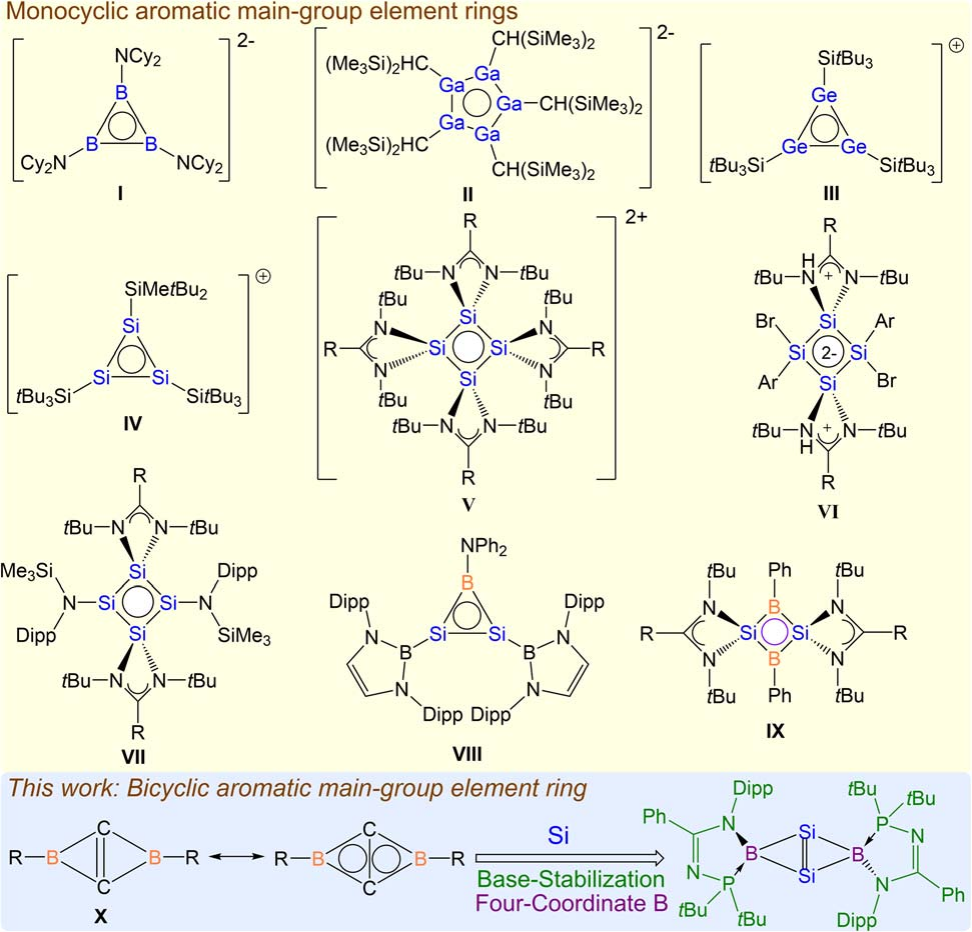
Aromatic main-group element rings.

Aromaticity is a key concept in understanding the electronic properties and reactivity of aromatic compounds in chemistry.^1^ Aside from the typical cyclic carbon based aromatic compounds such as benzene, aromaticity has been successfully extended to other main-group elements such as boron, silicon and phosphorus.^2^ Aromatic compounds with cyclic delocalized (4*n* + 2) electrons are important synthons in organic chemistry; therefore it is highly desirable to replace all carbon atoms in aromatic rings with main-group elements to afford inorganic ring systems that retain aromaticity.^2^ The diverse characteristics of main group elements compared to carbon give rise to distinct aromatic character which allows such rings to function as versatile building blocks for complex, yet highly functional molecules. However, the synthesis of such aromatic rings containing these main group elements is a formidable challenge due to the lack of methodology. To date, only a few stable examples have been reported and most examples consist of highly charged aromatic rings,^2–8^ such as triboracyclopropenyl dianion I,^3^ cyclopentagallene dianion II,^4^ cyclotrigermenium cation III,^5^ cyclotrisilenylium cation IV,^6^ and tetrasilacyclobutadiene dication V.^7^ Neutral aromatic rings are relatively rare;^9–16^ tetrasilacyclobutane-1,3-diyl VI,^10^ tetrasilacyclobutadiene VII,^11^ disilaborirene VIII,^12^ and cyclo-2,4-dibora-1,3-disilabutane-1,3-dilyl IX^13,14^ are such examples, and they were only synthesized recently (Scheme 1).

It is noteworthy that compounds I–IX are all mono-cyclic aromatic rings, and are representative main group element analogues of aromatic carbon molecules, such as cyclobutane-1,3-diyl and the cyclopropenyl cation. Based on fused polycyclic aromatic hydrocarbons such as naphthalene, it is plausible to extend the concept of aromaticity to fused polycyclic inorganic rings, which have yet to be discovered.

Among fused polycyclic aromatic rings, bicyclic borirene X is the smallest neutral fused aromatic ring.^17^ It features bicyclic 2π-aromatic delocalization, resulting from the overlapping of the bridgehead C=C π orbital with two empty p orbitals on the bridging boron center. Compared to typical alkenes such as ethylene and propylene, high strain energy owing to the exceptionally strained bridgehead C=C double bond in the bicyclic scaffold, arising from an inversion of the conformation about the C=C double bond, is expected. The isolation of such a ring remains unknown experimentally. Thus, in an effort to isolate a more stable analogue of bicyclic borirene X, we replaced the carbon atoms in the ring with silicon to offer more chemical adaptability and reduced ring strain due to the larger atomic size of silicon. We further used a bidentate ligand bonded with the boron atoms to provide enhanced kinetic stabilization. Herein, we report a phosphinoamidinate-stabilized bicyclo[1.1.0]-2,4-diborylenyldisil-1(3)-ene, where the bridgehead Si=Si σ- and π-electrons and bridging Si–B σ-electrons are aromatically delocalized on the Si_2_B_2_ fused bicyclic ring, accounting for the stability of the fused bicyclic borirene ring structure.

## Results and discussion

### Synthesis and characterization

**Scheme 2:**
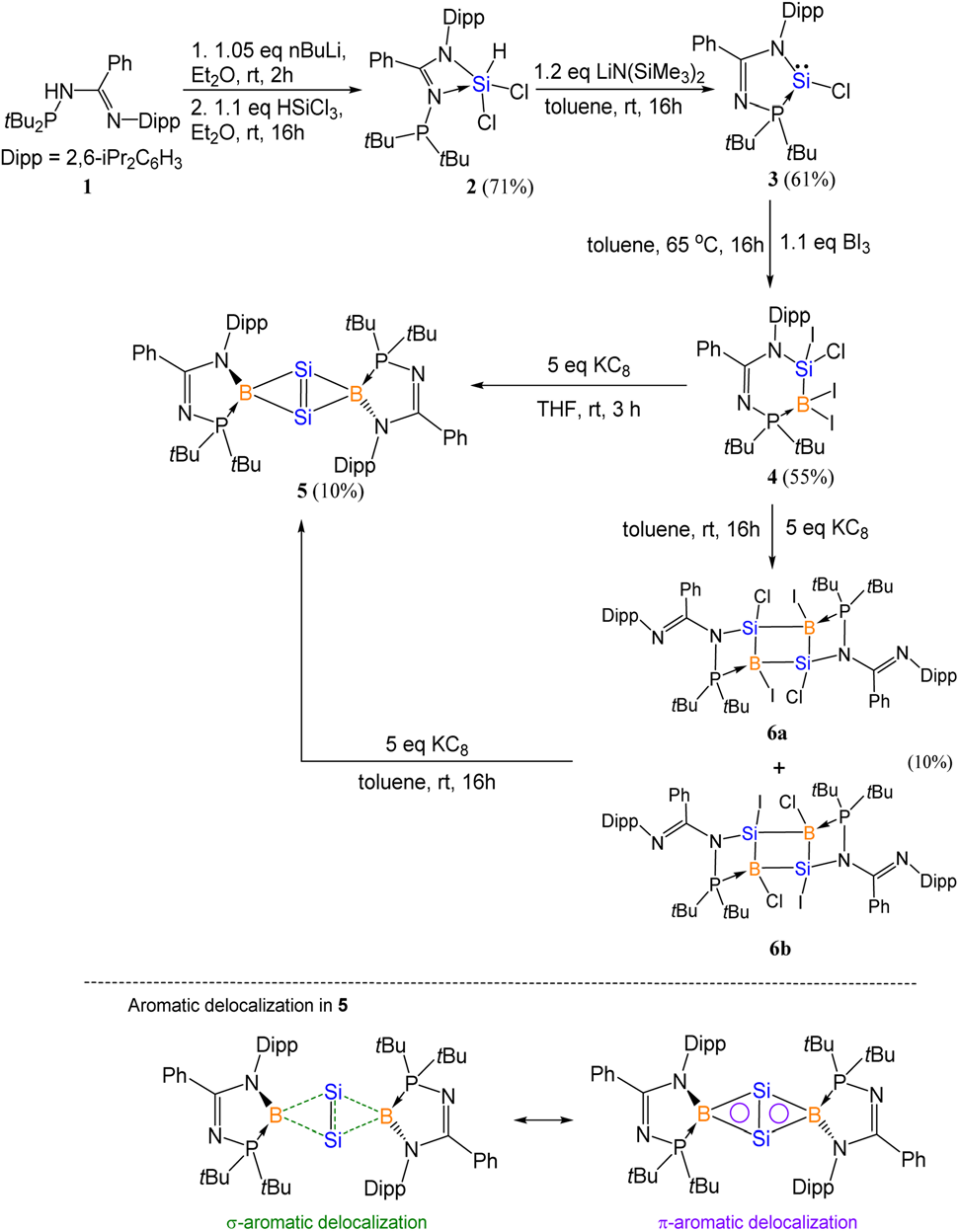
Synthesis of compound 5 (yields reported are all isolated yields).

**Fig. 1:**
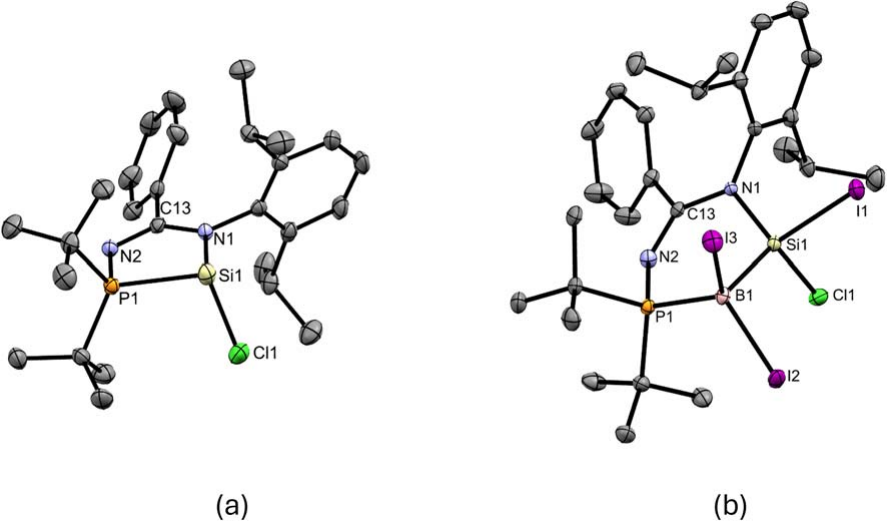
X-ray crystal structures of 3 and 4 with thermal ellipsoids at 50% probability. All H atoms are omitted for clarity. (a) Selected bond lengths (Å) and angles (deg.) of 3: Si1–N1 1.862(3), Si1–P1 2.3685(13), Si1–Cl1 2.1770(14), P1–Si1–N1 81.03(9), P1–Si1–Cl1 99.06(5), N1–Si1–Cl1 99.89(10). (b) Selected bond lengths (Å) and angles (deg.) of 4: Si1–B1 2.021(5), Si1–N1 1.787(3), Si1–I1 2.4356(11), Si1–Cl1 2.0845(15), B1–I2 2.243(5), B1–I3 2.260(5), B1–P1 2.009(5), N1–Si1–B1 112.94(17), Si1–B1–P1 101.1(2), B1–P1–N2 110.17(19).

The *N*-phosphinoamidinato dichlorosilane 2 (Fig. S27†) was prepared and reacted with LiN(SiMe_3_)_2_·Et_2_O in toluene at room temperature for 16 h to form *N*-phosphinoamidinato chlorosilylene 3 (Scheme 2), which was isolated as a yellow crystalline solid (yield: 61%). The *N*-phosphinoamidinate ligand is bonded with the silylene center in an *N*,*P*-chelate fashion. Its ^31^P{^1^H} NMR spectrum shows a singlet at 67.8 ppm with a pair of satellite signals (*J*_Si–P_ = 186.5 Hz), which is upfield shifted compared with that of (phosphine)(amido)bromosilylene (74.7 ppm).^18^ The ^29^Si{^1^H} NMR spectrum shows a doublet at 8.0 ppm (*J*_Si–P_ = 186.6 Hz), which is intermediate between that of (phosphine)(amido)bromosilylene (−18.4 ppm)^18^ and amidinato chlorosilylene (14.6 ppm).^19^ The X-ray crystal structure shows the silicon center adopting a trigonal pyramidal geometry (Fig. 1a, sum of the bond angles: 280.0°), indicating the presence of a Si lone pair of electrons.

Compound 3 underwent oxidative addition with BI_3_ in toluene at 65 °C for 16 h to afford the *N*-phosphinoamidinate-bridged borylsilane 4 (Scheme 2), which was isolated as a colorless crystalline solid (yield: 55%). The ^29^Si{^1^H} NMR signal of 4 (−8.8 ppm) is broad due to quadrupolar coupling with the B nucleus. The ^31^P{^1^H} and ^11^B{^1^H} NMR signals are found at 26.6 ppm and −45.6 ppm, respectively. X-ray crystallography shows a six-membered ring, where the ligand is an *N*,*P*-chelate, bridged between the Si–B bond (Fig. 1b). The Si1–B1 bond (2.021(5) Å) is typical of a single bond. A similar B–Cl bond oxidative addition was observed in the reactivity of a disilicon(i) compound.^20^

**Scheme 3:**
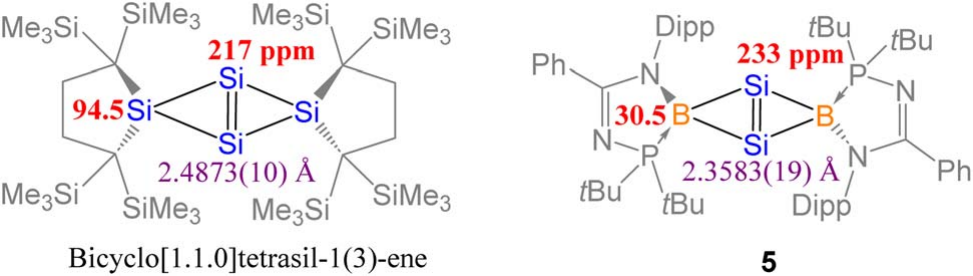
Comparison of NMR signals (in red) and Si=Si bond lengths (in purple) between bicyclo[1.1.0]tetrasil-1(3)-ene and compound 5.

**Fig. 2:**
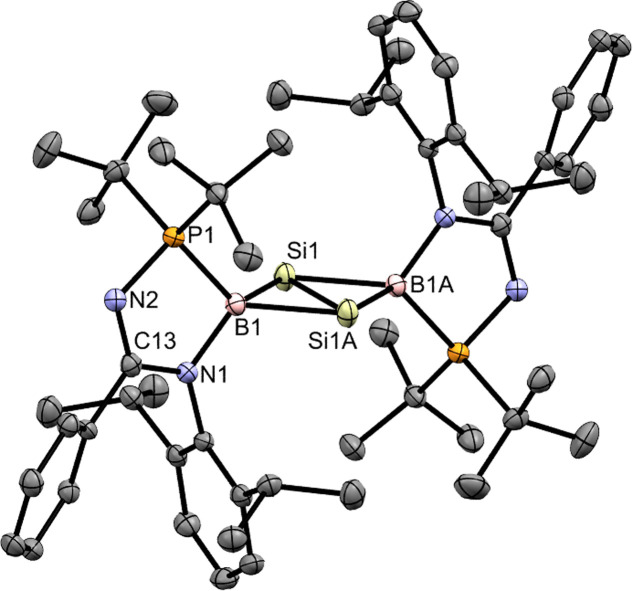
X-ray crystal structure of 5 with thermal ellipsoids at 50% probability. All H atoms are omitted for clarity. Selected bond lengths (Å) and angles (deg.): Si1–Si1A 2.3583(19), B1–Si1 2.034(4), B1–Si1A 2.048(3), B1–P1 1.970(3), B1–N1 1.550(4), B1–Si1–Si1A 54.98(10), B1–Si1–B1A 109.41(12), P1–B1–N1 95.34(18).

Compound 4 was reacted with excess KC_8_ in THF at room temperature for 3 h to afford *N*-phosphinoamidinato bicyclo[1.1.0]-2,4-diborylenyldisil-1(3)-ene 5, which was isolated as a reddish-orange crystalline solid (yield: 10%). Compound 5 is considered a bridging boron analogue of the bicyclo[1.1.0]tetrasil-1(3)-ene reported by Iwamoto *et al.*,^21–25^ but the presence of the bridging *N*-phosphinoamidinato boron moieties in compound 5 induces some degree of difference in electronic properties compared with the bicyclo[1.1.0]tetrasil-1(3)-ene, as indicated by NMR spectroscopy and X-ray crystallography (Scheme 3). The ^31^P{^1^H} NMR signal (40.9 ppm) is broad and the ^11^B{^1^H} NMR signal (30.5 ppm) is in the low-field region, even though the boron centers in compound 5 are four-coordinate. It is upfield shifted in comparison with that of the endocyclic boron center in VIII (50.1 ppm).^12^ The ^29^Si{^1^H} NMR resonance (233.0 ppm) is intermediate between cyclotrisilenylium cations (284.6–288.1 ppm)^6^ and bicyclo[1.1.0]tetrasil-1(3)-ene (217 ppm).^21^ The broad ^29^Si{^1^H} NMR signal of compound 5 is also downfield shifted in comparison with those of disilenes (50–155 ppm)^26^ and VIII (112.2 ppm). The molecular structure of 5 obtained by X-ray crystallography shows the planar fused bicyclo-Si_2_B_2_ ring being orthogonal to two *N*-phosphinoamidinate ligands (Fig. 2). It is expected that the exceptionally strained bridgehead Si=Si double bond in the bicyclic scaffold distorts the structural parameters. The Si–B (2.034(4), 2.048(3) Å) and Si1–Si1A (2.3583(19) Å) bond lengths are longer than those in compound VIII (Si–B: 1.911(7)–1.952(3); Si–Si: 2.133(2)– 2.1469(11) Å).^12^ The Si1–Si1A bond is intermediate between those in disilenes (2.14–2.29 Å)^26^ and bicyclo[1.1.0]tetrasil-1(3)-ene (2.4873(10) Å).^21^ The Si–Si bond length in compound 5 is comparable with the Si–Si single bond length (*ca.* 2.3 Å). The UV-vis spectrum of compound 5 in THF (dark orange solution) shows an absorption band at *λ*_max_ = 412 nm corresponding to the 
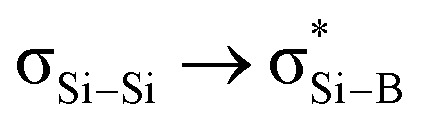
 (HOMO−2 → LUMO) and 
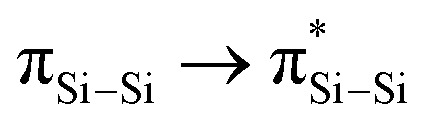
 (HOMO → LUMO+2) with equal contribution, and an intense absorption band at 474 nm corresponding to the 
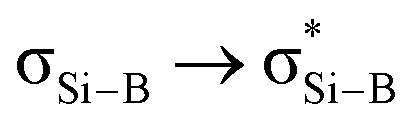
 (HOMO−1 → LUMO).

The downfield ^11^B{^1^H} and ^29^Si{^1^H} NMR signals together with long Si=Si double bond length indicate that the bridgehead Si=Si σ and π electrons in compound 5 could delocalize in the fused bicyclic scaffold. However, the multinuclear NMR signals and the distorted structural parameters of the Si_2_B_2_ ring cannot definitively confirm the presence of aromaticity. ^1^H NMR spectroscopy is a widely used experimental tool to assess whether a molecule is aromatic, although determining the degree of aromaticity remains a challenge.^27^ In the case of compound 5, there are no ^1^H NMR signals for the Si_2_B_2_ ring. Therefore, the degree of electron delocalization as σ- and π-aromaticity can only be evaluated through DFT calculations, which is discussed later in the manuscript.

**Fig. 3:**
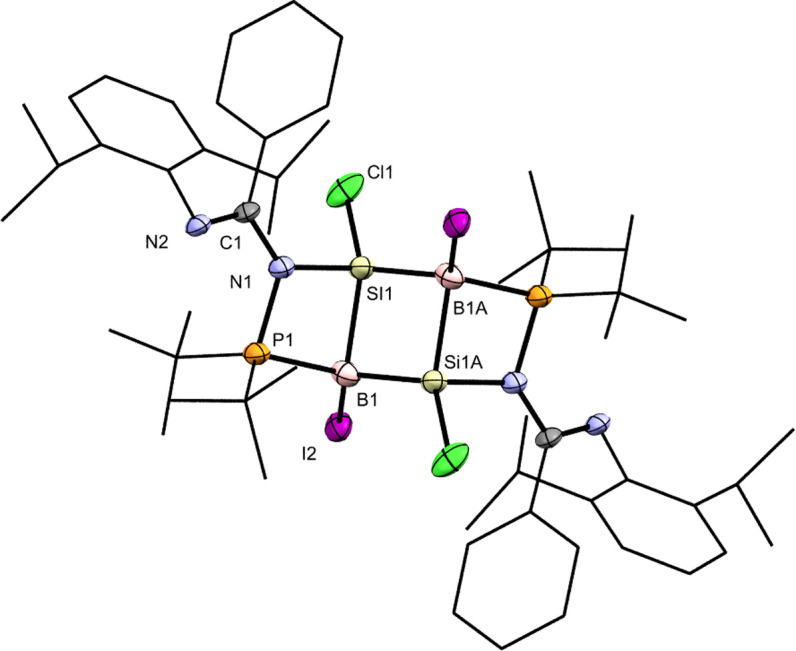
X-ray crystal structure of 6a with thermal ellipsoids at 50% probability. All H atoms are omitted for clarity. Selected bond lengths (Å) and angles (deg.): Si1–B1 2.033(10), Si1–B1A 2.025(11), Si1–N1 1.804(7), B1–P1 1.979(10), P1–N1 1.716(7), C1–N1 1.416(10), C1–N2 1.281(10), B1A-Si1-B1 95.6(4), Si1–B1–Si1A 84.4(4), N1–Si1–B1 86.2(4), Si1–B1–P1 83.8(4), B1–P1–N1 90.3(4), P1–N1–Si1 99.2(3).

The reaction of 4 with KC_8_ was performed in a less polar solvent, namely toluene, at room temperature (Scheme 2). The reaction was traced by ^31^P{^1^H} and ^11^B{^1^H} NMR spectroscopy after stirring for 16 h, where a mixture of 1,3-dibora-2,4-disilacyclobutanes 6a and 6b was observed (^31^P{^1^H} NMR: 102.4 and 108.4 ppm; ^11^B{^1^H} NMR: −11.3 and −34.8 ppm). They were isolated as a co-crystalline colorless solid (Fig. 3, yield: 10%) from the reaction mixture. The formation of 6a and 6b indicates that halogen scrambling occurred during the reduction. X-ray crystallography of 6a shows that the *N*-phosphinoamidinate ligands are in a *P*,*N*-chelate fashion, bridging across the B–Si bonds (Fig. 3). The B–Si bond lengths (2.033(10) and 2.025(11) Å) are comparable to those of 5. The Si1–N1 (1.804(7) Å) and P1–B1 bonds (1.979(10) Å) are typical single bonds. The C–N bond lengths (C1–N1: 1.416(10) and C1–N2: 1.281(10) Å) are unequal, showing that the exocyclic N2 atom is an imine moiety. When the reaction of 4 with KC_8_ in toluene was performed for 3 days, compound 5 was observed, indicating that compounds 6a and 6b are intermediates for the formation of compound 5. To support this, a mixture of compounds 6a and 6b were reacted with excess KC_8_ in toluene for 16 h, leading to the formation of compound 5.

From compounds 6a and 6b, it is plausible that the reduction of compound 4 with KC_8_ proceeds through the formation of compounds 6a and 6b first, followed by further reduction to form a diboradisilacyclobutadiene intermediate, which is anti-aromatic (Scheme S1†). Subsequently, it undergoes rearrangement by coordinating the *N*-phosphinoamidinate ligands with the boron centers to form the bridgehead Si=Si double bond and bridging boron in compound 5.

### The electronic properties of the bridgehead Si=Si double bond

**Fig. 4:**
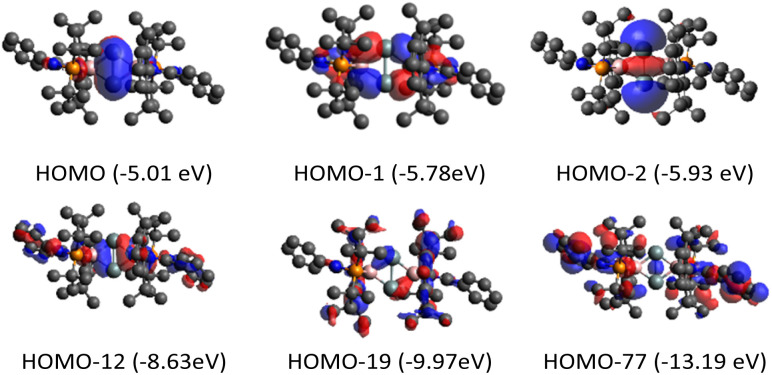
Molecular orbitals of 5 at the M06-2X/Def2-TZVP level of theory.

DFT calculations (M06-2X/def2-TZVP) were performed to elucidate the electronic structure of 5 (Fig. 4). Calculations revealed a singlet ground state of 5 with a singlet-triplet energy gap of 17.4 kcal mol^−1^. First, the molecular orbital analysis shows that the highest occupied molecular orbital (HOMO) is a π orbital delocalized over the entire fused bicyclic Si_2_B_2_ ring. It can be considered as the overlapping of p_π_ orbitals at the Si atoms with the B–N and B–P σ* orbitals, having pseudo π symmetry at the boron atoms. A total occupancy value of 0.59 e in B–N and B–P σ* orbitals from the NBO analysis reaffirms their participation in delocalization. The NBO analysis does not show a Si–Si π orbital, but instead shows two Si p_π_ orbitals with an occupancy value of 0.76 e. These NBO data suggest that the Si–Si π bond (total electron occupancy = 0.76 + 0.76 = 1.52 e) is weak due to electron delocalization (0.59 e) with the B–N and B–P σ* orbitals. In comparison, Cui *et al.* reported tetrasilacyclobutane-1,3-diyl VI,^10^ where 2π electrons are aromatically delocalized through the σ* orbitals with pseudo π symmetry on the four-coordinate silicon centers. The HOMO−2 represents the σ-interaction between two bridgehead silicon atoms, which significantly extends over the fused bicyclic Si_2_B_2_ ring. The HOMO−1, HOMO−12, HOMO−19 and HOMO−77 show the presence of four B–Si σ orbitals. Second, the Wiberg bond index (WBI) between the bridgehead silicon atoms is 1.01, indicating weak Si=Si double bond character.

**Fig. 5:**
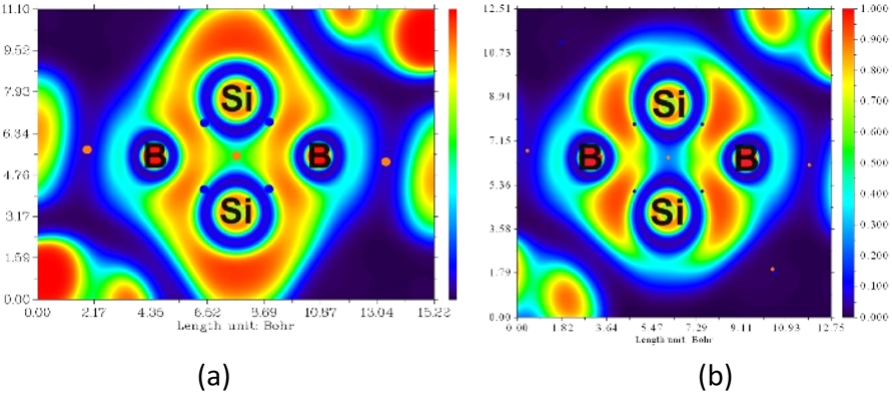
(a) ELF map of the fused bicyclic Si_2_B_2_ ring in 5; red and blue represent the strong and weak degree of electron localization of the core electrons, respectively. (b) Removing electrons from the σ-bonding HOMO−2 results in an almost non-existent σ bond between the two bridgehead Si atoms (ELF ≈ 0.01).

To understand the nature of the Si–Si σ-orbital, the electron localization function (ELF) was calculated (Fig. 5a). The ELF plot of the fused bicyclic Si_2_B_2_ ring reveals four Si–B bond critical points (blue dots) and one Si_2_B_2_ ring critical point (orange dot). Notably, a weak σ-interaction is observed between the two bridgehead Si atom as reflected by the small ELF values (in green). It is because some of the Si–Si σ-electrons are delocalized with the B–Si σ-electrons (annular red region). Upon the removal of σ electrons from HOMO−2 (Fig. 5b), the distribution of energy density is altered, revealing a nearly non-existent σ bond between the two bridgehead Si atoms (in blue). This confirms the presence of a weak Si–Si σ bond which cannot be completely disregarded.

**Fig. 6:**
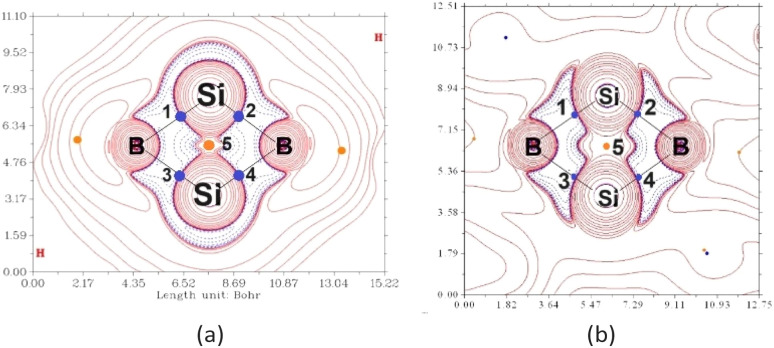
(a) Laplacian distribution of electron energy of the fused bicyclic B_2_Si_2_ ring. Positive and negative areas are shown by crimson and blue lines, representing electron depletion and accumulation, respectively. (b) By removing electrons from the σ-bonding HOMO−2, the σ-electron density concentrations within the fused bicyclic B_2_Si_2_ σ skeleton are altered.

A quantum theory of atoms in molecules (QTAIM) analysis was conducted to quantify the Si–Si σ-interaction. QTAIM revealed the formation of 4 Si–B σ bonds, as seen from the bond critical point (BCP: blue dot 1–4, Fig. 6a) along each Si–B bond path. The mean Laplacian distribution ∇^2^*ρ*(*r*_c_) and energy density *H*(*r*) of the Si–B bonds at the BCP are 0.009 e a^−5^ and –0.060 hartree a^−3^, respectively. It should be noted that the π-electron density at the ring critical point (RCP) becomes zero, which is attributed to the RCP being located precisely on the nodal plane of the π orbital. As such, the values of ∇^2^*ρ*(*r*_c_) (0.008 e a^−5^) and *H*(*r*) (−0.032 hartree a^−3^) observed at the Si_2_B_2_ RCP (orange dot 5) indicate the presence of a distinct and weak σ bond between the two Si atoms. Upon the removal of σ electrons from HOMO−2 (Fig. 6b), the *H*(*r*) of the RCP reduced from −0.032 hartree a^−3^ to −0.017 hartree a^−3^, suggesting that HOMO−2 contributes to the weak σ bond between the two bridgehead Si atoms. These results are in line with ELF calculations. The feasibility of weak σ interaction in the strained and saturated fused bicyclic ring system has also been illustrated by Foroutan-Nejad's theoretical calculations.^28^

### Electronic delocalization in bicyclo[1.1.0]-2,4-diborylenyldisil-1(3)-ene 5

Recently, Driess *et al.* showed that a highly strained all-germanium spiro molecule containing one Ge=Ge double bond in each spiro ring, namely spiro[2.2]pentagerma-1,3-diene, exhibits both σ and 2π electronic delocalization in order to consolidate the pentagerma-spiro skeleton and to reduce the magnitude of the ring strain, respectively.^29^ In addition, Cui *et al.* pointed out that Iwamoto's bicyclo[1.1.0]tetrasil-1(3)-ene^21^ possesses both σ and π delocalization through DFT calculations.^10^ We reported a highly strained all-silicon spiro molecule containing two bent trisilicon-allene skeletons in each spiro ring, namely spiro[3.3]heptasila-2,6-diylidone possessing both σ and π electronic delocalization to enhance the stability of the entire molecule.^30^ In this context, it is anticipated that the stability of the fused bicyclic borirene skeleton in compound 5 could arise from some degree of σ and π electronic delocalization, as indicated by the HOMO and ELF calculations.

**Fig. 7:**
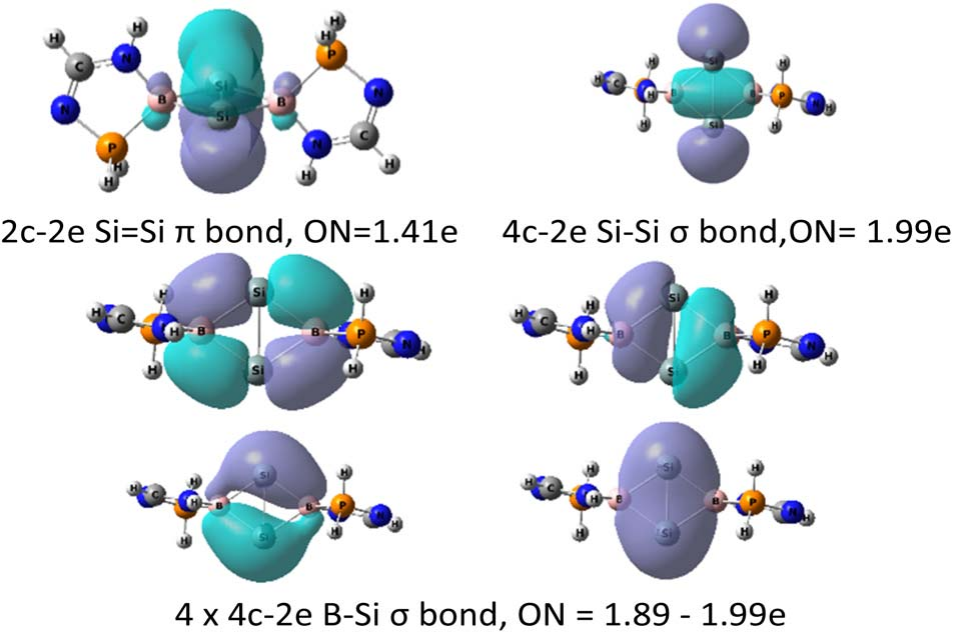
Adaptive natural population density (AdNDP) analysis of a simplified truncated model 5-H, where substituents (Dipp, Ph, and *t*Bu) in compound 5 are substituted with hydrogen atoms for clarity.

To understand the overall σ and π electronic delocalization in the entire Si_2_B_2_ ring, adaptive natural density partitioning (AdNDP) analysis was performed to elucidate the electronic structure of 5 in terms of the classical Lewis elements (lone-pairs and two-center-two-electron bonds) and delocalized *n*-center two-electron (*n*c-2e) bonds (Fig. 7) in order to identify possible delocalized electron-pair bonding.^31^ A simplified truncated model 5-H was used since all the bonding features of interest are located at the Si_2_B_2_ core in 5, which is preserved and clearly presented in the model. First, a 2-center-2-electron π-bond with occupation number (ON) = 1.41 e was found between two Si atoms (Fig. 7, top left). Such low occupancy indicates that the Si–Si π-bond is delocalized over the Si_2_B_2_ bicyclic ring, supporting the presence of 2π-aromaticity. Second, a 4-center-2-electron σ bond with ON = 1.99 e is found between two silicon atoms (Fig. 7, top right). In addition, four 4-center-2-electron σ bonds with ON = 1.88–1.99 e are observed at the Si_2_B_2_ ring (Fig. 7, bottom). These five 4-center-2-electron σ bonds indicate the presence of σ-aromatization,^32^ which is also illustrated by the contour plot (annular red region) in ELF calculations (Fig. 5a).

**Fig. 8:**
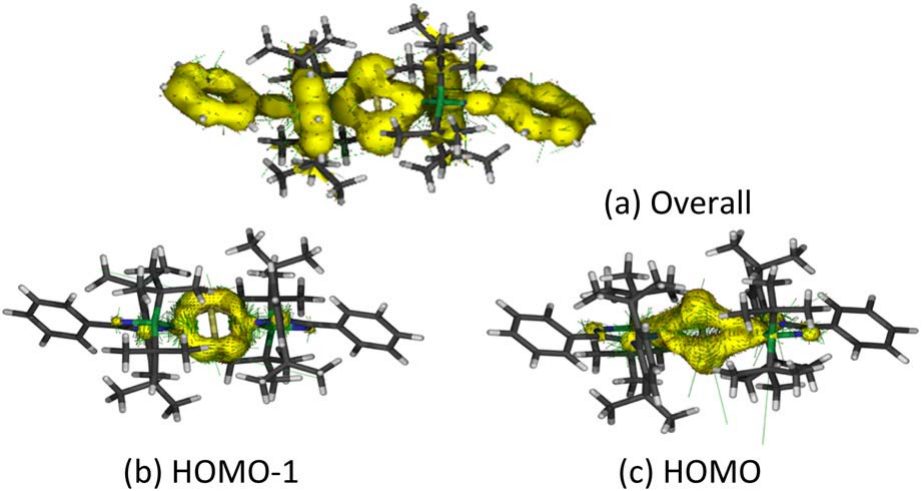
Anisotropy of the current induced density (ACID) of 5. (a) Overall, (b) HOMO-1, (c) HOMO.

Anisotropy of the induced current density (ACID) was calculated to elucidate the σ- and π-aromaticity. The current density vectors plotted onto the ACID isosurface indicate a strong diatropic ring current in the π system above and underneath the ring, showing π-aromaticity in the Si_2_B_2_ ring (Fig. 8a). It is supported by the strong diatropic ring current in the HOMO (Fig. 8c), where the Si–Si π bond exhibits 2π-aromatic delocalization. Besides π delocalization, the current density vectors plotted onto the ACID isosurface indicate a diatropic ring current in the molecular plane mainly localized inside the four-membered ring (Fig. 8a). This is supported by the diatropic ring current in HOMO-1 (Fig. 8b), which illustrates the presence of σ-tangential aromatic delocalization^33^ among the Si–B bonds. It is noteworthy that the diatropic ring current is restricted to the periphery. A similar observation can be found in naphthalene.^34^ In addition, the positive natural population analysis (NPA) charge on the bridgehead Si atoms (0.36*e*) and the negative NPA charge on the B atoms (−0.55*e*) suggest that the electron density in the ring flows from the silicon to boron atoms. The highly negative Nucleus Independent Chemical Shift (NICS) values at the center of each three-membered ring [NICS(0): −38.7, NICS(1): −13.8, NICS(1)_*zz*_: −22.4 ppm] and at the center of the whole Si_2_B_2_ four-membered ring [NICS(0): −38.5, NICS(1): −22.1, NICS(1)_*zz*_: −34.0 ppm] indicate considerable inorganic aromaticity in the fused bicyclic 2π-Si_2_B_2_ ring.

To further quantify the aromaticity of the B_2_Si_2_ four-membered ring in compound 5, the electron density of delocalized bonds (EDDB) was employed. The EDDB value for compound 5 is 1.901 e. The EDDB value comprises the EDDB_σ_ component (1.193 e) and the EDDB_π_ component (0.708 e) within the B_2_Si_2_ four-membered ring of compound 5. These results illustrate that compound 5 possesses both σ- and π-aromaticity in the Si_2_B_2_ ring of compound 5. The EDDB_π_ component (0.708 e) is comparable with that of VI and related 2π-aromatic Si_4_ ring analogues (0.586–0.979 e, CAM-B3LYP/def2-TZVP).^10^ Other ring systems containing both σ and π aromatic delocalization have been reported by Saito and Berndt.^35^

**Fig. 9:**
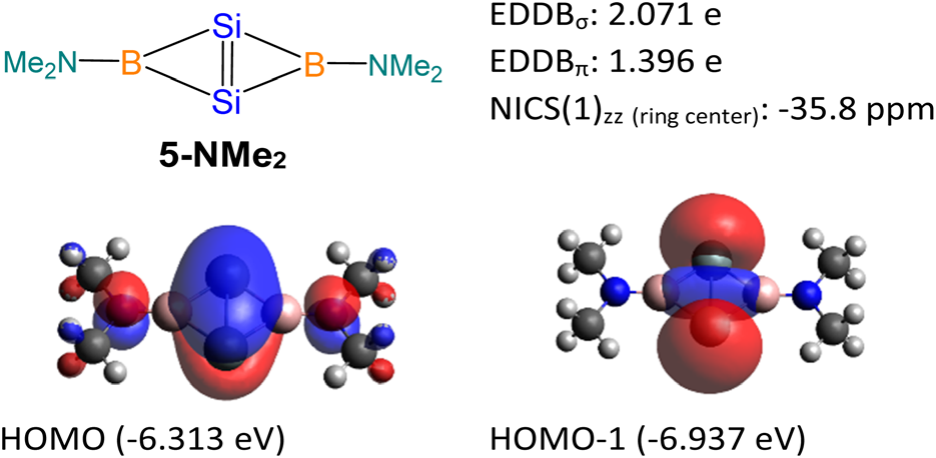
Theoretical data of 5-NMe_2_.

5-NMe_2_ with two -NMe_2_ substituted tricoordinate boron centers was used as a model molecule (Fig. 9), and its NICS and EDDB calculations were performed for comparison with compound 5. Similar to compound 5, the 5-NMe_2_ model possesses both σ- and π-aromatic delocalization. The HOMO of the 5-NMe_2_ model is a π orbital delocalized over the entire fused bicyclic Si_2_B_2_ ring. It can be considered as the overlapping of p_π_ orbitals on the Si atoms with the B–N π* orbitals. The HOMO-1 of the 5-NMe_2_ model represents the σ-interaction between two bridgehead silicon atoms, which significantly extends over the fused bicyclic Si_2_B_2_ ring. The NICS value at the center of the Si_2_B_2_ ring [NICS(0): −37.5, NICS(1): −23.2, NICS(1)_*zz*_: −35.8 ppm], the EDDB_σ_ value (2.071 e) and the EDDB_π_ value (1.396 e) illustrate the σ- and π-aromaticity in the Si_2_B_2_ ring of the 5-NMe_2_ model. The higher EDDB_π_ value of 5-NMe_2_ is attributed to the empty p orbital on the boron center which allows for more effective overlapping with the Si=Si π orbital. The lower EDDB_π_ value in compound 5 is due to the less effective overlapping of p_π_ orbitals on the Si atoms with the B–N and B–P σ* orbitals, having pseudo π symmetry at the four-coordinate boron atoms.

### Reactivity of bicyclo[1.1.0]-2,4-diborylenyldisil-1(3)-ene 5

**Scheme 4:**
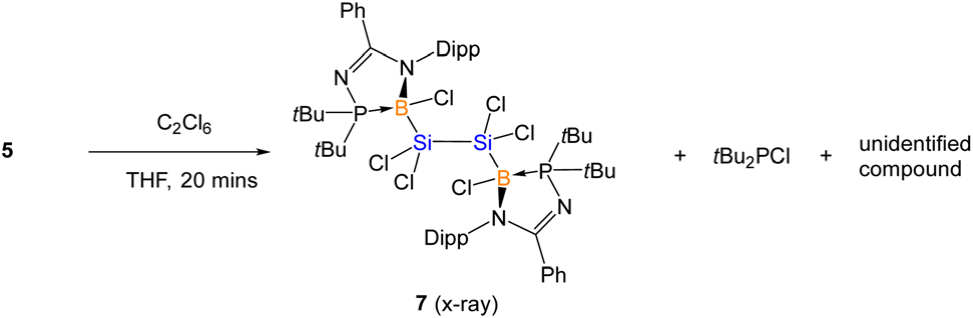
Synthesis of compound 7.

**Fig. 10:**
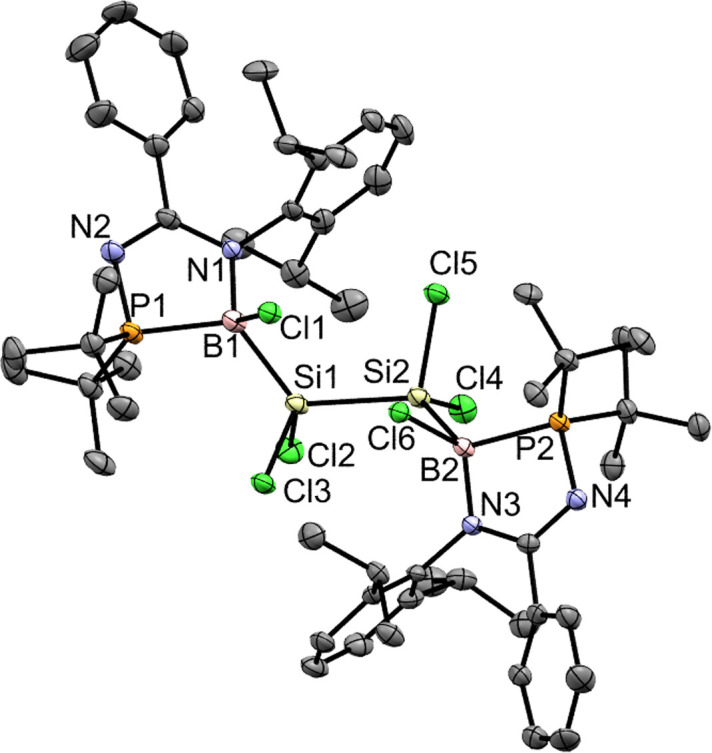
X-ray crystal structure of 7 with thermal ellipsoids at 50% probability. All H atoms are omitted for clarity. Selected bond lengths (Å) and angles (deg.): Si1–Si2 2.4114(16), Si1–B1 2.080(6), Cl2–Si1 2.1008(16), Cl3–Si1 2.0837(15), B1–N1 1.550(6), B1–Cl1 1.900(5), Cl4–Si2 2.1043(16), Cl5–Si2 2.0738(15), B2–N3 1.546(6), B2–Cl6 1.898(5), B2–P2 2.025(5), B2–Si2 2.085(5), N1–B1–Cl1 109.9(3), Cl1–B1–P1 109.0(3), N1–B1–P1 95.5(3), N1–B1–Si1 128.6(3), Cl1–B1–Si1 92.6(2), P1–B1–Si1 120.6(2), N3–B2–Cl6 109.4(3), Cl3–Si1–Cl2 100.88(6).

The lack of reactivity of compound 5 with small molecules (examples: H_2_ and CO), unsaturated molecules (example: *p*-tolyl isocyanate) and transition metal complexes (examples: [Ir(cod)Cl]_2_, PdCl_2_, and Pt(P*t*Bu_3_)_2_), can be attributed to the electronic delocalization of 5 which enhances the stability of the Si=Si and Si–B bonds. Reactivity with the chlorinating agent (C_2_Cl_6_) led to the oxidation of compound 5 resulting in a mixture of compounds including compound 7 and di-*tert*-butylchlorophosphine (Scheme 4 and Fig. 10). Reaction with protic reagents (examples: MeOH, NH_3_BH_3_ and H_2_O), and boron substrates (BI_3_ and B(OH)_3_) leads to the hydrolysis of compound 5 to form the free *N*-phosphinoamidine ligand (tracked by *in situ*^31^P{^1^H} NMR), indicating the decomposition of 5.

## Conclusions

In conclusion, the first base-stabilized di-silicon analogue of fused bicyclic borirene, namely bicyclo[1.1.0]-2,4-diborylenyldisil-1(3)-ene 5 was synthesized. It consists of a bridgehead Si=Si double bond bonded with two bridging *N*-phosphinoamidinato boron moieties. The bridgehead Si=Si σ- and π-electrons and bridging Si–B σ-electrons are both σ- and π–aromatically delocalized at the fused Si_2_B_2_ bicyclic ring. Other main-group element analogues of fused bicyclic borirene are currently under investigation.^36^

## Data availability

The data supporting this article have been included as part of the ESI.† Deposition numbers 2266104 (for 2), 2266105 (for 3), 2266106 (for 4), 2266107 (for 5), 2266108 (for 6) and 2380825 (for 7) contain the supplementary crystallographic data for this paper. These data are provided free of charge by the joint Cambridge Crystallographic Data Centre and Fach Informationszentrum Karlsruhe Access Structures service.

## Author contributions

S. J. I. Phang performed experiments. Z.-F. Zhang, C.-S. Wu, Z. X. Wong, and M.-D. Su conducted theoretical calculations. C.-W. So prepared the manuscript.

## Conflicts of interest

There are no conflicts to declare.

## Supplementary Material

SC-016-D4SC05867D-s001

SC-016-D4SC05867D-s002
