# Supplementary material for: A silicon analogue of a fused bicyclic borirene derivative
Source: Chem Sci. 2025 Jan 27;16(10):4512–8. doi: 10.1039/d4sc05867d (PMC11803951; doi:10.1039/d4sc05867d)
Supplement: SC-016-D4SC05867D-s001 [file SC-016-D4SC05867D-s001.pdf]

Supplementary Information (SI) for Chemical Science.
This journal is © The Royal Society of Chemistry 2025

Supporting Information

A Silicon Analogue of Fused Bicyclic Borirene Derivative

Si Jia Isabel Phang,[a] Zheng-Feng Zhang,[b] Chi-Shiun Wu,[b] Zhen Xuan Wong,[a] Ming-Der Su,[b,c*] and Cheuk-Wai So[a*]

[a]School of Chemistry, Chemical Engineering and Biotechnology, Nanyang Technological University, Singapore 637371. [b]Department of Applied Chemistry, National Chiayi University, Chiayi 60004, Taiwan. [c]Department of Medicinal and Applied Chemistry, Kaohsiung Medical University, Kaohsiung 80708, Taiwan

Table of Contents

S1. Experimental Section

S2. Selected NMR spectra

S3. UV-vis spectra

S4. X-Ray Data Collection and Structural Refinement

S5. Theoretical Studies

S1. Experimental Procedures

General procedure. All manipulations were carried out under an argon atmosphere with Schlenk techniques and glovebox. Hexane, toluene and diethyl ether were purified through a MBRAUN solvent purification system. Tetrahydrofuran and heptane were purified by distillation over potassium/benzophenone. Benzene-d_6, tetrahydrofuran-d_8 and toluene-d_8 were distilled over potassium metal. Chemicals were purchased from Sigma-Aldrich and directly used without purification. Compound **1** was synthesized according to reported procedures.[S1,S2] ^{1}H, ^{11}B{^{1}H}, ^{31}P{^{1}H}, ^{13}C{^{1}H} and ^{29}Si{^{1}H} NMR spectra were measured on a Bruker Avance III 400 with a Dual Resonance Probe (BBFO) or JEOL (ECA 400) spectrometer. Deuterated solvents were used for the recording of NMR spectra, and chemical shifts are given in δ (ppm) and coupling constants *J* in Hz. NMR multiplicities are abbreviated, where s = singlet, d = doublet, m = multiplet, sep = septet and br = broad signal. The solid-state ^{31}P, ^{29}Si and ^{11}B NMR experiments were conducted at 11.7 T on a 500 MHz JEOL NMR spectrometer (JNM-ECZL500G) and equipped with a 3.2 mm double-resonance HXMAS probe. The ^{31}P{^{1}H} and ^{11}B{^{1}H} solid state NMR spectroscopy were ran using single pulse decoupled experiment at 8 kHz with reference to $NH_4H_2PO_4$ (2.14 ppm) and $NaBH_4$ (-3.61 ppm), respectively. The ^{29}Si{^{1}H} solid state NMR spectroscopy was ran using a single pulse experiment at 12 kHz with reference to silicone rubber (-21.50 ppm). HRMS spectra were obtained at the Mass Spectrometry Laboratory in the School of Chemistry, Chemical Engineering and Biotechnology, Nanyang Technological University.

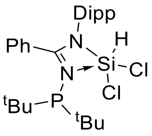

Synthesis of **2**. N-phosphinoamidine **1** (1.27 g, 3 mmol) was dissolved in diethyl ether (60 ml) in a 100 ml Schlenk flask. The mixture was stirred and cooled to -78 °C, followed by dropwise addition of *n*BuLi in hexane (2.64 M, 1.19 ml, 3.15 mmol). The reaction mixture was warmed to room temperature and stirred for 2 hours. Volatiles were removed under vacuum and the lithiated product was obtained as a yellow solid. The solid was dissolved in diethyl ether (60 ml) and the resulting solution was stirred and cooled to -78 °C. $SiHCl_3$ (0.333 ml, 3.3 mmol) was added, and the reaction mixture was warmed to room temperature and stirred overnight. Volatiles were then removed through vacuum and resulting solid was extracted with hexane. The suspension was filtered to remove LiCl and the filtrate was concentrated and stored at room temperature to obtain **2** as yellow crystals in 71% yield (1.12 g). ^{1}H NMR (C_6D_6, 400 MHz, 25 °C): δ 7.29-7.27 (m, 2H, Ar-H), 7.01-6.97 (m,1H, Ar-H), 6.93 (s, 1H, Si-H), 6.90-6.82 (m, 4H, Ar-H), 6.78-6.74 (m, 1H, Ar-H), 3.39 (sep, 2H, C*H*Me$_2$, *J* = 7.0 Hz), 1.42 (d, 6H, CH(C*H*$_3$)$_2$, *J* = 6.6 Hz), 1.24 (d, 18H, C(C*H*$_3$)$_3$, *J* = 11.8 Hz), 1.02 (d, 6H, CH(C*H*$_3$)$_2$, *J* = 6.6 Hz). ^{13}C{^{1}H} NMR (C_6D_6, 101 MHz, 25 °C): δ 178.03 (d, N*C*N, *J* = 28.7 Hz), 147.42 (Ar-*C*), 133.35 (Ar-*C*), 130.36 (Ar-*C*), 130.32 (Ar-*C*), 129.76 (d, Ar-*C*, *J* = 4.8 Hz), 129.06 (Ar-*C*), 127.54 (Ar-*C*), 124.21 (Ar-*C*), 35.25 (*C*H(CH$_3$)$_2$), 34.99 (*C*H(CH$_3$)$_2$), 29.68 (d, *C*(CH$_3$)$_3$, *J* = 15.8 Hz), 29.19 (C(*C*H$_3$)$_3$), 25.60 (CH(*C*H$_3$)$_2$), 23.62 (CH(*C*H$_3$)$_2$). ^{31}P{^{1}H} NMR (C_6D_6, 162 MHz, 25 °C): δ 98.00 (s). ^{29}Si{^{1}H} (C_6D_6, 79 MHz, 25 °C): δ -70.49 (s). ^{29}Si (C_6D_6, 79 MHz, 25 °C): δ -70.25 (d, *J* = 368.5 Hz). HRMS (ESI): m/z calcd for $C_{27}H_{42}Cl_2N_2PSi$: 523.2232 [(M + H)]$^+$; found: 523.2237.

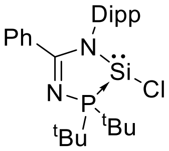

Synthesis of **3.** $LiN(SiMe_3)_2.Et_2O$ (0.579 g, 2.4 mmol) was added to **2** (1.05 g, 2 mmol) in a reaction flask and cooled to -78 °C, followed by addition of toluene (50 ml). The reaction mixture was allowed to warm to room temperature and stirred overnight. The resulting suspension was filtered, and volatiles were removed. Crude product was extracted with heptane and decanted to obtain yellow solids. Yield: 0.891 g (61%). Crystals suitable for X-ray crystallography was afforded from a concentrated hexane solution. ^{1}H NMR (C_6D_6, 400 MHz, 25 °C): δ 7.72-7.69 (m, 2H, Ar-H), 7.14-7.10 (m, 2H, Ar-H), 7.02-7.00 (m, 1H, Ar-H), 6.87 -6.86 (m, 3H, Ar-H), 3.68 (sep, 1H, C*H*Me$_2$, *J* = 6.8 Hz); 3.32 (sep, 1H, C*H*Me$_2$, *J* = 6.8 Hz), 1.54 (d, 3H, CH(C*H*$_3$)$_2$, *J* = 6.8 Hz), 1.48-1.44 (overlapping signals, CH(C*H*$_3$)$_2$ and C(C*H*$_3$)$_3$), 1.28 (d, 3H, CH(C*H*$_3$)$_2$, *J* = 7.0 Hz), 1.24 (d, 9H, C(C*H*$_3$)$_3$, *J* = 14.1 Hz), 0.47 (d, 3H, CH(C*H*$_3$)$_2$, *J* = 6.8 Hz). ^{13}C{^{1}H} NMR (C_6D_6, 101 MHz, 25 °C): δ 172.82 (d, N*C*N, *J* = 4.4 Hz), 146.99 (Ar-*C*), 145.60 (Ar-*C*), 137.59 (d, Ar-*C*, *J* = 9.2 Hz), 136.65 (d, Ar-*C*, *J* = 17.8 Hz), 130.41 (Ar-*C*), 130.01 (Ar-*C*), 127.57 (Ar-*C*), 125.52 (Ar-*C*), 124.84 (Ar-*C*), 39.82 (d, *C*(CH$_3$)$_3$, *J* = 10.5 Hz), 35.43 (d, *C*(CH$_3$)$_3$, *J* = 31.5 Hz), 29.72 (*C*H(CH$_3$)$_2$),

28.51 ($CH(CH_3)_2$), 28.12 ($C(CH_3)_3$), 27.34 ($C(CH_3)_3$), 26.14 ($CH(CH_3)_2$), 25.83 ($CH(CH_3)_2$), 24.49 ($CH(CH_3)_2$), 24.10 ($CH(CH_3)_2$). $^{31}P\{^1H\}$ NMR (C_6D_6, 162 MHz, 25 °C): δ 67.81 (s). $^{29}Si\{^1H\}$ (C_6D_6, 79 MHz, 25 °C): 7.96 (d, *J* = 186.6 Hz). HRMS (ESI): m/z calcd for $C_{27}H_{41}ClN_2PSi$: 487.2465 $[(M + H)]^+$; found: 487.2463.

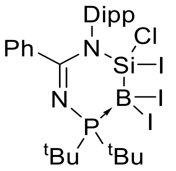

Synthesis of **4**. **3** (0.974 g, 2 mmol) and BI_3 (0.861g, 2.2 mmol) were dissolved in toluene in two separate 100 ml flasks. BI_3 was added to **3** dropwise at -78 °C and the reaction mixture was allowed to warm to room temperature, and heated at 65 °C overnight. Resulting suspension was filtered and the filtrate was concentrated and stored at room temperature to yield colourless crystals**.** Yield: 0.96 g (55%). ^1H NMR (C_6D_6, 400 MHz, 25 °C): δ 7.13- 7.11 (m, 2H, Ar-H), 7.08- 7.06 (m, 2H, Ar-H), 6.92-6.90 (m, 1H, Ar-H), 6.77-6.75 (m, 3H, Ar-H), 4.12 (sep, 1H, C*H*Me$_2$, *J* = 6.6 Hz), 3.27 (sep, 1H, C*H*Me$_2$, *J* = 6.6 Hz), 1.60 (d, 9H, $C(CH_3)_3$, *J* = 14.8 Hz), 1.53 (d, 3H, $CH(CH_3)_2$, *J* = 6.8 Hz), 1.41 (d, 9H, $C(CH_3)_3$, *J* = 15.2 Hz), 1.33 (d, 3H, $CH(CH_3)_2$, *J* = 6.5 Hz), 1.21 (d, 3H, $CH(CH_3)_2$, *J* = 6.7 Hz), 0.24 (d, 3H, $CH(CH_3)_2$, *J* = 6.6 Hz). $^{13}C\{^1H\}$ NMR (C_7D_8, 101 MHz, 25 °C): δ 170.52 (d, N*C*N, *J* = 12.4 Hz), 147.88 (Ar-C), 147.35 (Ar-C), 140.29 (d, Ar-C, *J* = 14.0 Hz), 137.87 (Ar-C), 129.62 (Ar-C), 127.51 (Ar-C), 125.74 (Ar-C), 125.17 (Ar-C), 41.82 (d, $C(CH_3)_3$, *J* = 38.4 Hz), 40.99 (d, $C(CH_3)_3$, *J* = 36.2 Hz), 29.38 ($CH(CH_3)_2$), 29.08 ($C(CH_3)_3$), 28.69 ($C(CH_3)_3$), 26.77 ($CH(CH_3)_2$), 25.37 ($CH(CH_3)_2$), 25.07 ($CH(CH_3)_2$), 22.92 ($CH(CH_3)_2$). $^{31}P\{^1H\}$ NMR (C_7D_8, 162 MHz, 25 °C): δ 26.57 (d, *J* = 95.1 Hz). $^{11}B\{^1H\}$ NMR (C_7D_8, 128 MHz, 25 °C): δ -45.57 (d, *J* = 70.7 Hz). $^{29}Si\{^1H\}$ NMR (C_4D_8O, 79 MHz, 25 °C): δ -7.28 – -10.37 (m). $^{11}B\{^1H\}$ NMR (C_4D_8O, 128 MHz, 25 °C): δ -45.53 (d, *J* = 55.0 Hz). HRMS (ESI): m/z calcd for $C_{27}H_{41}BClI_3N_2PSi$: 878.9692 $[(M + H)]^+$; found: 878.9697.

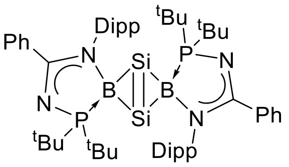

Synthesis of **5**. THF (20 ml) was added to a 100 ml flask containing **4** (0.439 g, 0.5 mmol) and excess KC_8 (0.338 g, 2.5 mmol) at -78 °C. The reaction mixture was allowed to warm to room temperature and stirred for 3 hours. The resulting suspension was filtered and volatiles in the filtrate were removed. The crude solid was extracted with toluene and the solution was concentrated to yield black crystals. Yield: 0.023 g (10%).^1H NMR (THF-*d*$_8$, 400 MHz, 25 °C): δ 7.48 – 7.36 (m, 4H, Ar-H), 7.16 (m, 2H, Ar-H), 7.00 (m, 6H, Ar-H), 6.86 (m, 4H, Ar-H), 3.34 (sep, 4H, C*H*Me$_2$, *J* = 6.8 Hz), 1.35 – 1.25 (m, 36H, $C(CH_3)_3$), 1.19 (d, 6H, $CH(CH_3)_2$, *J* = 6.8 Hz), 0.54 (d, 6H, $CH(CH_3)_2$, *J* = 6.8 Hz). $^{13}C\{^1H\}$ NMR (THF-*d*$_8$, 101 MHz, 25 °C): δ 175.05 (N*C*N), 145.72 (Ar-C), 132.02 (Ar-C), 130.30 (Ar-C), 127.70 (Ar-C), 127.46 (Ar-C), 124.73 (Ar-C), 36.04 (d, $C(CH_3)_3$, *J* = 33.9 Hz), 29.31 ($CH(CH_3)_2$), 28.41 ($C(CH_3)_3$), 25.66 ($CH(CH_3)_2$), 25.46 ($CH(CH_3)_2$), 25.26 ($CH(CH_3)_2$). $^{31}P\{^1H\}$ NMR (THF-*d*$_8$, 162 MHz, 25 °C): δ 40.93 (br). $^{11}B\{^1H\}$ (THF-*d*$_8$, 128 MHz, 25 °C): δ 30.46 (d, *J* = 110.5 Hz). $^{29}Si\{^1H\}$ (THF-*d*$_8$, 79 MHz, 25 °C): δ 232.99 (dd, *J* = 10.3, 11.9 Hz). HRMS (ESI): m/z calcd for $C_{54}H_{81}B_2N_4P_2Si_2$: 925.5661 $[(M + H)]^+$; found: 925.5680.

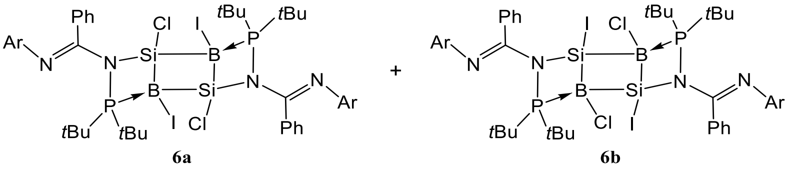

Synthesis of **6a** and **6b**. Toluene (20 ml) was added to a 100 ml flask containing **4** (0.439 g, 0.5 mmol) and excess KC_8 (0.338 g, 2.5 mmol) at -78 °C. The reaction mixture was stirred for 16 hours at room temperature and the resulting suspension was filtered to yield a dark brown solution, which was concentrated to yield colourless crystals of **6a** and **6b**. Yield: 0.033 g (10%). $^{31}P\{^1H\}$ solid state NMR (202 MHz, 25 °C): δ 108.43, 103.44. $^{11}B\{^1H\}$ solid state NMR (160 MHz, 25 °C) : δ -11.29, -34.80. ^1H NMR (THF-*d*$_8$, 400 MHz, 25 °C): δ 7.55 (br, 1H, Ar-H), 7.51-7.50 (m, 1H, Ar-H), 7.46-7.43 (m, 1H, Ar-H), 7.18-7.16 (m, 1H, Ar-H), 7.13-7.11 (m, 3H, Ar-H), 7.07-7.05 (m, 3H, Ar-H),

6.96-6.91 (m, 1H, Ar-H), 6.86-6.84 (m, 1H, Ar-H), 6.78-6.72 (m, 4H, Ar-H), 3.50-3.48 (overlapping signals, 1H, C*H*Me2), 3.36-3.28 (m, 1H, C*H*Me2), 3.19-3.09 (m, 1H, C*H*Me2), 2.96-2.84 (m, 1H, C*H*Me2), 1.87-1.82 (m, C(C*H*3)3, 17H), 1.78 (d, 6H, C(C*H*3)3, *J* = 14.5 Hz), 1.62 (d, 9H, C(C*H*3)3, *J* = 16.2 Hz), 1.31-1.26 (m, overlapping signals, C(C*H*3)3 and CH(C*H*3)2, 6H), 1.16 (d, 3H, CH(C*H*3)2, *J* = 6.3 Hz), 1.07 (d, 4H, CH(C*H*3)2, *J* = 6.3 Hz), 1.02 (d, 3H, CH(C*H*3)2, *J* = 7.3 Hz), 1.00 – 0.97 (m, 4H, CH(C*H*3)2), 0.91 (d, 2H, CH(C*H*3)2, *J* = 6.3 Hz), 0.88 (d, 3H, CH(C*H*3)2, *J* = 7.7 Hz), 0.82 (d, 2H, CH(C*H*3)2, *J* = 6.7 Hz), 0.68 (d, 1H, CH(C*H*3)2, *J* = 6.9 Hz). HRMS (ESI): m/z calcd for $C_{54}H_{81}B_2Cl_2I_2N_4P_2Si_2$: 1249.3128 [(M + H)]$^+$; found: 1249.3149.

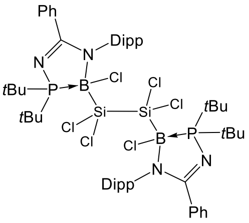

Synthesis of **7**. THF (0.4 ml) was added to a JYoung containing **5** (0.0185 g, 0.02 mmol) and C_2Cl_6 (0.0047 g, 0.02 mmol) at rt, and stirred for 20 mins to afford a mixture of compounds (according to in situ $^{31}P\{^1H\}$ NMR). The resulting suspension was filtered using a syringe filter to yield a few colourless crystals of **7**. Crude NMR: $^{31}P\{^1H\}$ NMR (THF, 162 MHz, 25 °C): δ 76.16 (br).

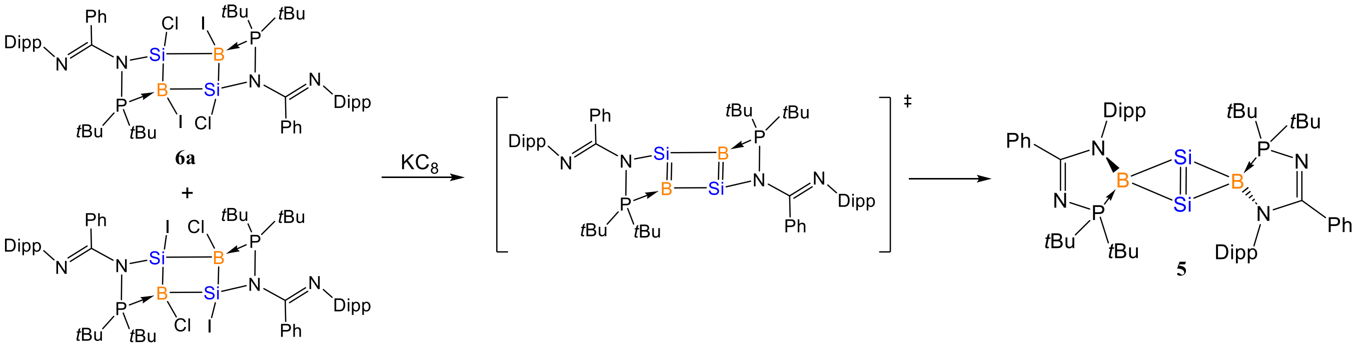

Scheme S1. Proposed mechanism for the formation of compound **5** from **6a** and **6b**

S2. Selected NMR spectra

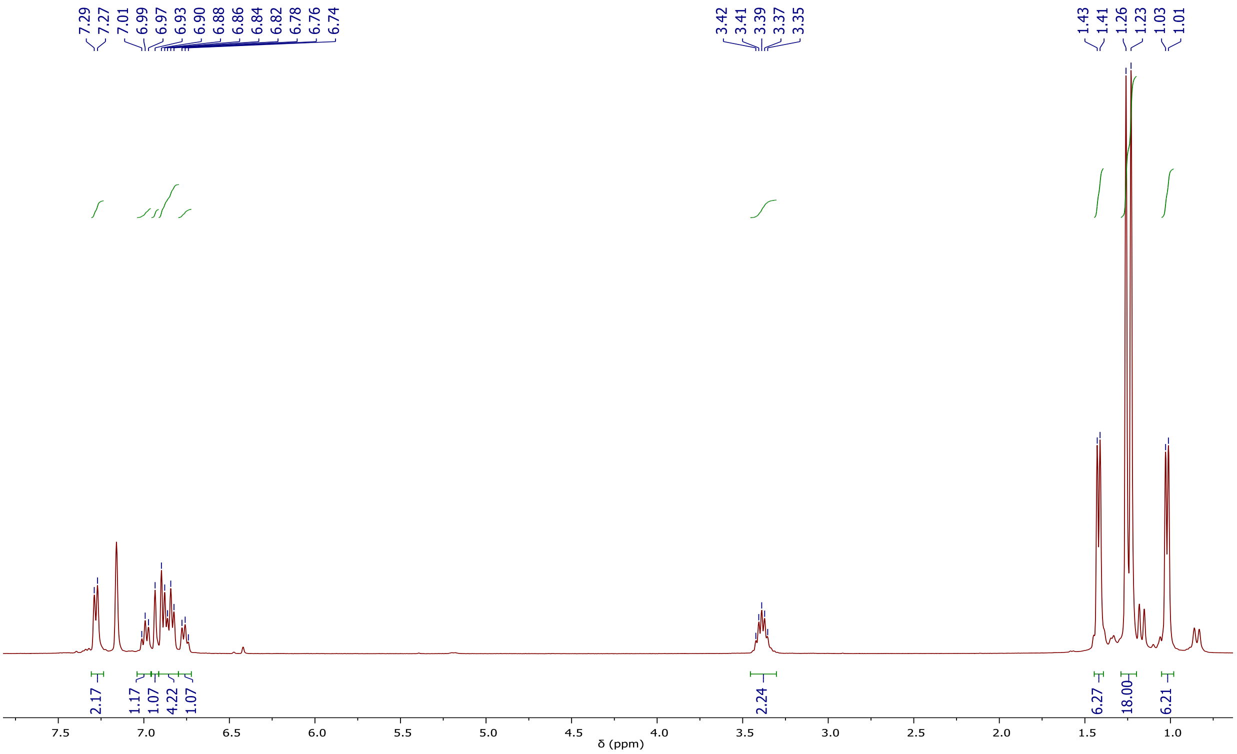

Figure S1. ^{1}H NMR spectrum of **2**

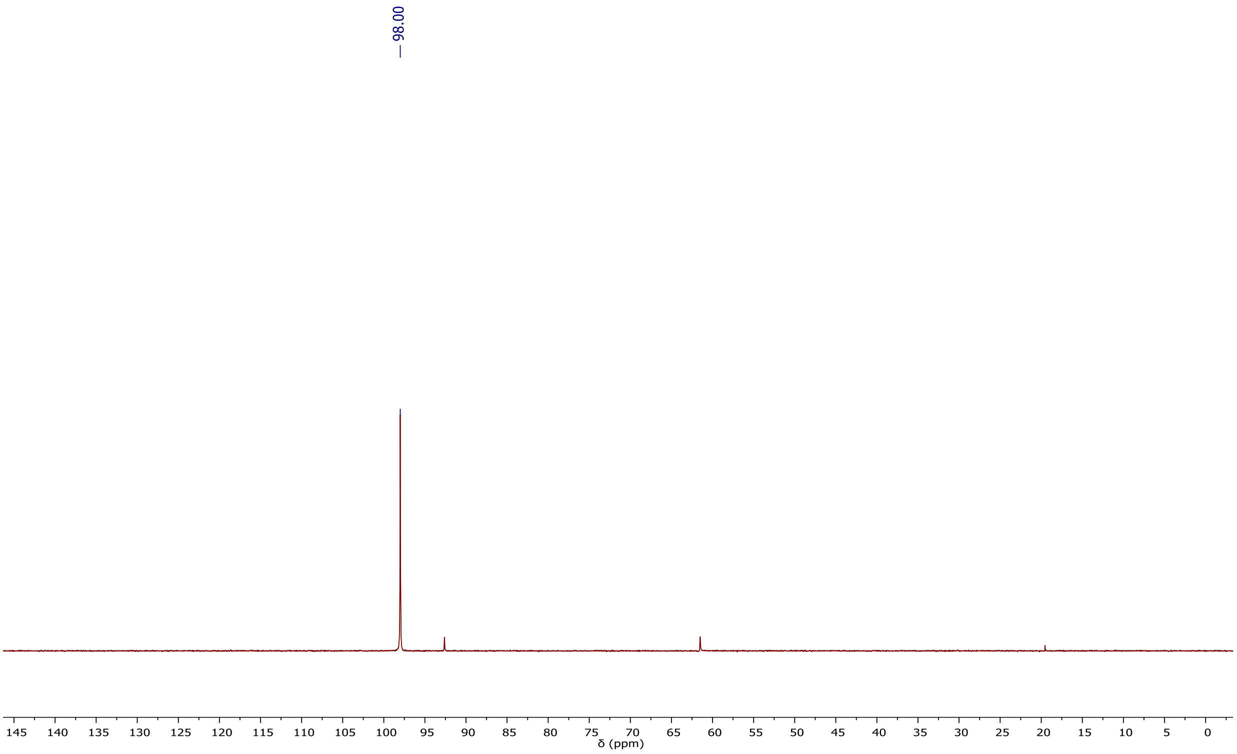

Figure S2. $^{31}P\{^{1}H\}$ NMR spectrum of **2**

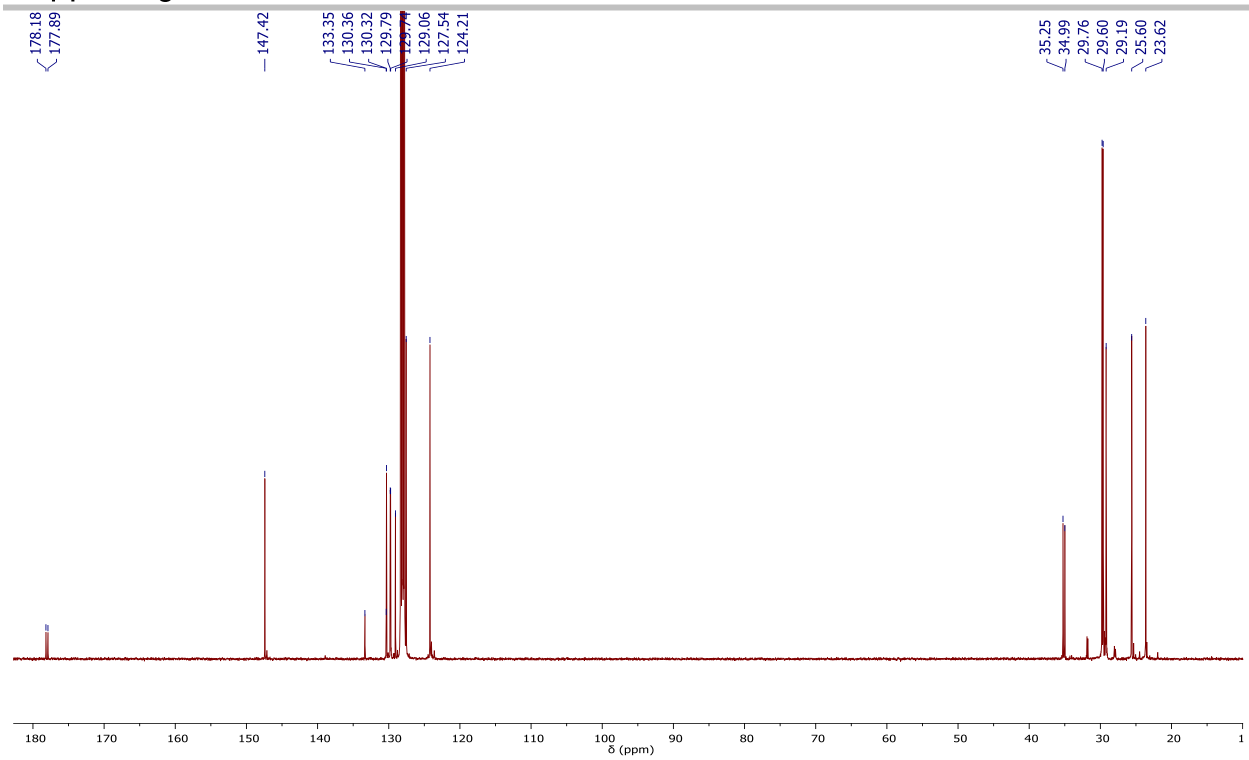

Figure S3. $^{13}C\{^{1}H\}$ NMR spectrum of **2**

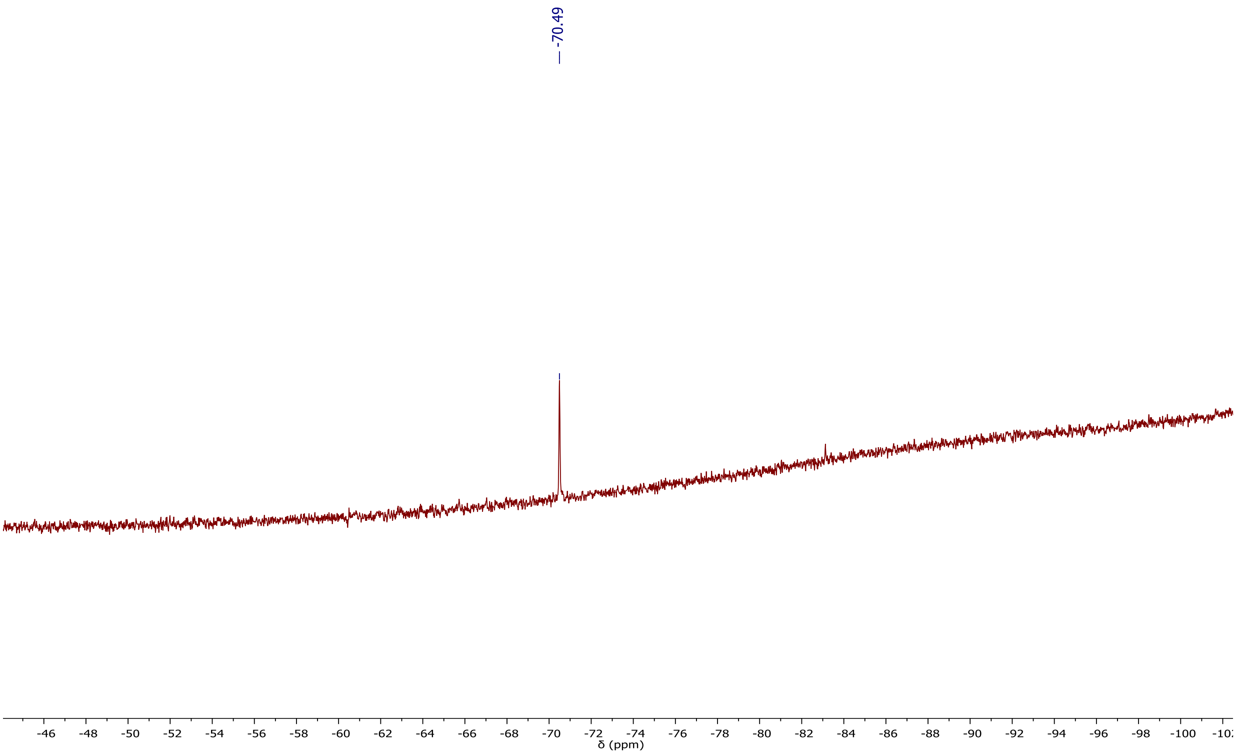

Figure S4. $^{29}Si\{^{1}H\}$ NMR spectrum of **2**

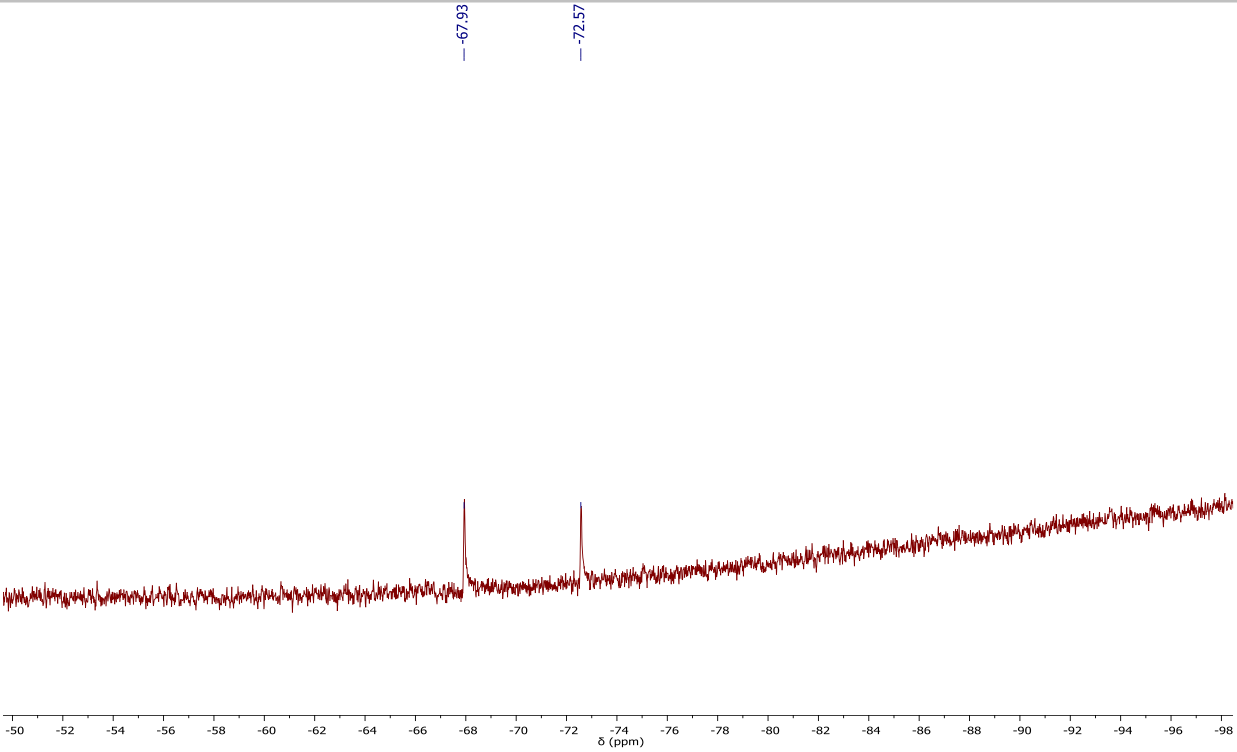

Figure S5. ^{29}Si NMR spectrum of **2**

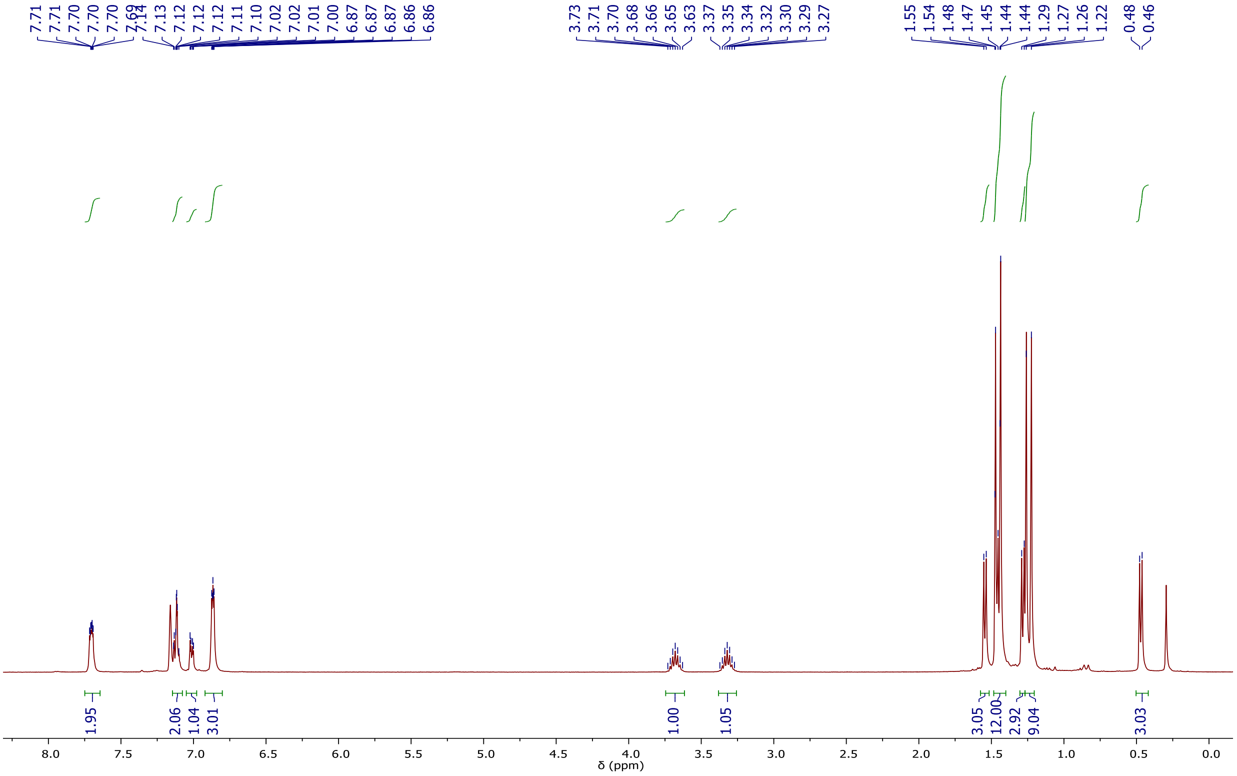

Figure S6. ^{1}H NMR spectrum of **3**

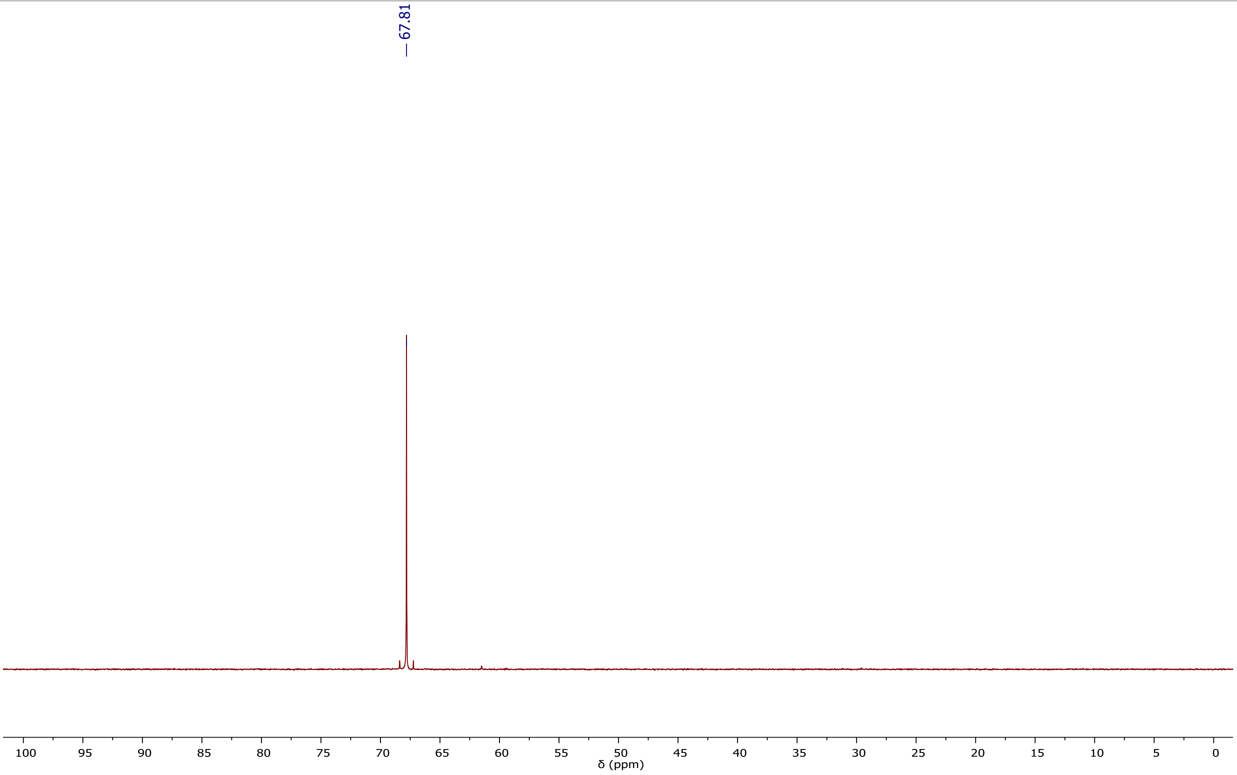

Figure S7. $^{31}P\{^{1}H\}$ NMR spectrum of **3**

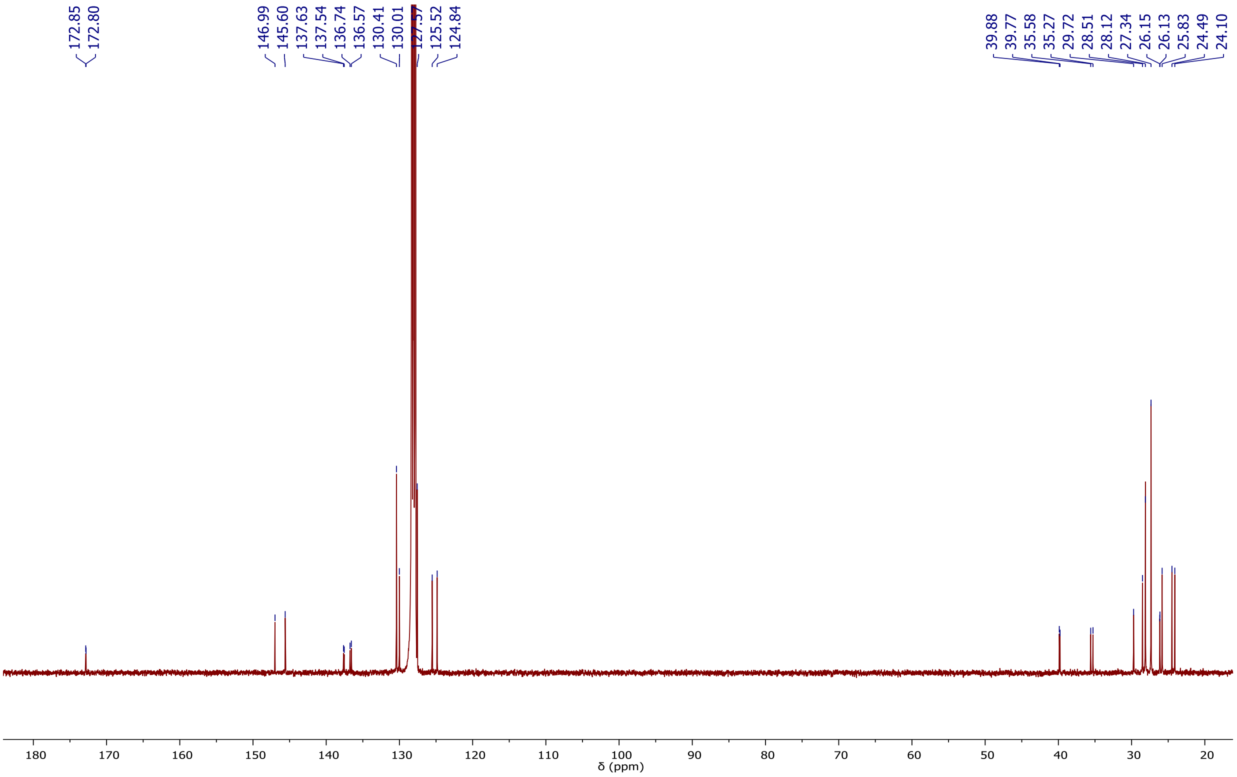

Figure S8. $^{13}C\{^{1}H\}$ NMR spectrum of **3**

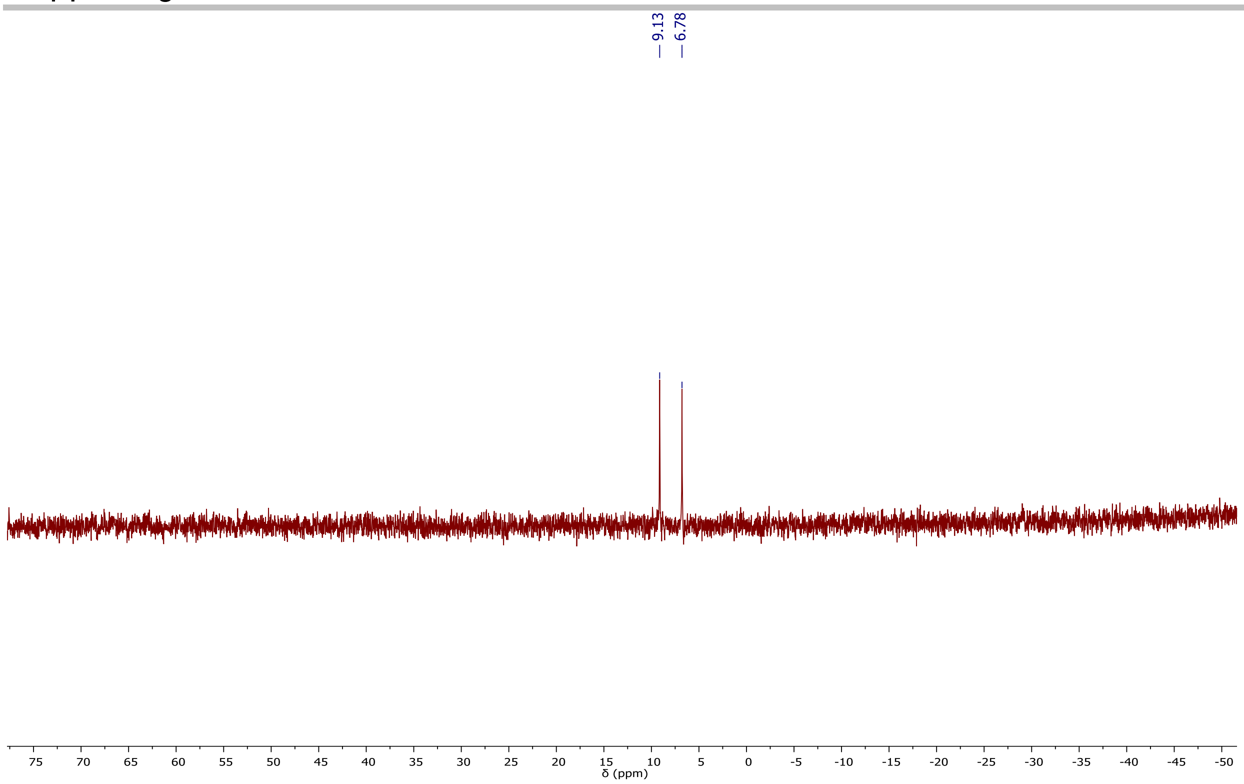

Figure S9. $^{29}Si\{^{1}H\}$ NMR spectrum of **3**

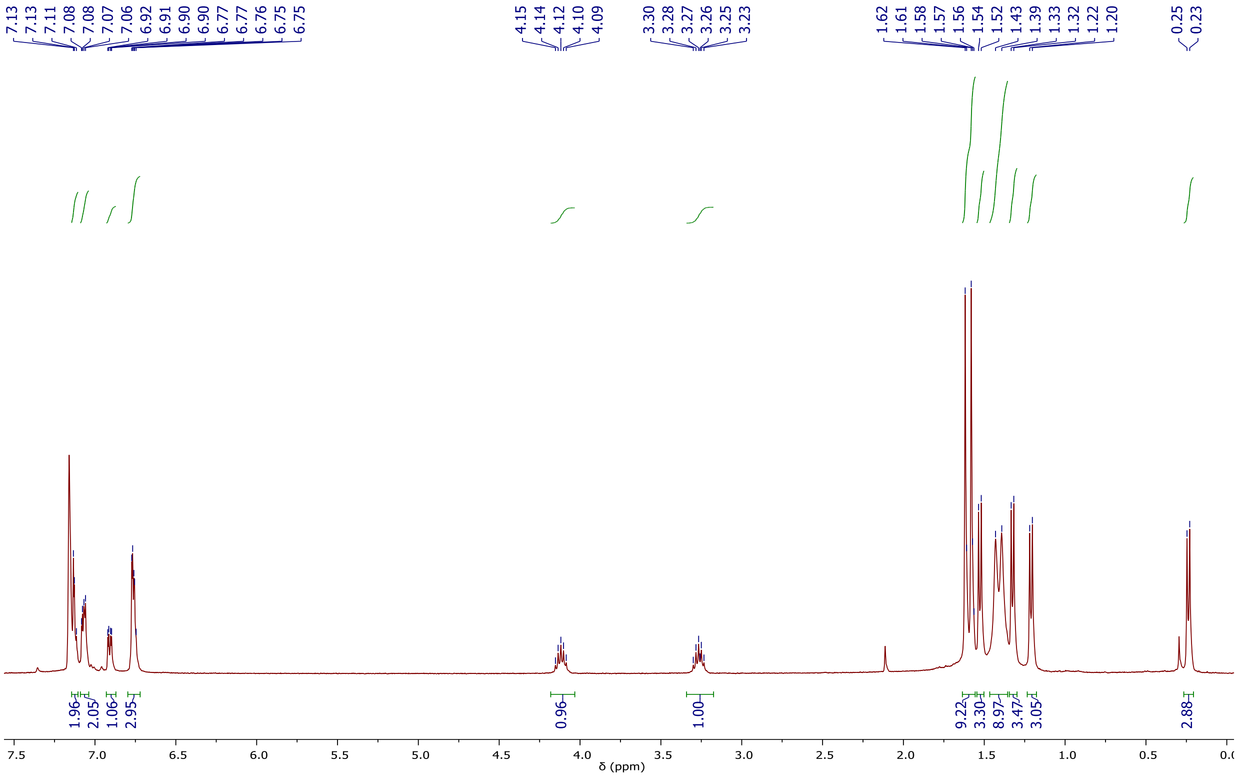

Figure S10. ^{1}H NMR spectrum of **4** in C_6D_6

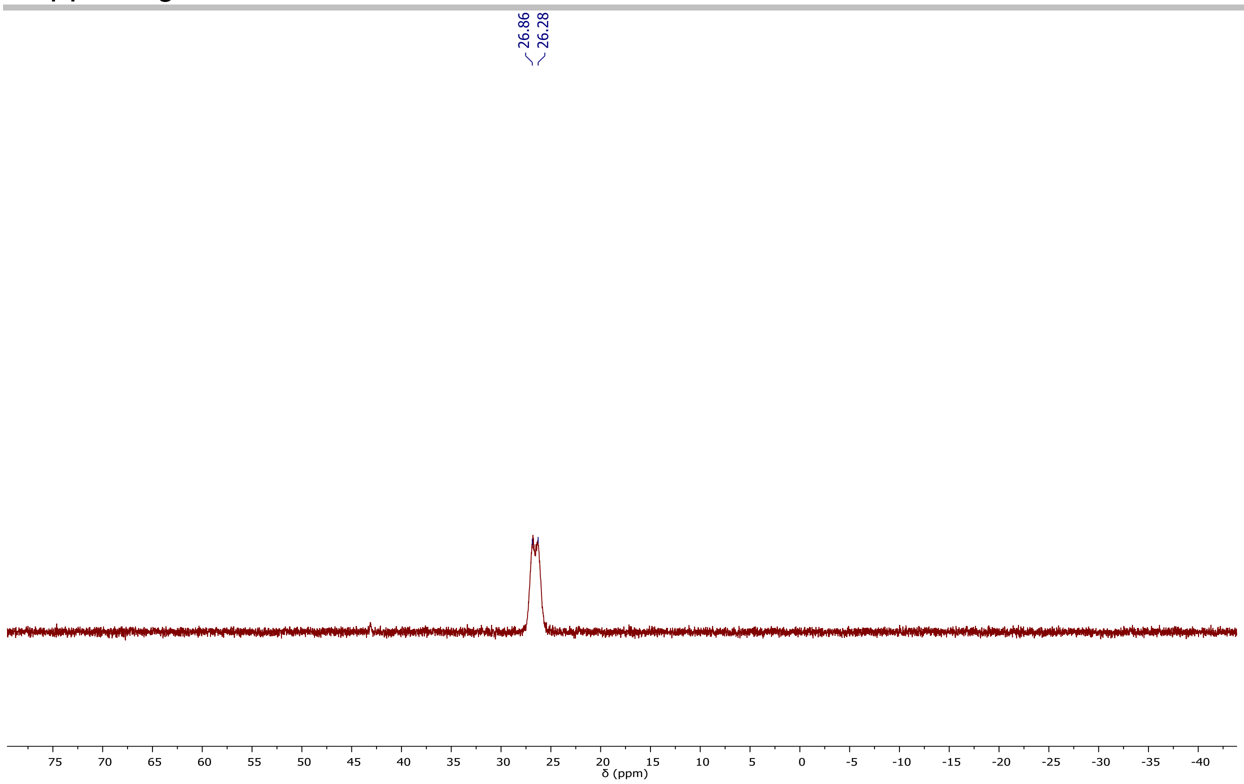

Figure S11. $^{31}P\{^{1}H\}$ NMR spectrum of **4** in toluene-d_8

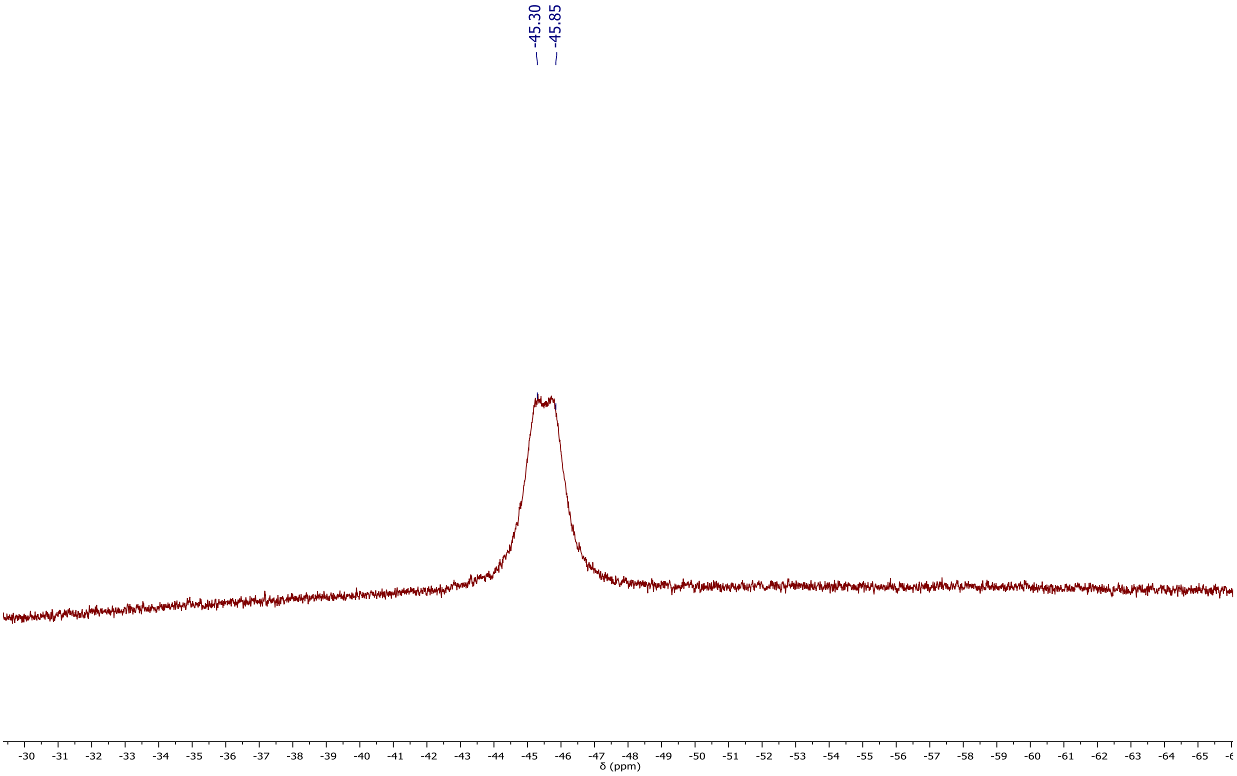

Figure S12. $^{11}B\{^{1}H\}$ NMR spectrum of **4** in toluene-d_8

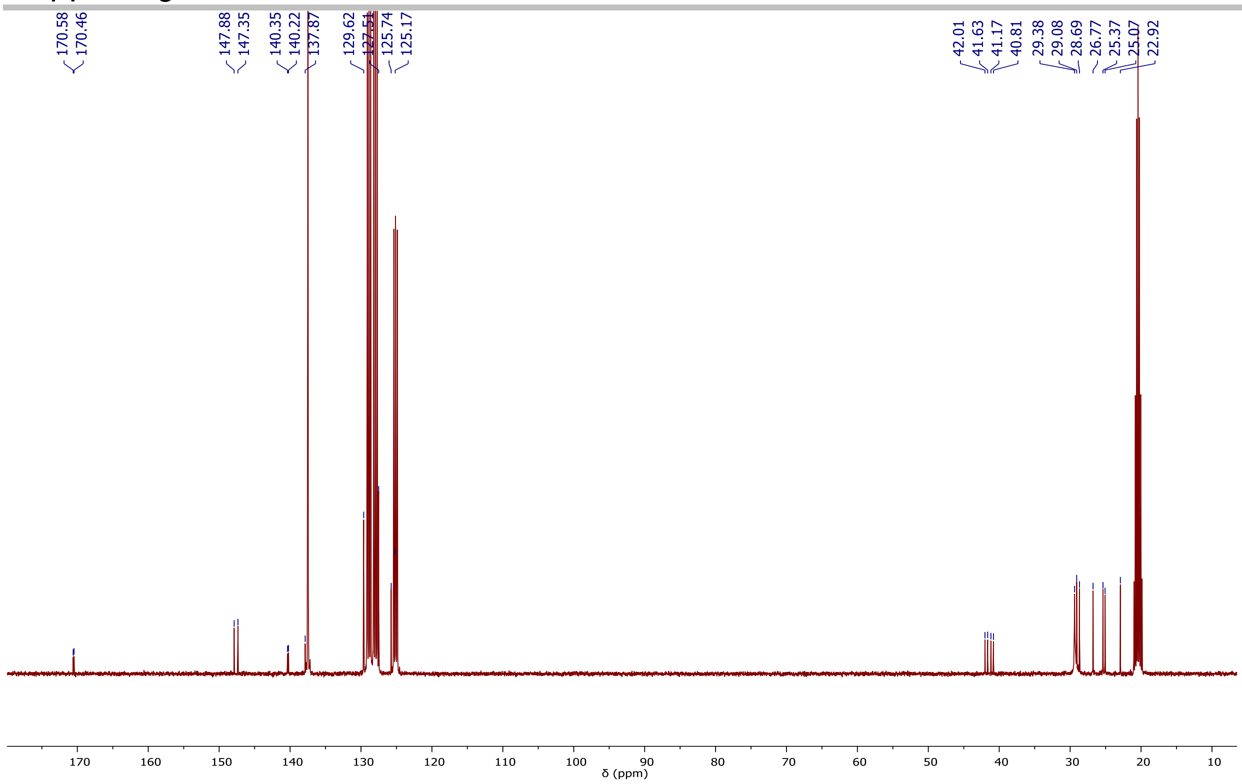

Figure S13. $^{13}C\{^{1}H\}$ NMR spectrum of **4** in toluene-d_8

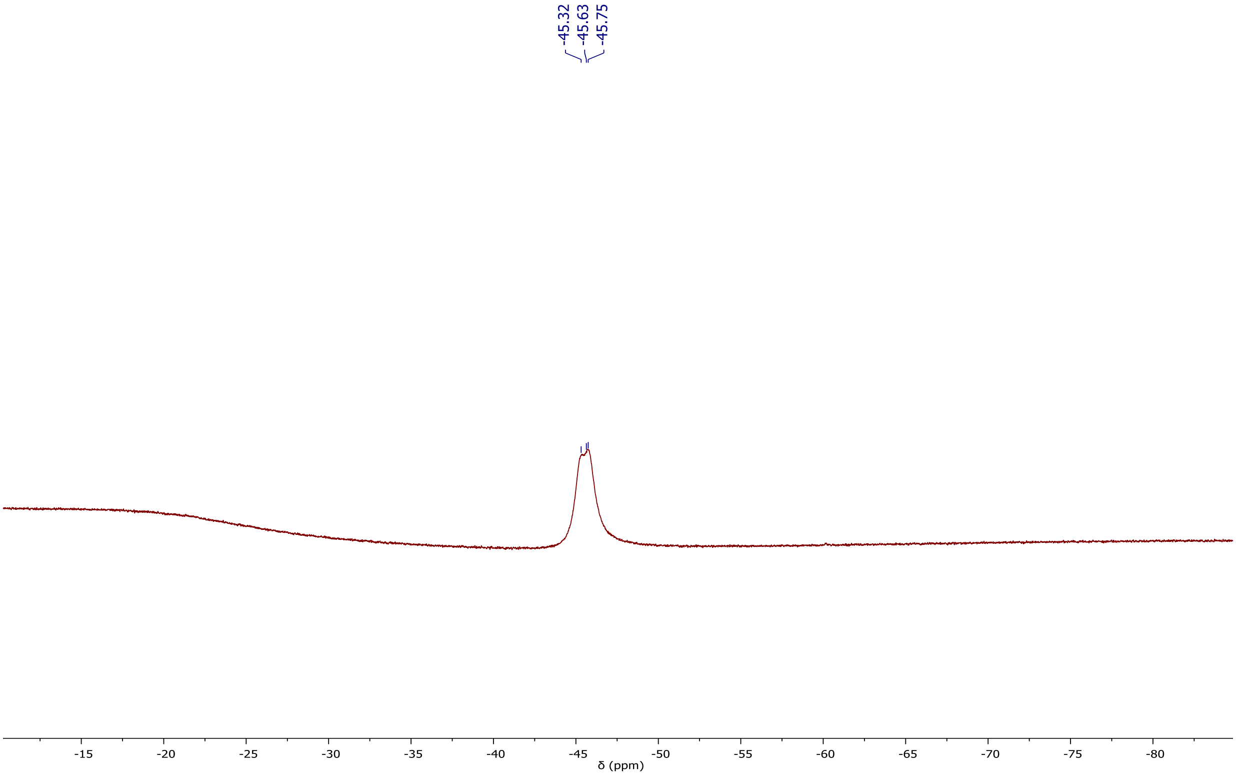

Figure S14. $^{11}B\{^{1}H\}$ NMR spectrum of **4** in THF-d_8

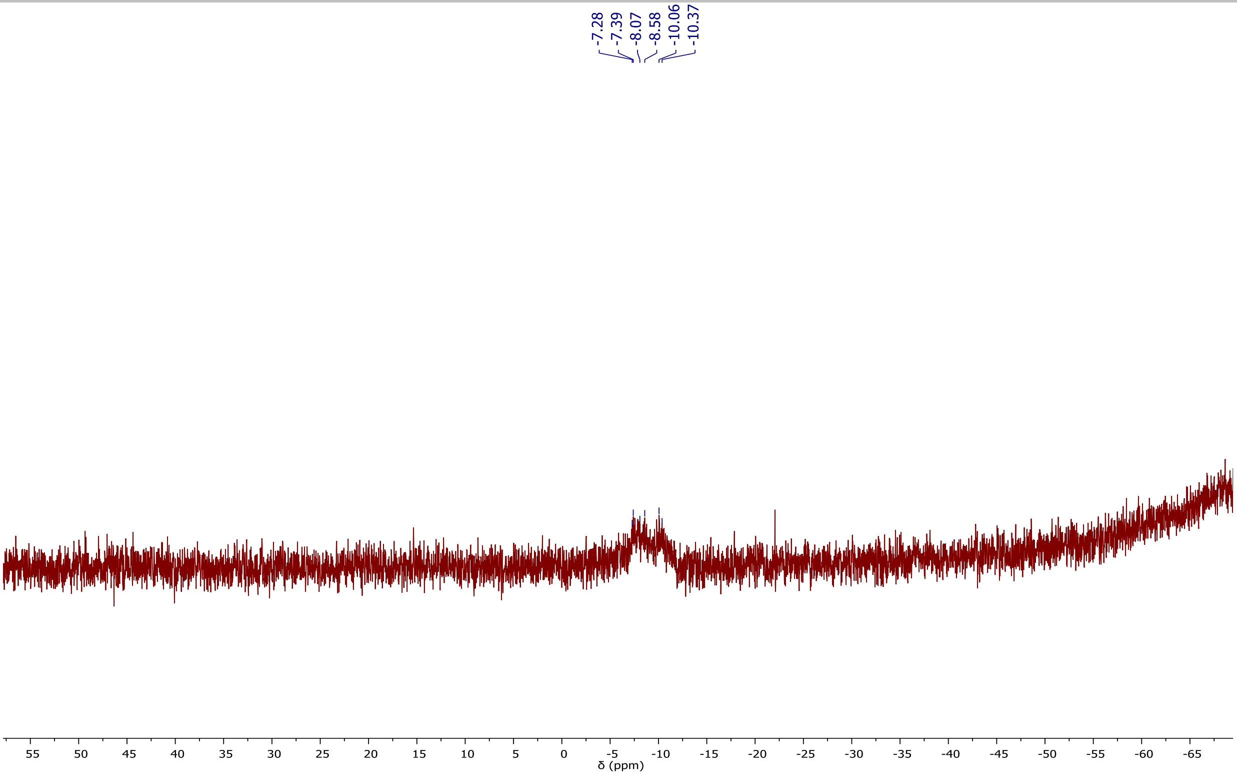

Figure S15. $^{29}Si\{^{1}H\}$ NMR spectrum of **4** in THF-d_8

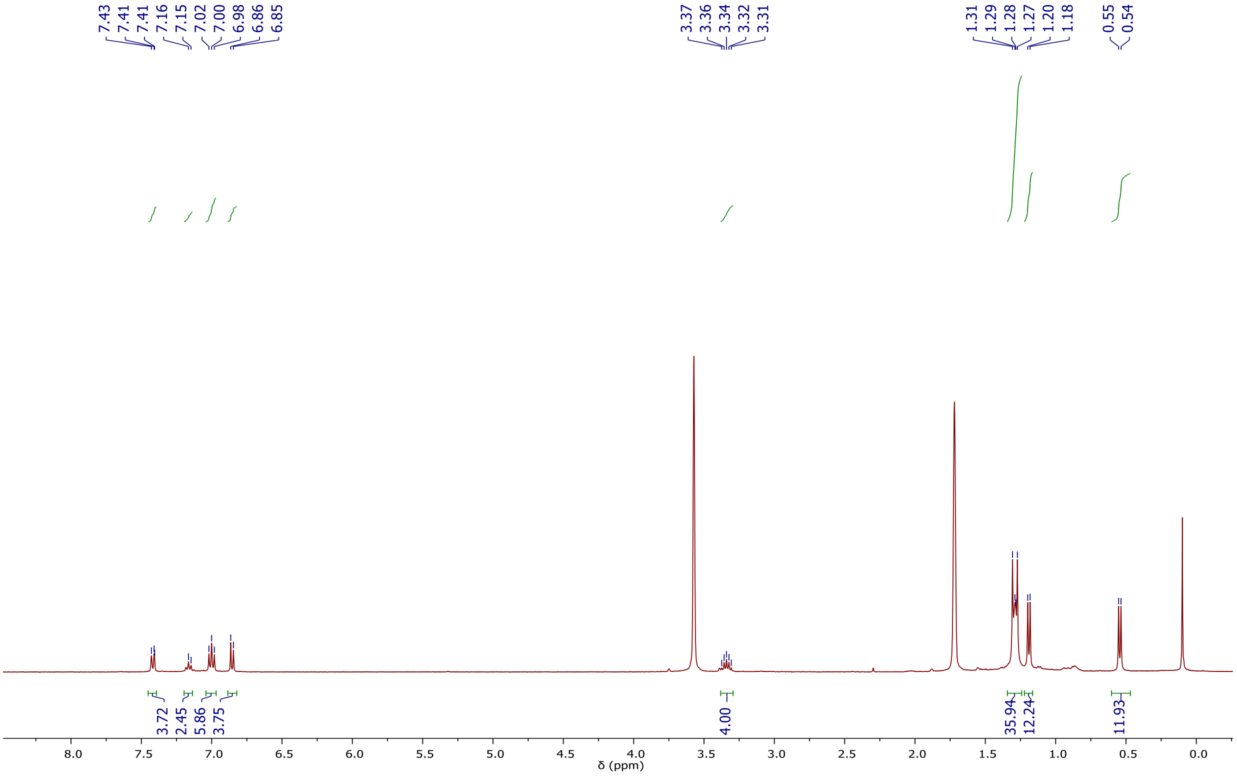

Figure S16. ^{1}H NMR spectrum of **5** in THF-d_8

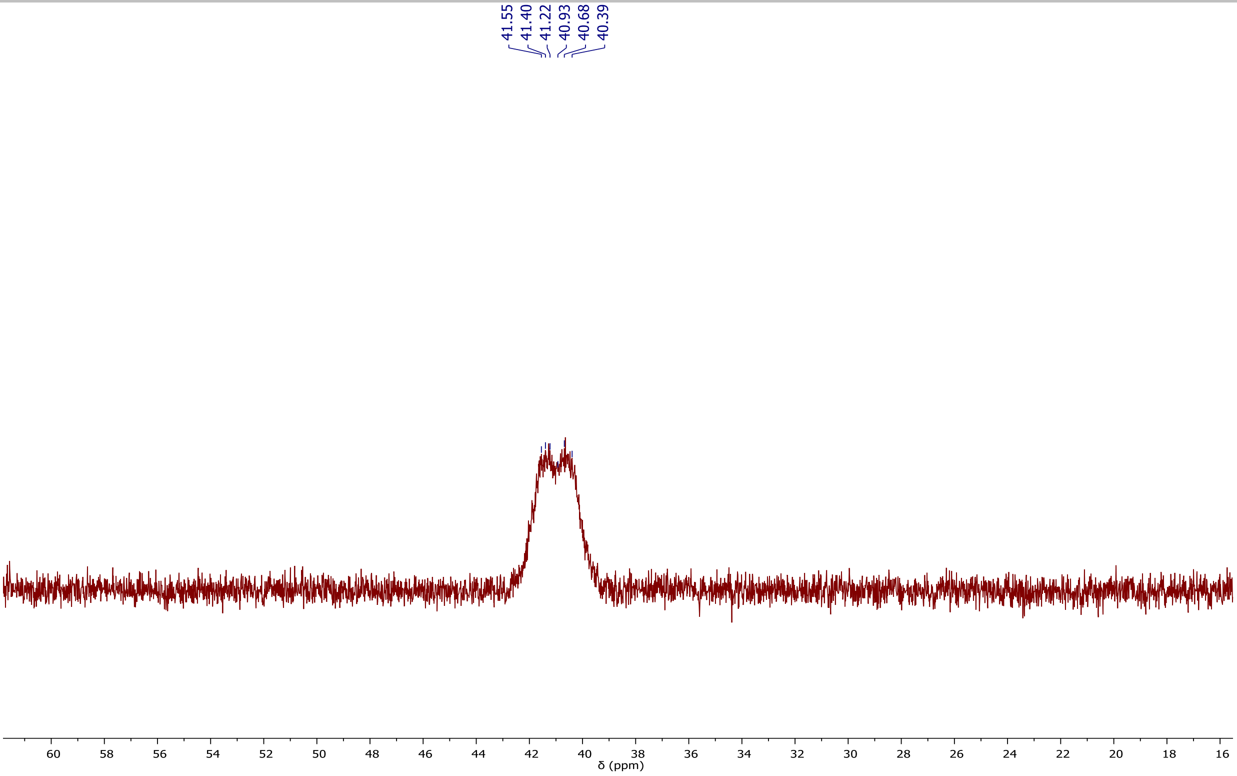

Figure S17. $^{31}P\{^{1}H\}$ NMR spectrum of **5** in THF-d_8

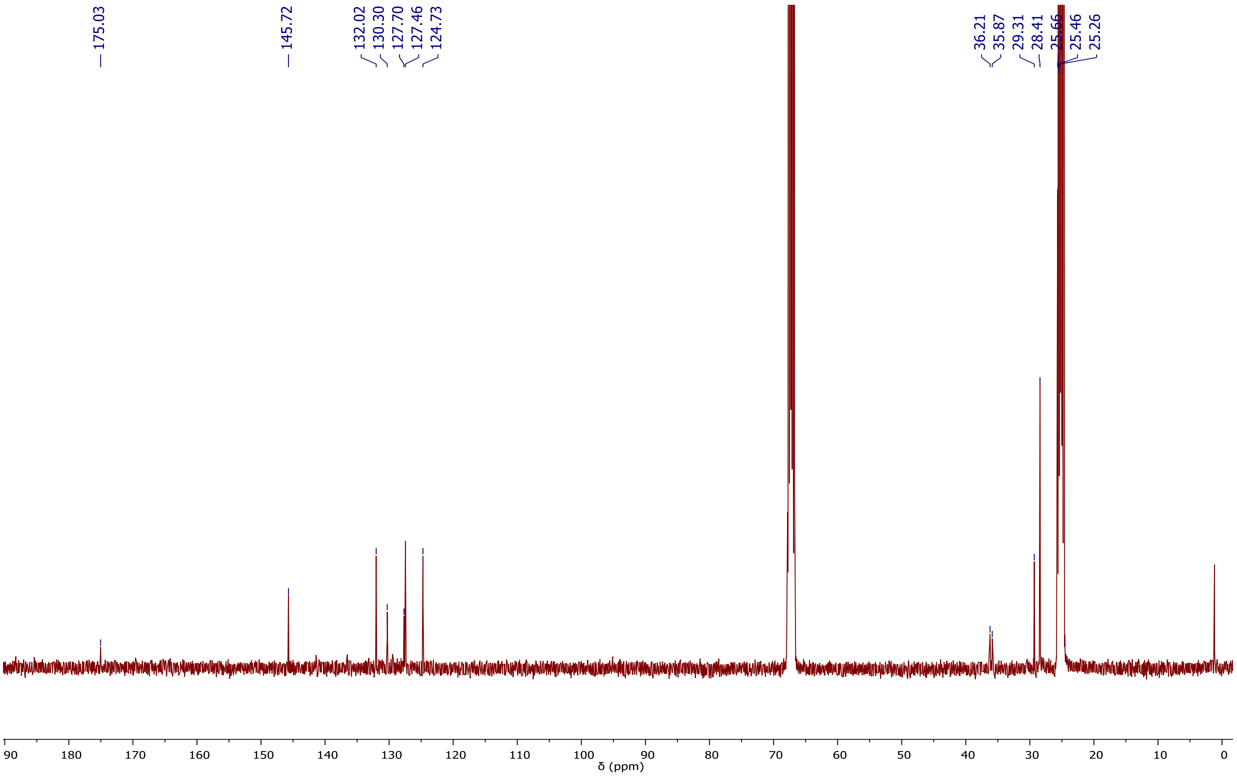

Figure S18. $^{13}C\{^{1}H\}$ NMR spectrum of **5** in THF-d_8

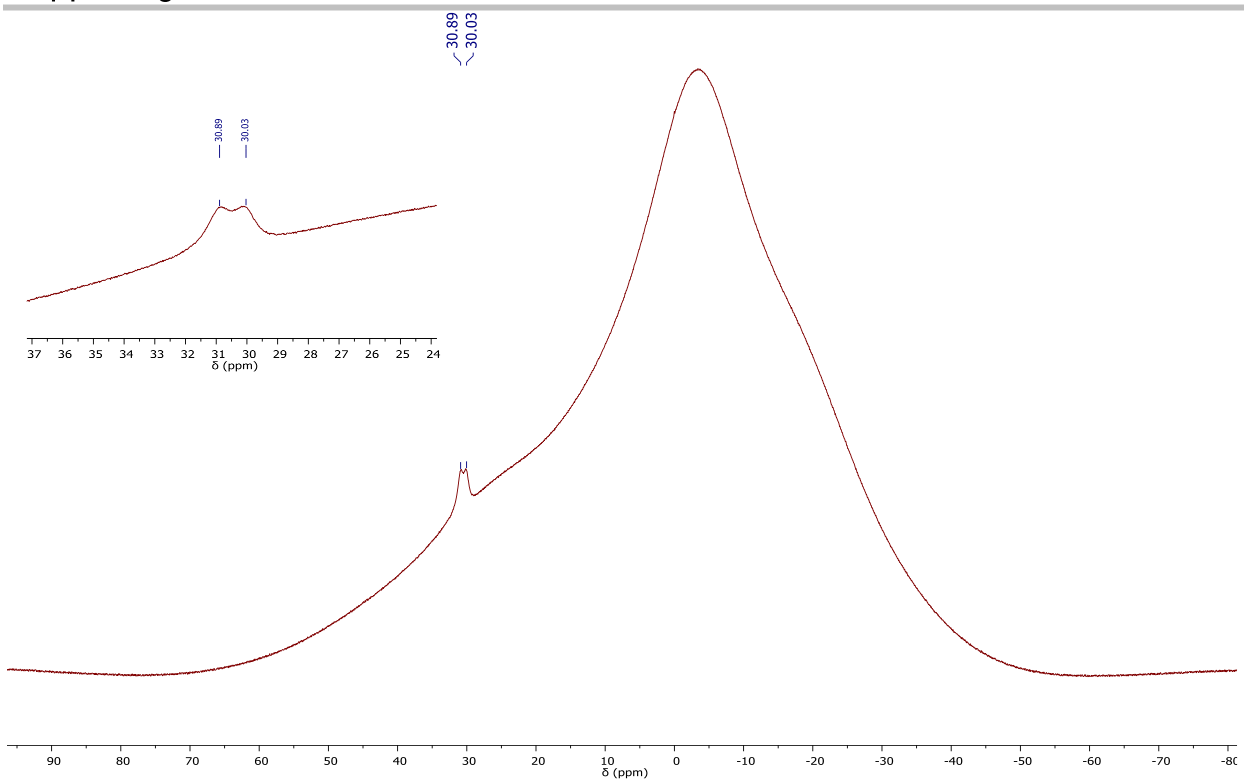

Figure S19. $^{11}B\{^{1}H\}$ NMR spectrum of **5** in THF-d_8

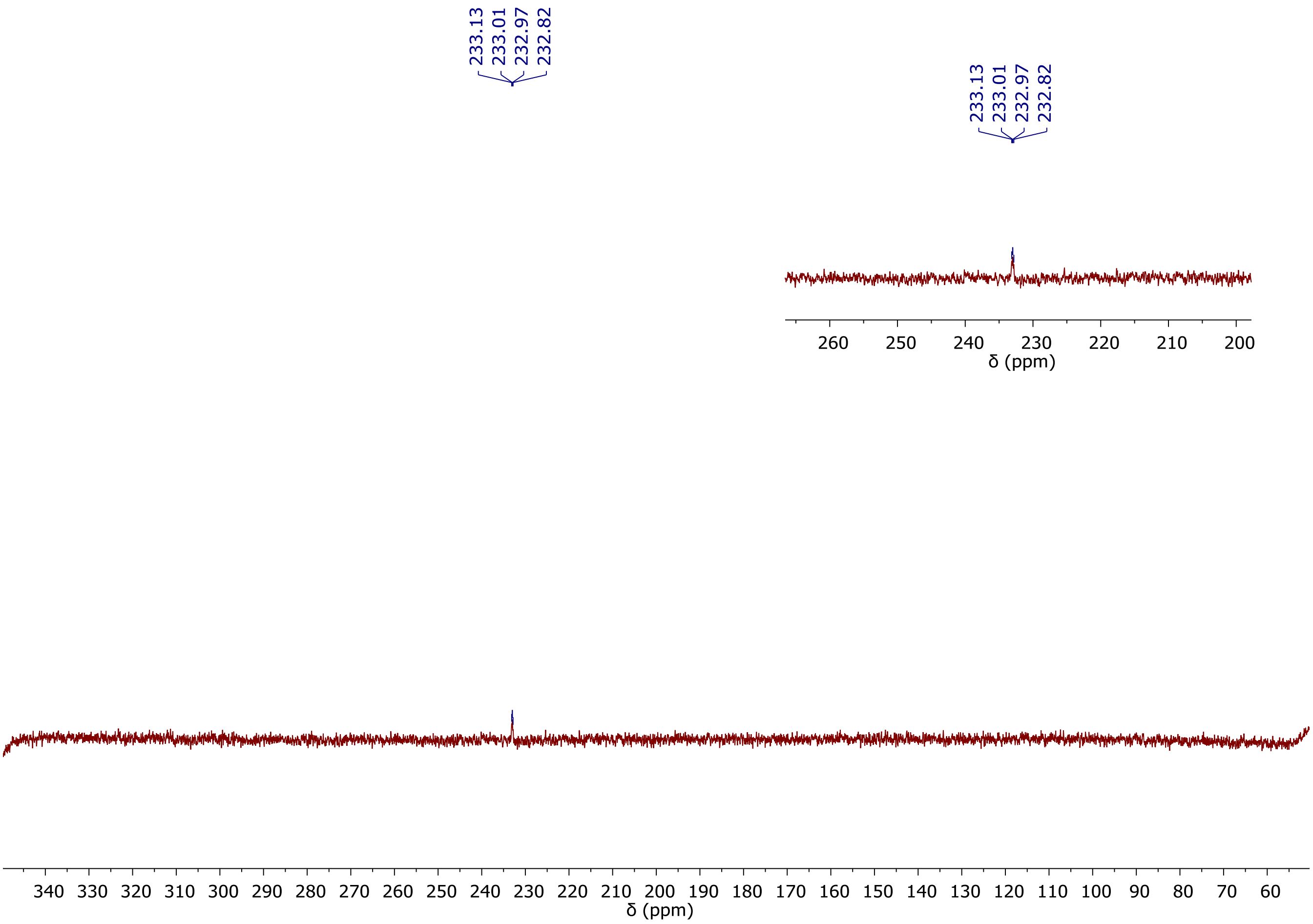

Figure S20. $^{29}Si\{^{1}H\}$ NMR spectrum of **5** in THF-d_8

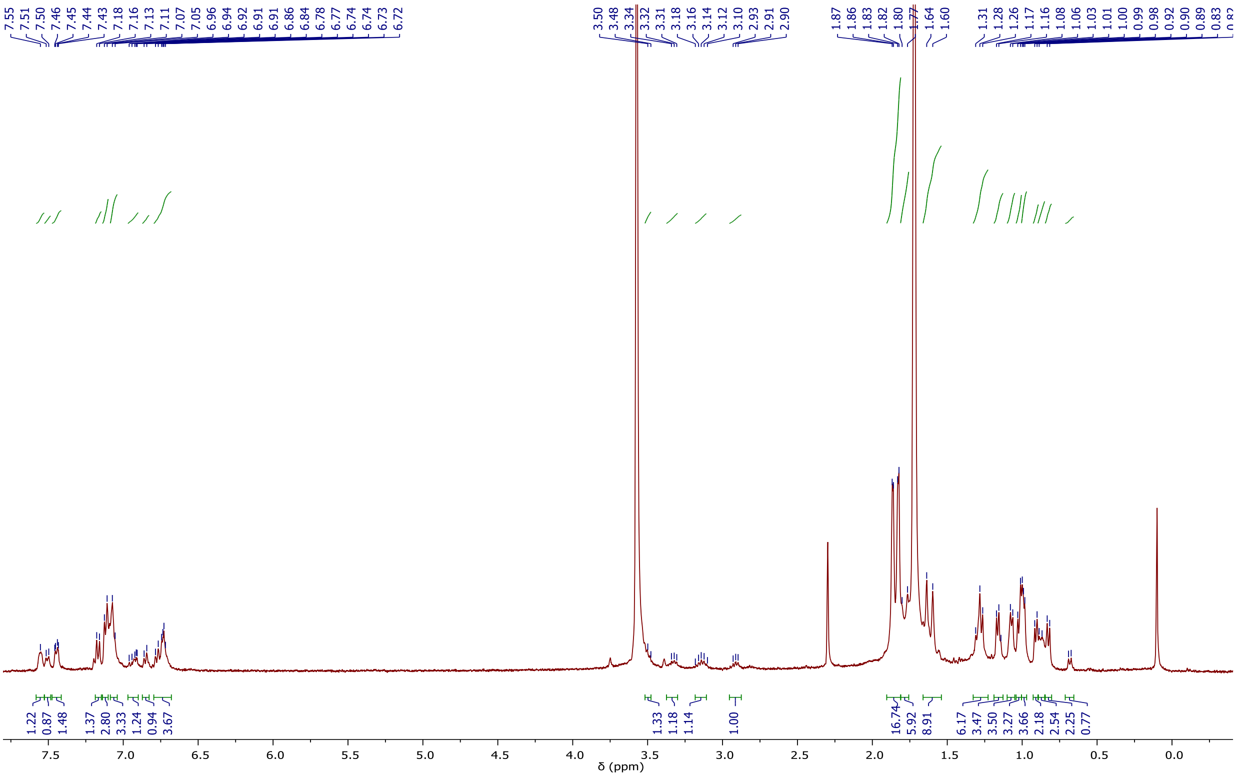

Figure S21. ^{1}H NMR spectrum of **6a** and **6b** in THF-d_8

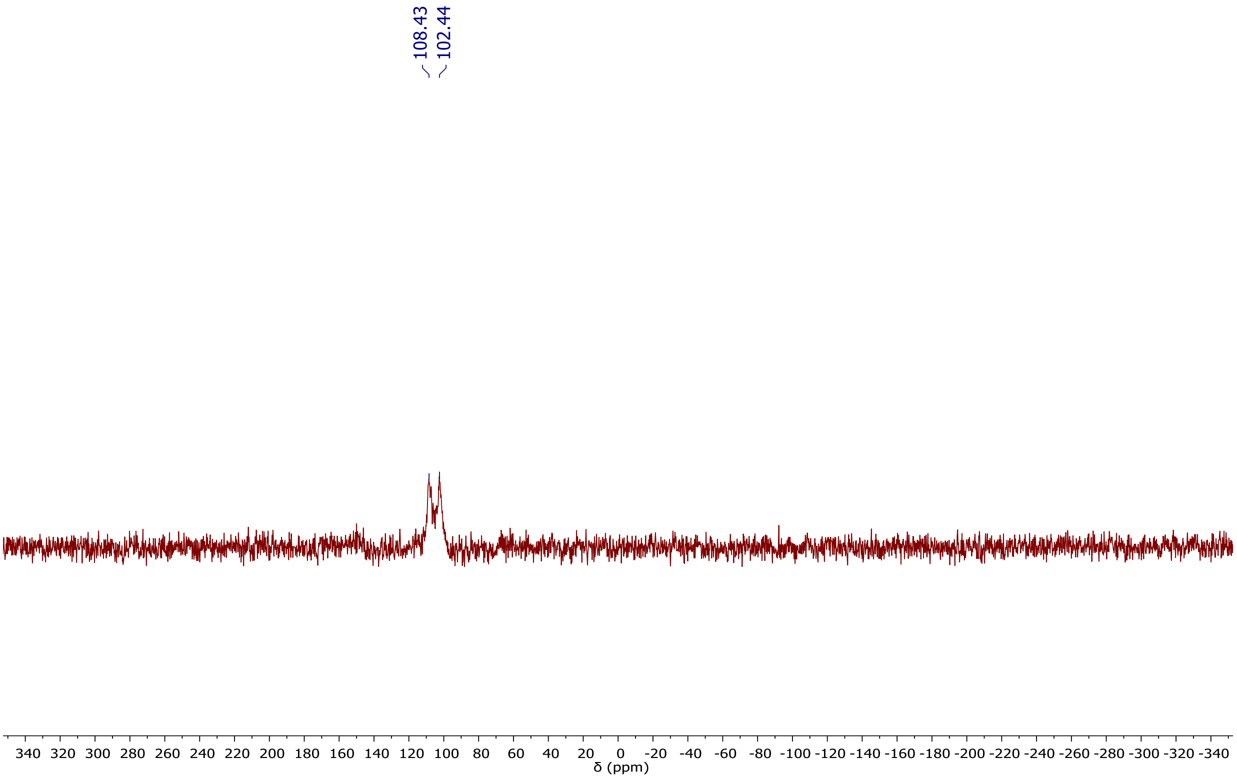

Figure S22. $^{31}P\{^{1}H\}$ solid state NMR spectrum of **6a** and **6b**

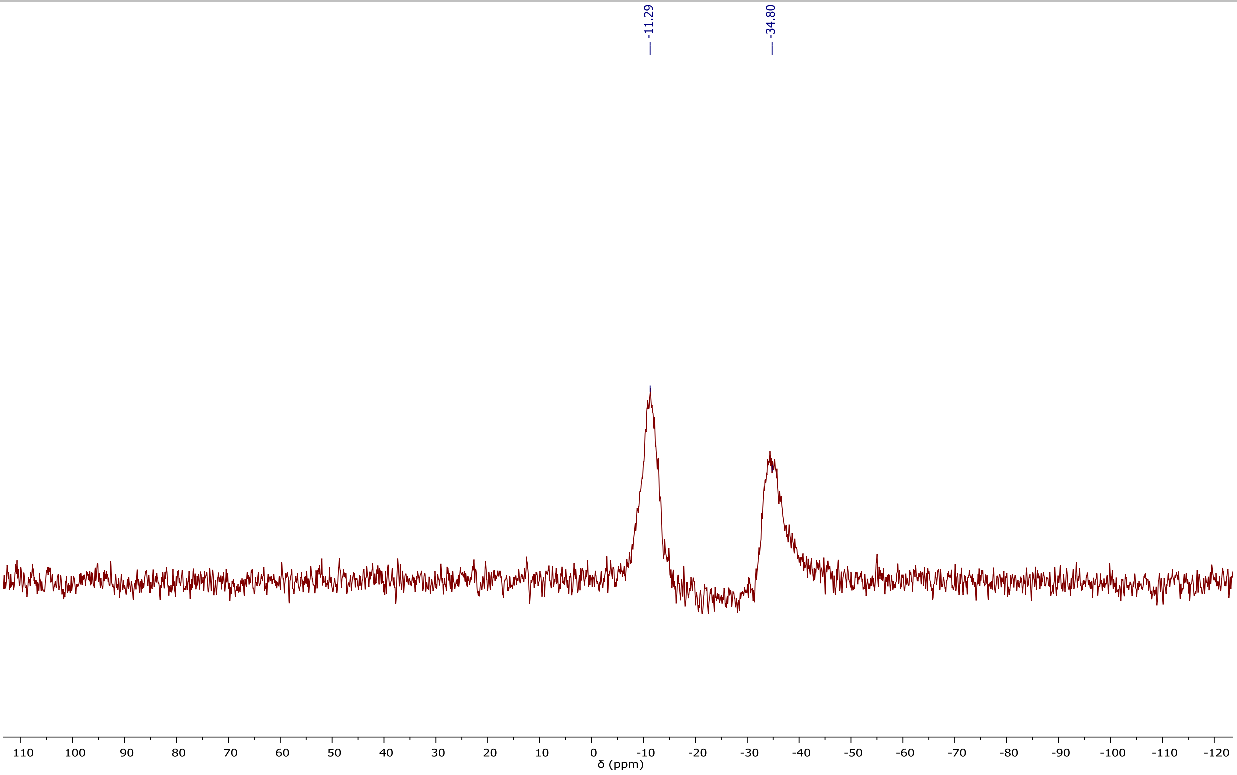

Figure S23. $^{11}B\{^{1}H\}$ solid state NMR spectrum of **6a** and **6b**

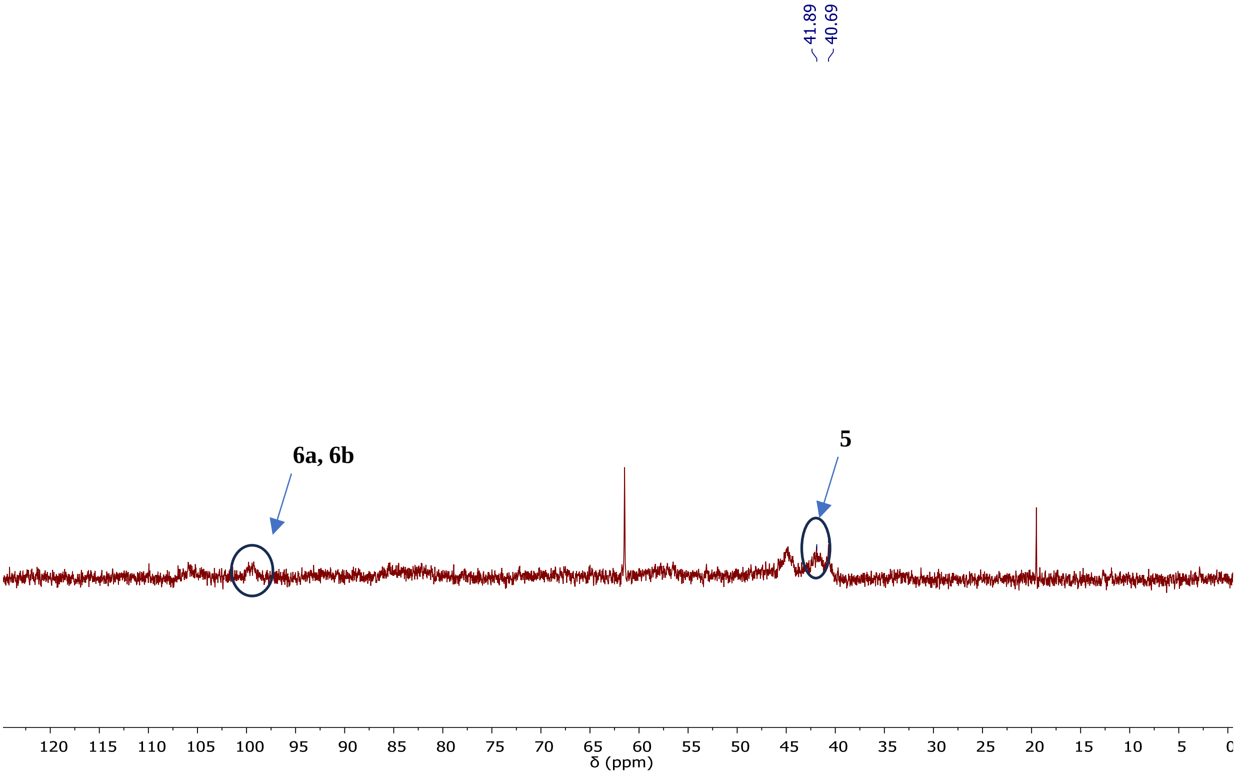

Figure S24. In situ $^{31}P\{^{1}H\}$ NMR spectrum of the reduction of **4** in toluene after 3 days, shows the presence of compound **5**

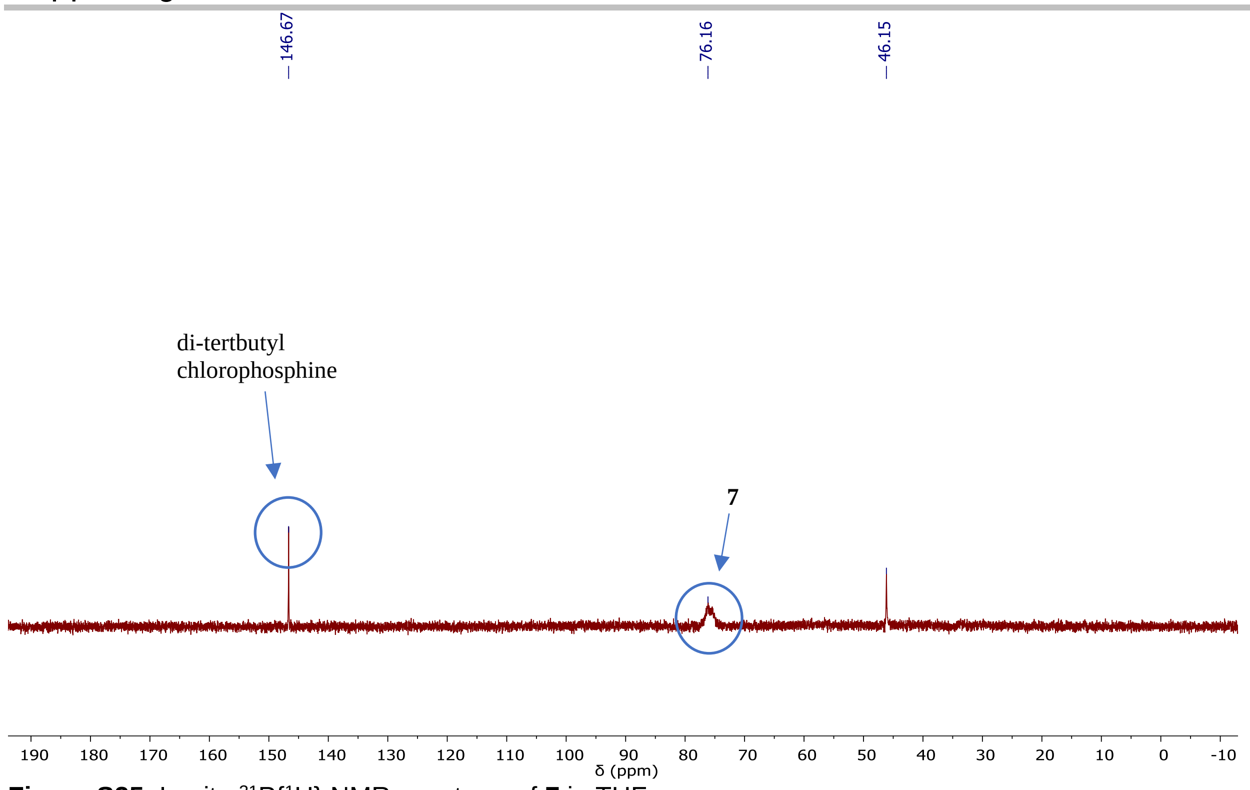

Figure S25. In-situ $^{31}P\{^{1}H\}$ NMR spectrum of **7** in THF.

S3. UV-Vis Spectrum

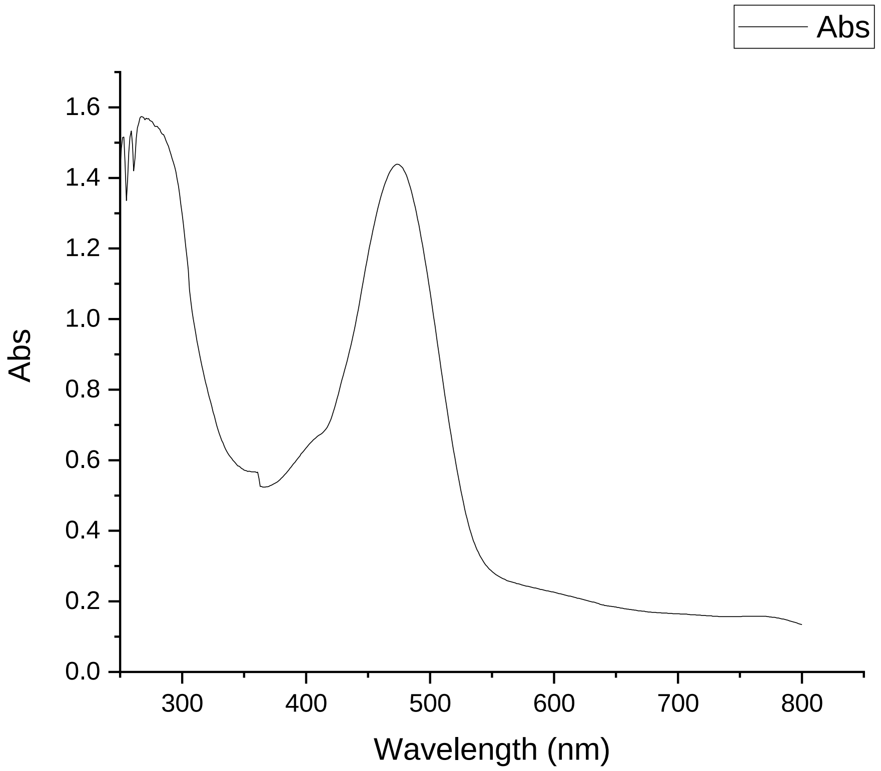

Figure S26. UV-vis spectrum of compound **5**

S3. X-ray Data Collection and Structural Refinement

The X-ray diffraction intensity data of all compounds were measured using a Bruker D8 Quest diffractometer equipped with a CCD detector at 100 K and employing Mo K α radiation (λ = 0.71073 Å) with the SMART suite of programs. SAINT was used to correct Lorentz and polarization effects and SADABS was used to correct absorption effects. The SHELXTL suite of programs were employed for solving of structures and structural refinement.[S3,S4] Direct methods were employed for the location of the heavier atoms, ensued by difference maps for the lighter, non-hydrogen atoms for structural solution. Anisotropic thermal parameters were used for the refinement of all non-hydrogen atoms. Deposition numbers 2266104 (for **2**), 2266105 (for **3**), 2266106 (for **4**), 2266107 (for **5**) and 2266108 (for **6a** and **6b**) and 2380825 (for **7**) contain the supplementary crystallographic data for this paper. These data are provided free of charge by the joint Cambridge Crystallographic Data Centre and Fachinformationszentrum Karlsruhe Access Structures service.

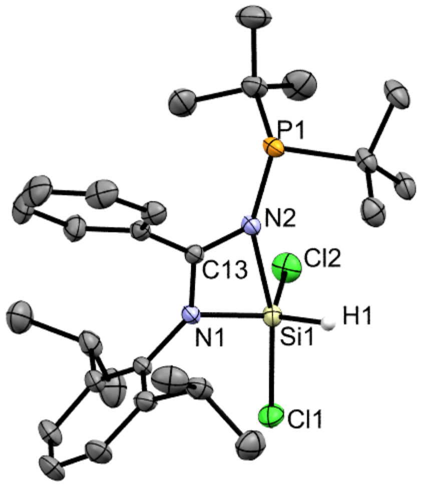

Figure S27. X-ray crystal structure of **2** with thermal ellipsoids at 50 % probability. All H atoms are omitted for clarity. Selected bond lengths (Å) and angles (deg) of **2**: Si1-Cl1 2.1197(6), Si1-Cl2 2.0590(6), Si1-N1 1.7663(12), Si1-N2 2.1139(13), N2-Si1-Cl1 164.74(4), N1-Si1-Cl2 117.66(5), N1-Si1-H1 119.5(7), H1-Si1-Cl2 117.7(7).

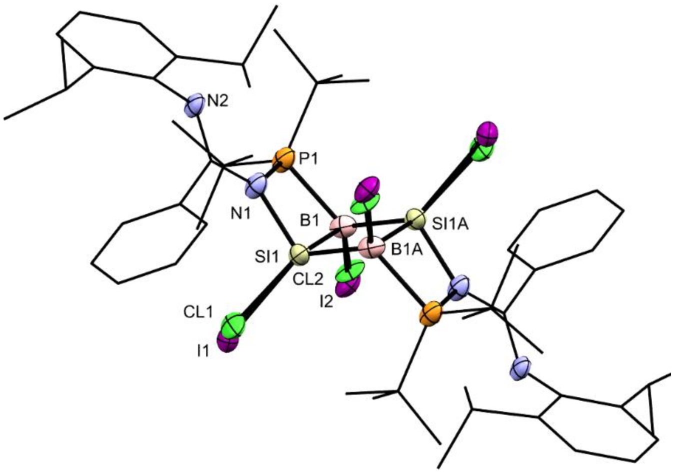

Figure S28. X-ray crystallography of co-crystal **6a** and **6b** with thermal ellipsoids at 50 % probability. All H atoms are omitted for clarity. The I:Cl occupancy ratio at the Si atoms is 0.49:0.52 and at the B atoms is 0.52:0.48. Selected bond lengths (Å): Si1-Cl1 2.127(7), Si1-I1 2.470(2), B1-Cl2 1.837(6), B1-I2 2.237(4).

Table S1. X-Ray crystallographic data for compound **2** - **4**

	**2**	**3**	**4**
Formula	$C_{27}H_{41}Cl_2N_2PSi$	$C_{27}H_{40}ClN_2PSi$	$C_{27}H_{40}BClI_3N_2PSi$
Fw	523.58	487.12	878.63
Temperature/K	150(2)	150(2)	100(2)
crystal system	monoclinic	orthorhombic	monoclinic
space group	*P* 1 21/*n* 1	*P n a* 21	*P* 1 21/*n* 1
a (Å)	8.9802(3)	15.8487(6)	9.1943(8)
b (Å)	15.5193(7)	10.8456(4)	20.6319(16)
c (Å)	21.2731(8)	16.0281(5)	17.5682(14)
α (deg)	90	90	90
β (deg)	100.9553(12)	90	98.264(3)
γ (deg)	90	90	90
V (Å^3)	2910.7(2)	2755.05(17)	3298.0(5)
Z	4	4	4
d_{calcd} (g cm^{-3})	1.195	1.174	1.770
μ (mm^{-1})	0.337	0.257	3.028
F (000)	1120	1048	1704
crystal size (mm)	0.280 x 0.300 x 0.320	0.120 x 0.180 x 0.200	0.12 x 0.14 x 0.22
2θ range (deg)	4.701 < 2θ < 63.55	4.534 < 2θ < 63.63	4.892 < 2θ < 63.03
index range	$-13 \le h \le 11$,<br>$-23 \le k \le 18$,<br>$-31 \le l \le 31$	$-24 \le h \le 24$,<br>$-14 \le k \le 16$,<br>$-22 \le l \le 25$	$-13 \le h \le 13$,<br>$-30 \le k \le 30$,<br>$-25 \le l \le 24$
no. of reflections collected	50504	34130	66149
no. of independent reflections	10158	10799	10942
$R1, wR2$ ($I > 2\sigma(I)$)	0.0457/0.1000	0.0606/0.120	0.0477/0.1016
$R1, wR2$ (all data)	0.0824/0.1149	0.1263/0.1451	0.0596/0.1068
goodness of fit, F^2	1.026	1.028	1.188
no. of data/restraints/parameters	10158 / 0 / 312	10799 / 1 / 300	10942 / 0 / 335
largest diff peak and hole, eÅ^{-3}	0.424 and -0.462	1.020 and -0.501	2.042 and -1.219

Table S2. X-Ray crystallographic data for compound **5-7**

	**5**	**6a** and **6b**	**7**
Formula	$C_{54}H_{80}B_2N_4P_2Si_2$	$C_{61}H_{88}B_2Cl_{2.05}I_{1.95}N_4P_2Si_2$	$C_{31}H_{48}BCl_3N_2OPSi$
Fw	924.96	1337.22	640.93
Temperature/K	100(2)	100(2)	100(2)
crystal system	monoclinic	triclinic	orthorhombic
space group	*P* 1 21/n 1	*P* -1	*P* 21 21 21
a (Å)	9.9510(4)	10.9260(9)	11.1528(2)
b (Å)	24.2287(10)	12.0567(10)	22.0855(5)
c (Å)	11.9140(5)	13.3072(10)	27.2759(7)
α (deg)	90	105.159(3)	90
β (deg)	114.1872(13)	106.206(3)	90
γ (deg)	90	95.699(3)	90
V (Å^3)	2620.29(19)	1596.4(2)	6718.5(3)
Z	2	1	8
d_{calcd} (g cm^{-3})	1.172	1.391	1.267
μ (mm^{-1})	0.168	1.175	3.463
F (000)	1000	688	2728
crystal size (mm)	0.010 x 0.100 x 0.140	0.060 x 0.160 x 0.210	0.005 x 0.010 x 0.120
2*θ* range (deg)	4.792° < 2*θ* < 54.72°	4.803° < 2*θ* < 58.58°	5.148° < 2*θ* < 136.7°
index range	-12 ≤ *h* ≤ 12,<br>-31 ≤ *k* ≤ 31,<br>-15 ≤ *l* ≤15	-15 ≤ *h* ≤ 15,<br>-16 ≤ *k* ≤ 16,<br>-18 ≤ *l* ≤15	-13 ≤ *h* ≤13,<br>-26 ≤ *k* ≤25,<br>-32 ≤ *l* ≤32
no. of reflections collected	35324	42485	57694
no. of independent reflections	6013	8947	12255
R1, wR2 (I > 2σ(I))	0.0616/0.1079	0.0591/0.1155	0.0402/0.0906
R1, wR2 (all data)	0.1254/0.1368	0.1122/0.1342	0.0499/ 0.0950
goodness of fit, *F*^{2}	1.020	1.019	1.029
no. of data/restraints/parameters	6013 / 0 / 276	8947 / 0 / 389	12255 / 230 / 787
largest diff peak and hole, eÅ^{-3}	0.537 and -0.384	1.053 and -0.502	0.540 and -0.278

S4. Theoretical studies

Geometry optimizations were carried out using density functional theory at the M06-2X level[S5] in conjunction with the def2-TZVP basis set.[S6] Stationary points were characterized as minima by calculating the Hessian matrix analytically. The calculations were carried out using the program package Gaussian 16, Revision C.01.[S7] The NBO analysis[S8] was done with the internal module of Gaussian 16 (NBO Version 5.0) at the M06-2X/def2-TZVP level of theory. The quantum theory of atoms in molecules (QTAIM) method[S9] was employed for the characterization of the Laplacian of electron density and electron localization function (ELF)[S10] using the Multiwfn 3.8 package.[S11] Nucleus Independent Chemical Shift (NICS)[S12] calculations with the Gauge-Independent Atomic Orbital (GIAO) method.[S13] Anisotropy of the Current Induced Density (ACID),[S14] and adaptive natural density partitioning (AdNDP) method[S15] were conducted at the M06-2X/def2-TZVP level of theory using the Gaussian 16 C.01 program.

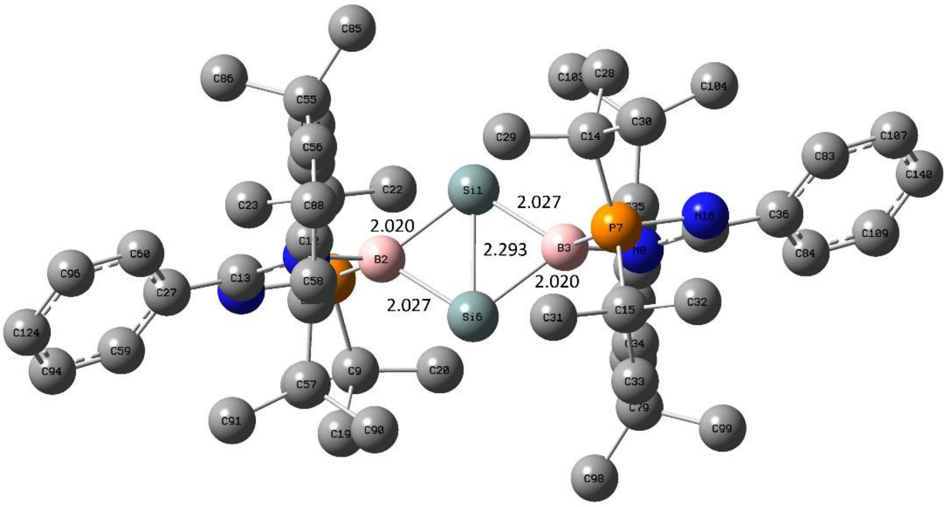

Figure S29. Optimised geometries of compound **5** at M06-2X/def2-TZVP level of theory (Grey: C, Blue: N, Orange: P, Pink: B, Green: Si). Hydrogen atoms are omitted for clarity. The bond lengths displayed are measured in Angstroms (Å).

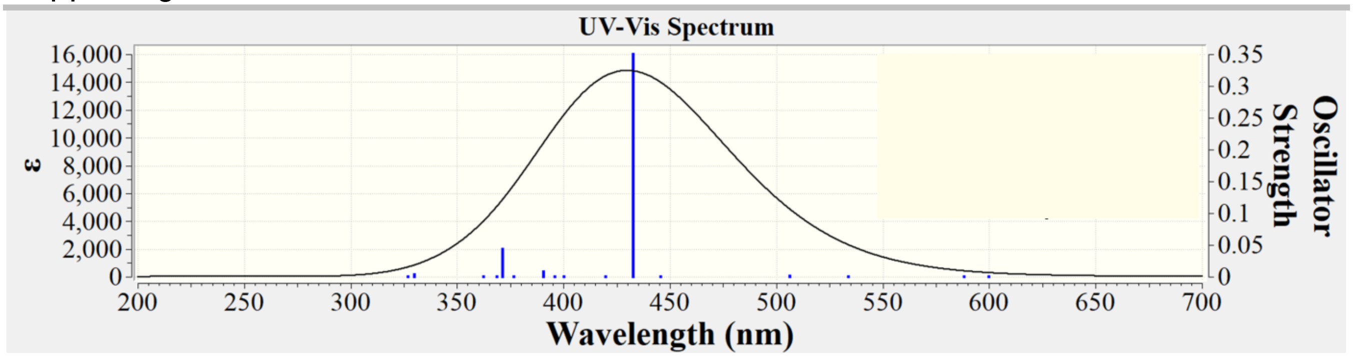

State	l (nm)	f_{calc}	nature			contribution
S_1	432.74	0.3500	HOMO-1	→	LUMO	68%
			HOMO-1	→	LUMO+3	12%
S_2	371.51	0.0428	HOMO-2	→	LUMO	51%
			HOMO	→	LUMO+1	17%
			HOMO	→	LUMO+2	42%

Figure S30. UV-Vis spectrum and absorption band of compound **5** (f_{calc} = oscillator strength).

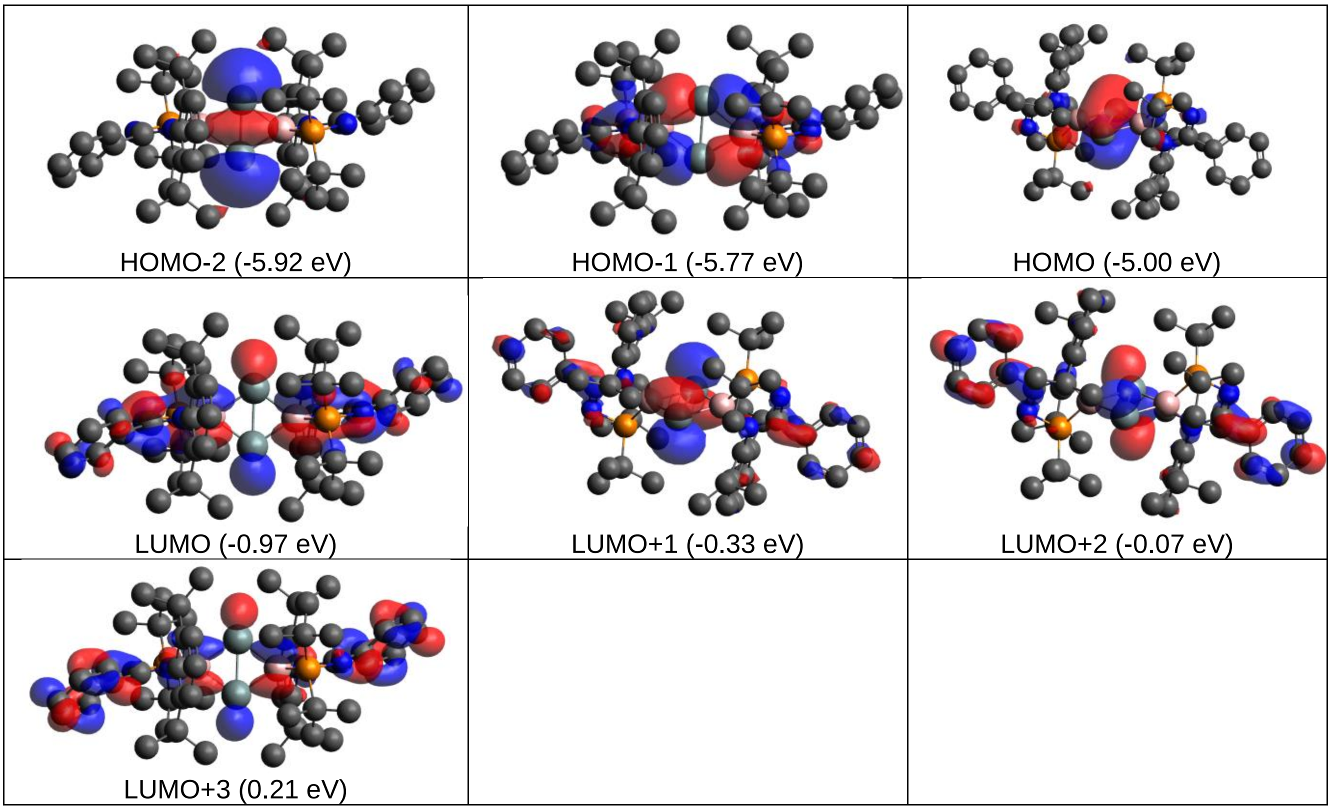

Figure S31. Molecular orbitals of compound **5**.

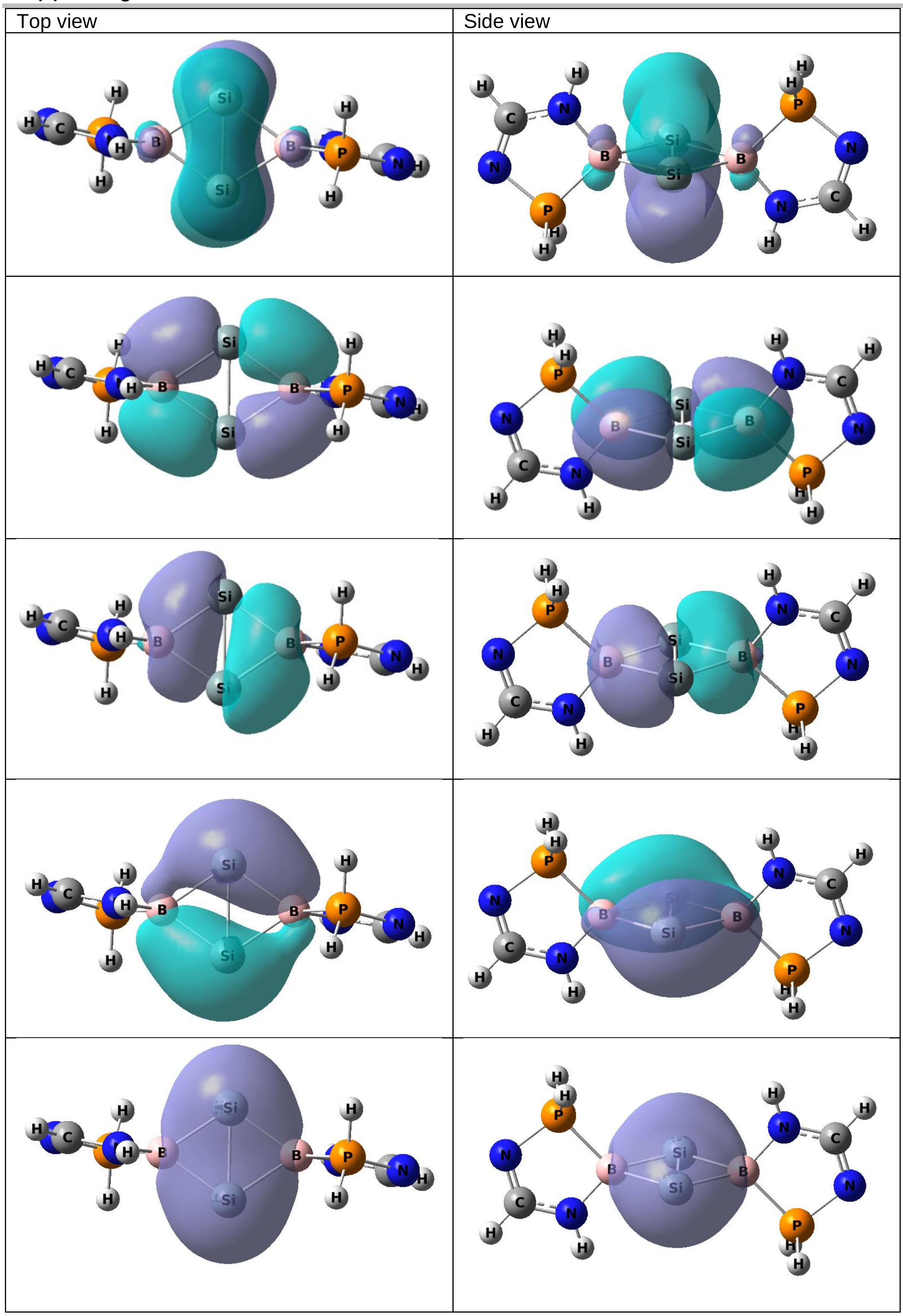

Figure S32. Adaptive natural population density (AdNDP) analysis of a simplified truncated model **5-H**, where substituents (Dipp, Ph, *t*Bu) in compound **5** are substituted by hydrogen atom for clarity.

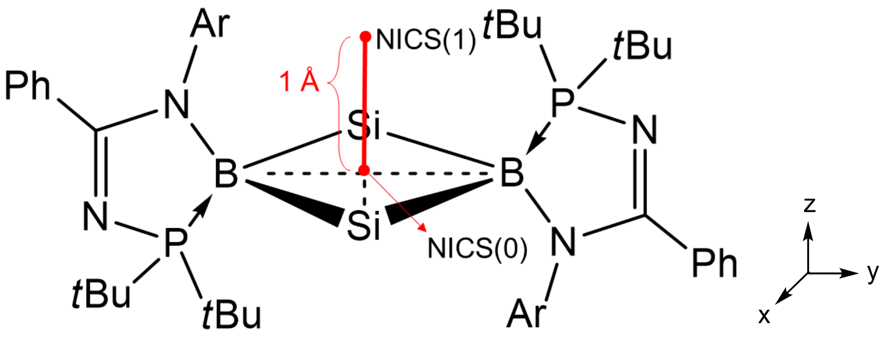

NICS(0)	-38.52 ppm
NICS(0)$_{zz}$*	-45.50 ppm
NICS(0)$_{xy}$	-0.20 ppm
NICS(1)	-22.12 ppm
NICS(1)$_{zz}$*	-34.01 ppm
NICS(1)$_{xy}$	-1.33 ppm

Figure S33. Calculated NICS value under the M06-2X/Def2-TZVP of theory. NICS(0) and NICS(1) represents the chemical shift at the Si_2B_2 ring center and the chemical shift at 1Å above the ring center. zz represents the chemical shift alone the Z-axis.

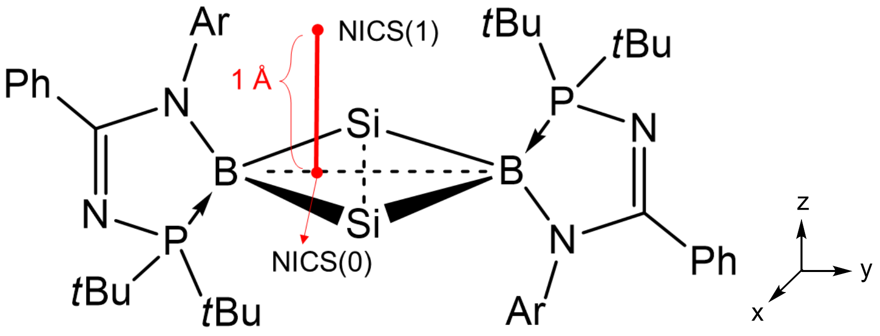

NICS(0)	-38.70 ppm
NICS(0)zz*	-40.59 ppm
NICS(0)xy	-1.44 ppm
NICS(1)	-13.77 ppm
NICS(1)zz*	-22.41 ppm
NICS(1)xy	-1.72 ppm

Figure S34. Calculated NICS value under the M06-2X/Def2-TZVP of theory. NICS(0) and NICS(1) represents the chemical shift at the left side of the Si_2B_2 ring and the chemical shift at 1Å above the left side of the ring. zz represents the chemical shift alone the Z-axis.

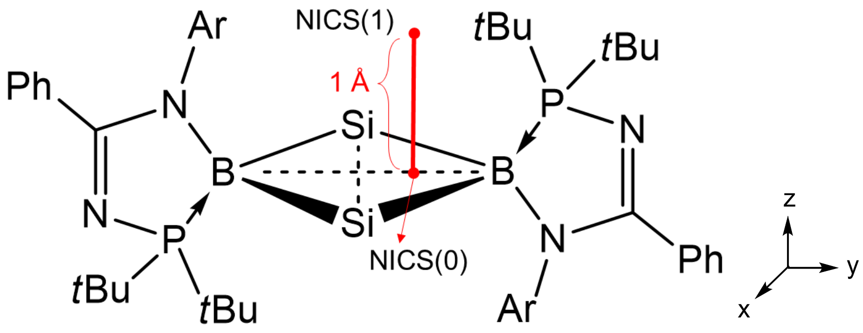

NICS(0)	-38.71 ppm
NICS(0)zz*	-40.56 ppm
NICS(0)xy	-1.45 ppm
NICS(1)	-13.75 ppm
NICS(1)zz*	-22.42 ppm
NICS(1)xy	-1.74 ppm

Figure S35. Calculated NICS value under the M06-2X/Def2-TZVP of theory. NICS(0) and NICS(1) represents the chemical shift at the right side of the Si_2B_2 ring and the chemical shift at 1Å above the right side of the ring. zz represents the chemical shift alone the Z-axis.

Overall

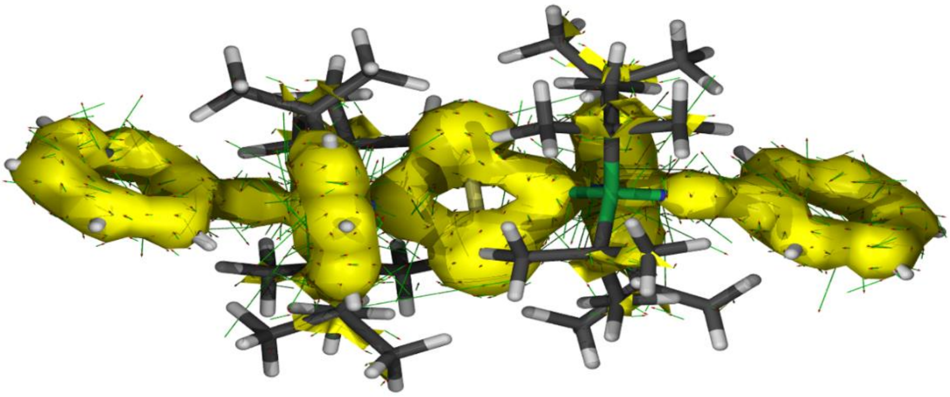

HOMO

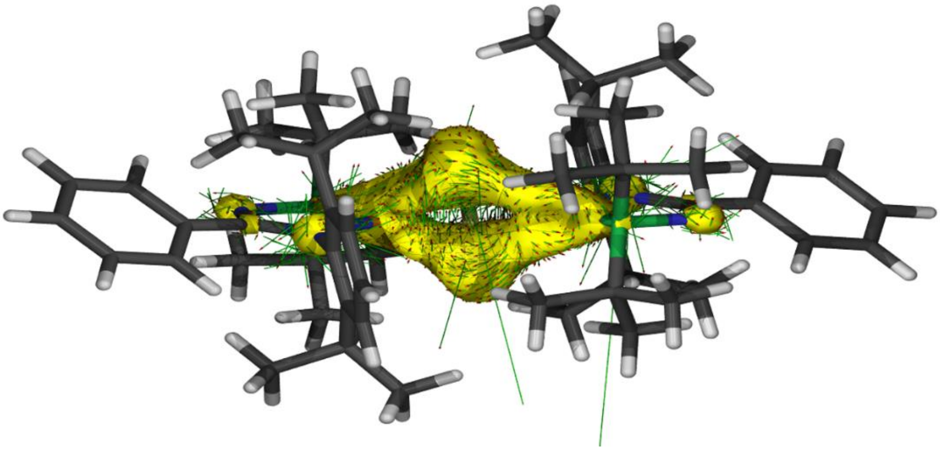

HOMO-1

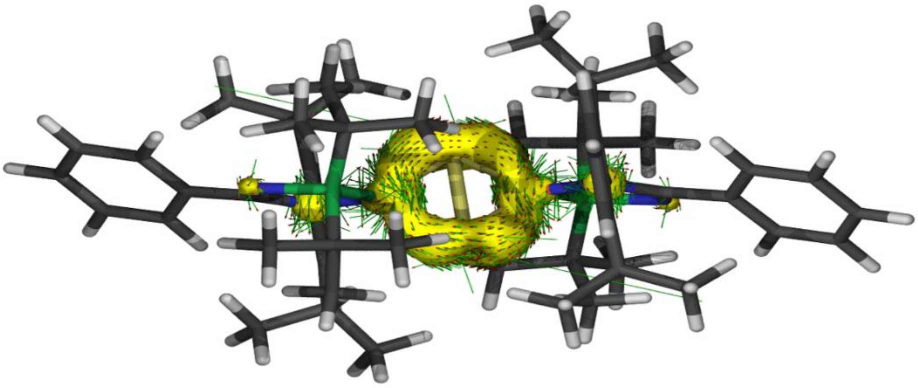

Figure S36. Anisotropy of the Current Induced Density (ACID) of compound **5** (Top view).

Overall

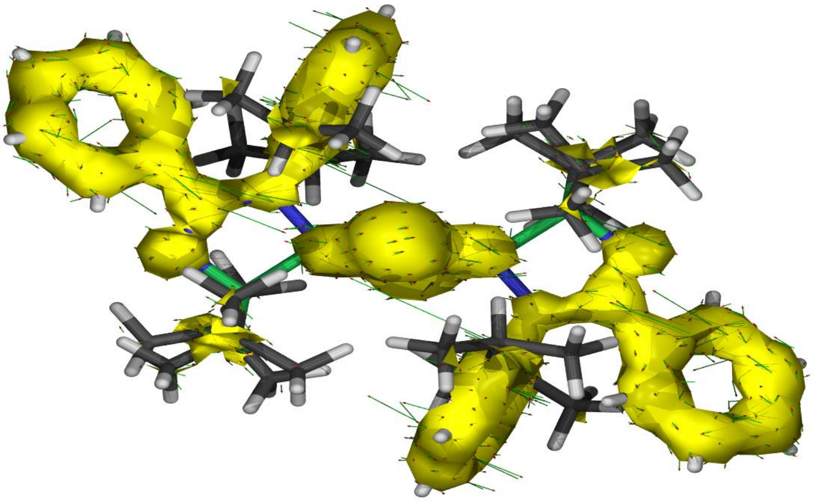

HOMO

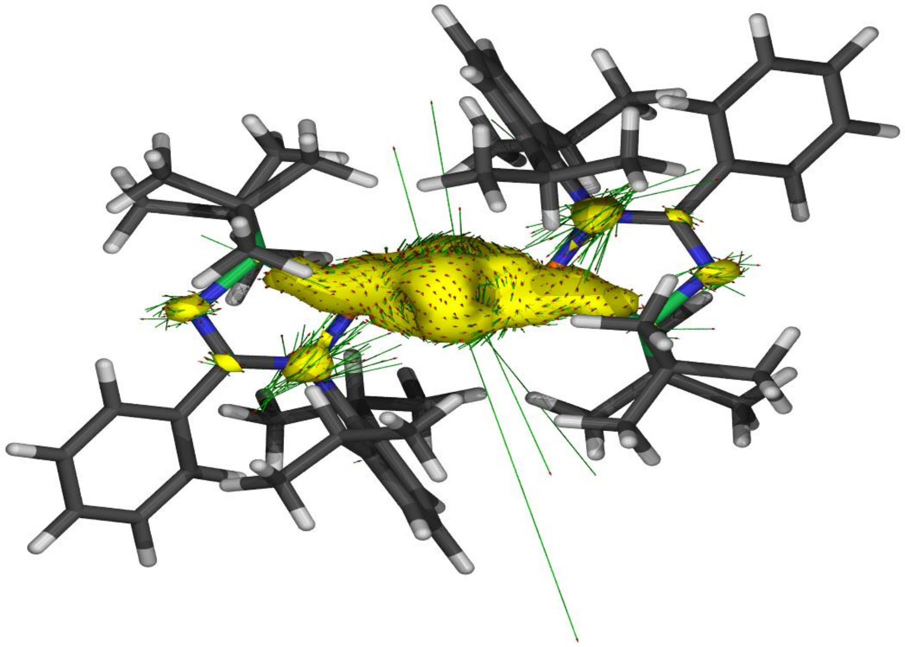

HOMO-1

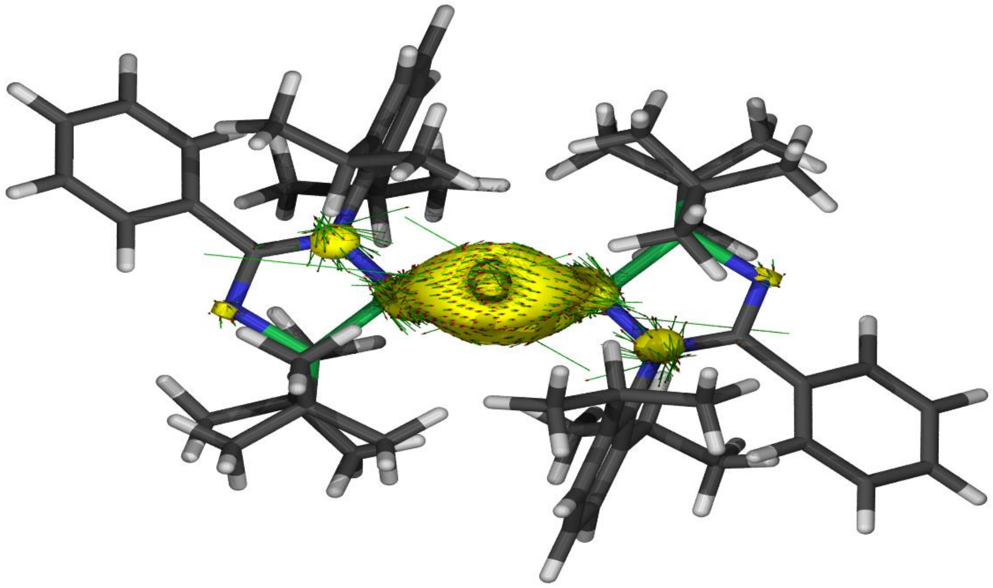

Figure S37. Anisotropy of the Current Induced Density (ACID) of compound **5** (side view).

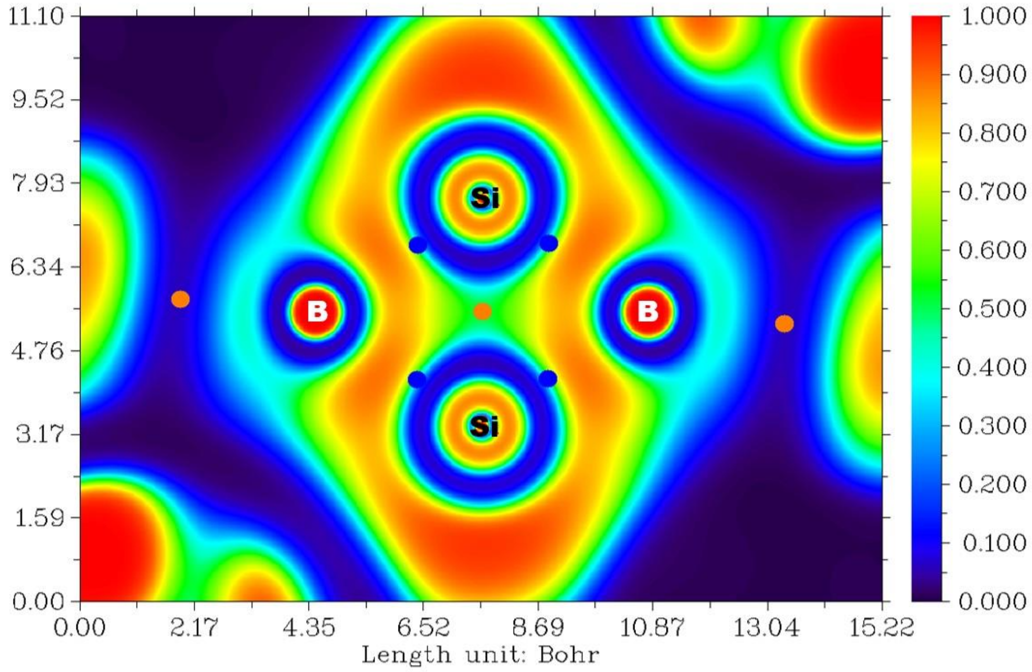

(a)

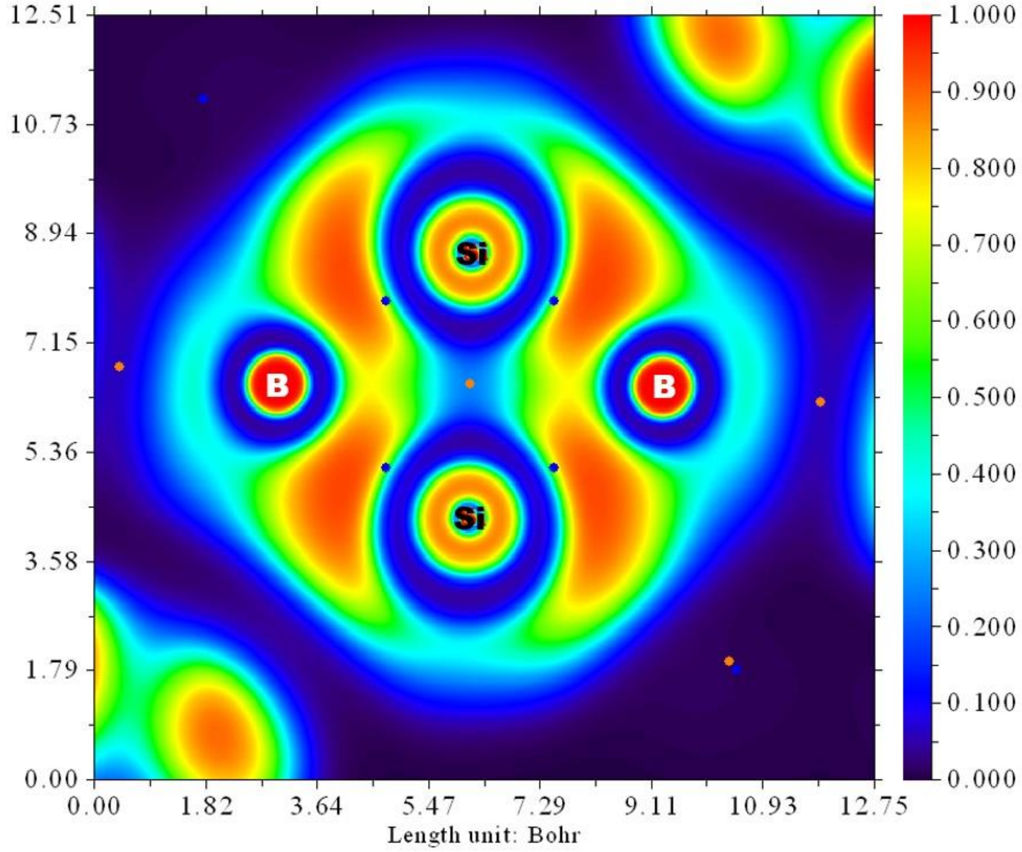

(b)

Figure S38. (a) Color-filled map of ELF of **5**; the color reflects the degree of electron localization of the core electrons; red and blue represent strong and weak localization. (b) Removing electrons from the σ-bonding HOMO−2 results the almost non-existent chemical bond between the two bridgehead Si atoms (ELF ≈ 0.01).

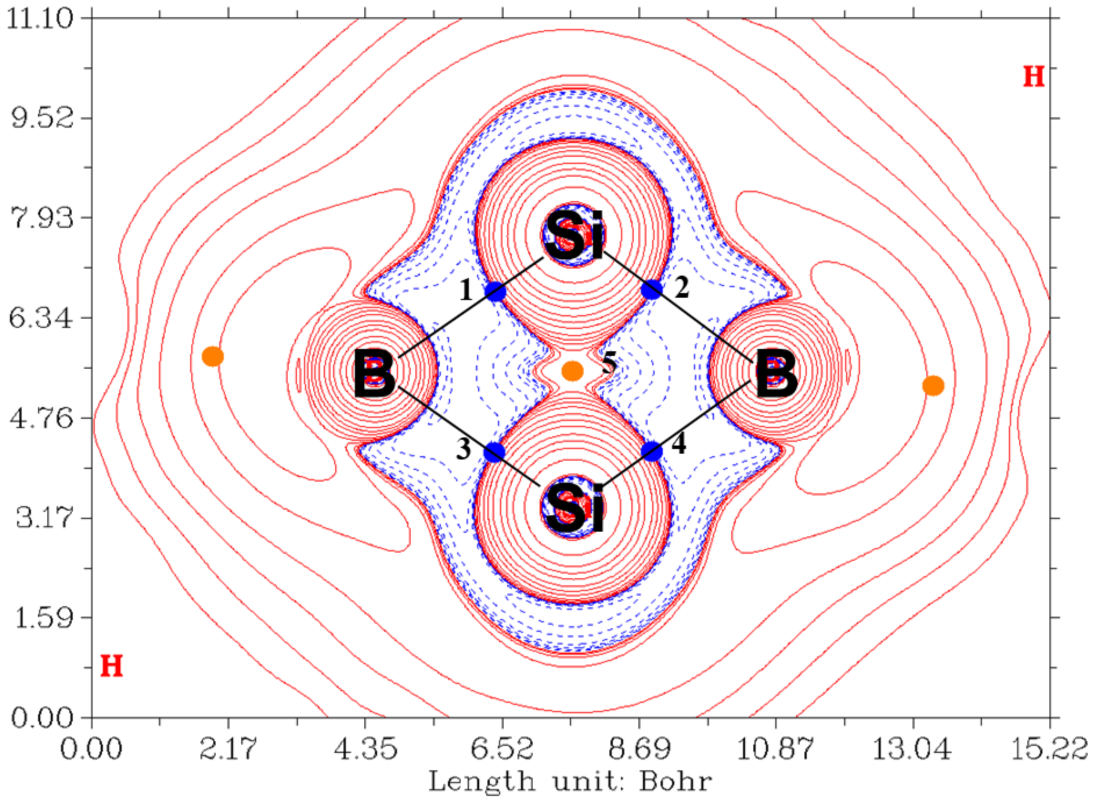

CP	CP type	Electron density $\rho(r_c)$ (e·a^{-3})	Laplacian electron density $\nabla^2\rho(r_c)$ (e·a^{-5})	Total energy electron density $H(r_c)$ (Hartree·a^{-3})
1	3, –1	0.095	0.013	–0.060
2	3, –1	0.094	0.004	–0.060
3	3, –1	0.094	0.004	–0.060
4	3, –1	0.095	0.013	–0.060
5	3, +1	0.071	0.008	–0.032

(a)

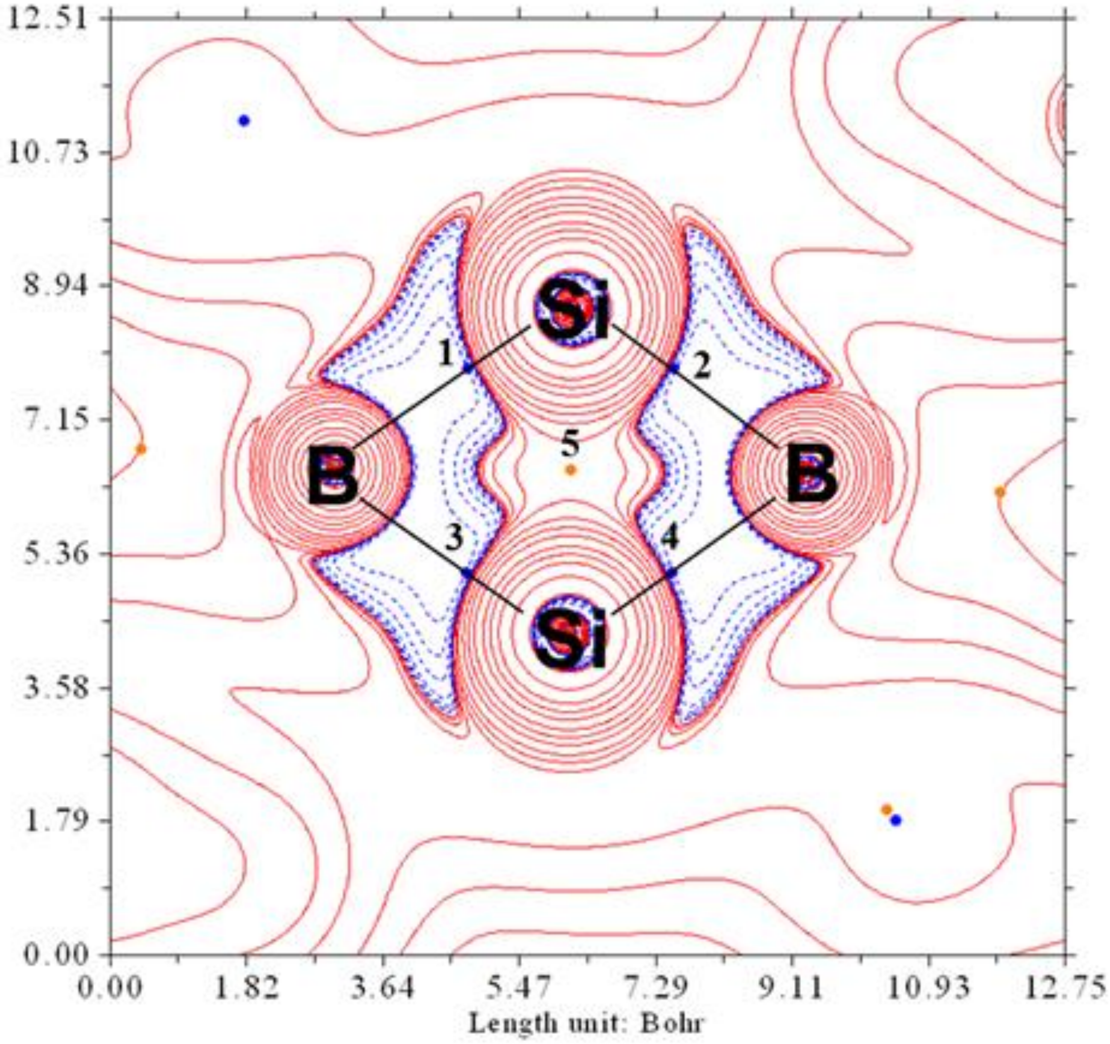

CP	CP type	Electron density $\rho(r_c)$ (e·a^{-3})	Laplacian electron density $\nabla^2\rho(r_c)$ (e·a^{-5})	Total energy electron density $H(r_c)$ (Hartree·a^{-3})
1	3, –1	0.091	–0.080	–0.057
2	3, –1	0.092	–0.064	–0.059
3	3, –1	0.092	–0.064	–0.059
4	3, –1	0.091	–0.080	–0.057
5	3, +1	0.049	0.063	–0.017

(b)

Figure S39. (a) Laplacian distribution of electron energy of the Si_2B_2 ring plane in **5**. Positive and negative area are represented by crimson and blue lines, representing electron depletion and accumulation, respectively. (b) By removing electrons from the σ-bonding HOMO−2, the electron density concentrations within the Si_2B_2 ring plane in **5** are altered.

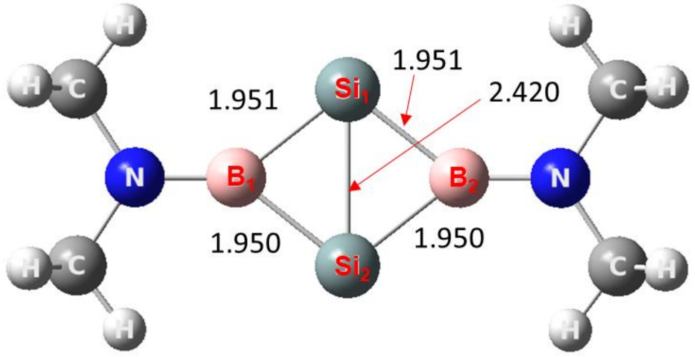

Figure S40. Optimised geometries of the model molecule **5-NMe$_2$** at M06-2X/def2-TZVP level of theory (Grey: C, Blue: N, Pink: B, Green: Si). Hydrogen atoms are omitted for clarity. The bond lengths displayed are measured in Angstroms (Å).

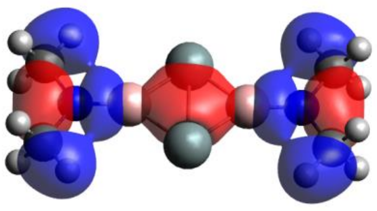 HOMO-17 (-15.50 eV)	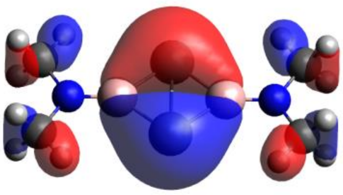 HOMO-6 (-11.72 eV)	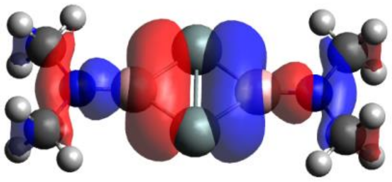 HOMO-5 (-10.83 eV)
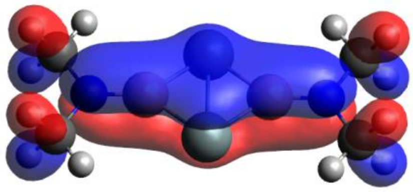 HOMO-4 (-9.320 eV)	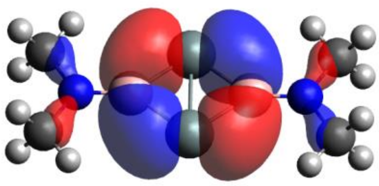 HOMO-2 (-7.094 eV)	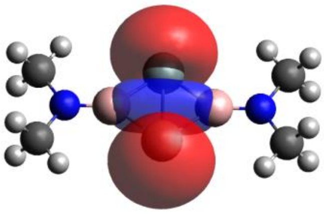 HOMO-1 (-6.937 eV)
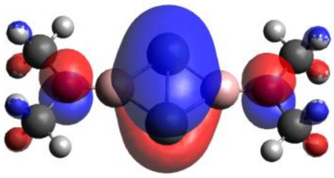 HOMO (-6.313 eV)	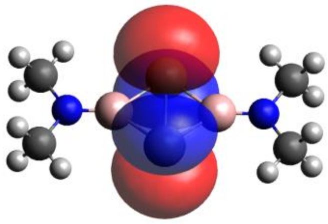 LUMO (-0.681 eV)	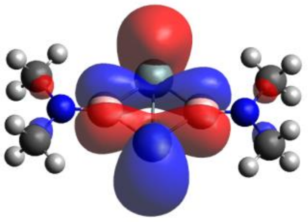 LUMO+1 (-0.655 eV)
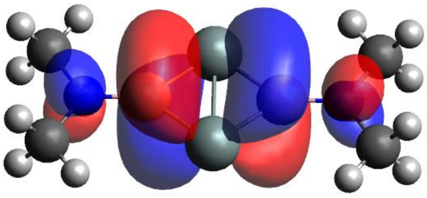 LUMO+2 (0.317 eV)		

Figure S41. Molecular orbitals of the model molecule **5-NMe$_2$**.

	**5**	**5-NMe₂**
$EDDB_{4MR}$	1.901	3.467
$EDDB_{4MR\text{-}\pi}$	0.708	1.396
Ratio $\left(= \frac{EDDB_{4MR-\pi}}{EDDB_{4MR}} \times 100\%\right)$	37.2%	40.2%

Table S3. Electron Density of Delocalized Bonds (EDDB) value of compound **5** and **5-NMe$_2$** at M06-2X/ def2-TZVP level of theory.

	$EDDB_{4MR}$	$EDDB_{4MR\text{-}\pi}$
**5**	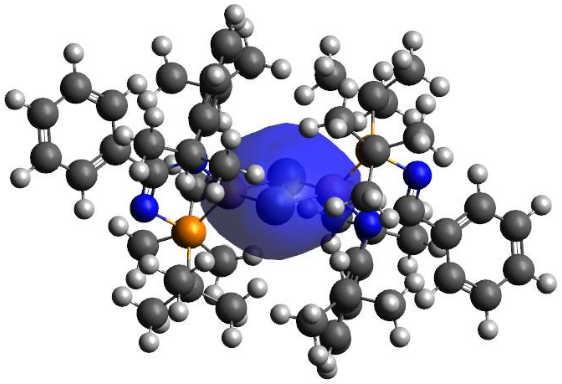	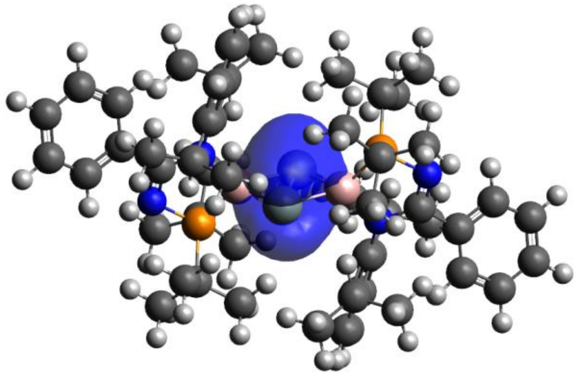
**5-NMe$_2$**	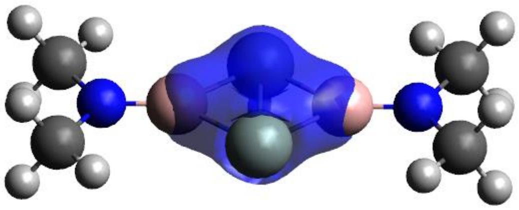	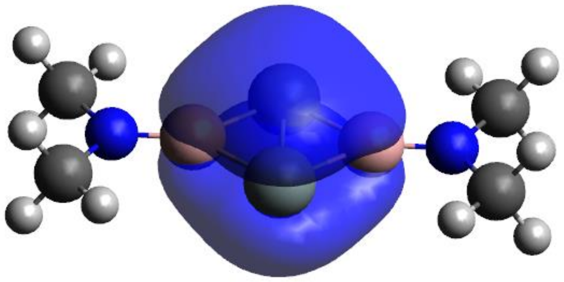

Figure S42. Visualized EDDB of compound **5** and **5-NMe$_2$** at M06-2X/ def2-TZVP level of theory.

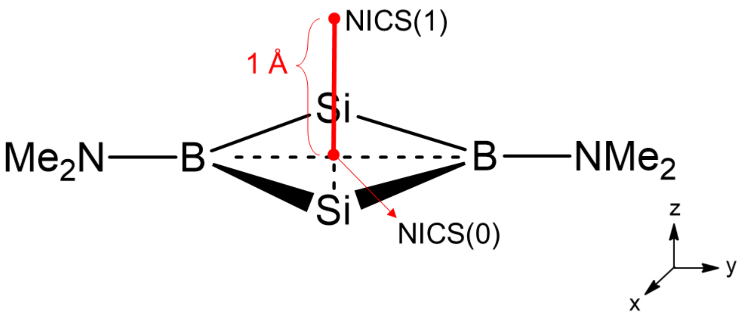

NICS(0)	-37.45 ppm
NICS(0)zz*	-49.88 ppm
NICS(1)	-23.22 ppm
NICS(1)zz*	-35.83 ppm

Figure S43. Calculated NICS value under the M06-2X/Def2-TZVP of theory. NICS(0) and NICS(1) represents the chemical shift at the Si-B-Si-B ring center and the chemical shift 1Å above the ring center. zz represents the chemical shift alone the Z-axis.

Table S4. Cartesian coordinates for **5.**
M06-2X/def2-TZVP

Atomic Number	Coordinates (Angstroms) X	Y	Z
Si	-0.16260400	0.11649900	-1.12867300
B	1.51933200	0.66833200	-0.15577900
B	-1.51928400	-0.66833200	0.15626600
P	2.19627400	2.52709100	-0.14733600
N	2.92745600	0.00623200	-0.25322800
Si	0.16272900	-0.11666600	1.12915900
P	-2.19626200	-2.52708300	0.14794100
N	-2.92742100	-0.00617900	0.25340300
C	1.80824200	3.53786100	1.37829200
C	2.02747800	3.51070200	-1.73712000
N	3.83395700	2.13943500	-0.05368900
C	3.04861600	-1.41554600	-0.34428000
C	3.99905500	0.83921900	-0.11409300
C	-1.80784700	-3.53816600	-1.37737500
C	-2.02790500	-3.51045500	1.73792400
N	-3.83390600	-2.13938600	0.05381600
C	-3.04856200	1.41563200	0.34407600
C	-3.99899300	-0.83916200	0.11402200
C	2.31332700	2.71165700	2.57122000
C	0.29430600	3.74101500	1.49922500
C	2.53883200	4.88354800	1.36855600
C	0.65175300	4.17884500	-1.82419600
C	3.15913600	4.53899000	-1.86579400
C	2.18473400	2.49744600	-2.88055600
C	2.93214800	-2.00807000	-1.62219100
C	3.26419600	-2.19303000	0.81072000
C	5.40385800	0.33882100	0.07160200
C	-2.31247200	-2.71215900	-2.57062900
C	-0.29388000	-3.74145800	-1.49775700
C	-2.53855400	-4.88378800	-1.36762700
C	-0.65240900	-4.17901500	1.82531400
C	-3.15989500	-4.53837600	1.86662100
C	-2.18498000	-2.49696500	2.88117600
C	-2.93234600	2.00844200	1.62188800
C	-3.26381800	2.19286900	-0.81115600
C	-5.40373900	-0.33877200	-0.07214700
H	1.80465500	1.73865000	2.63480300
H	2.10324500	3.27046600	3.49688500
H	3.39762900	2.53956100	2.50527200
H	-0.10460700	4.42474200	0.73909900
H	0.06880300	4.17577400	2.48708400
H	-0.25257400	2.79014500	1.41319900
H	2.43997200	5.34368600	2.36459400
H	2.10143900	5.58073700	0.64107100
H	3.61019300	4.76034300	1.15121800
H	-0.16421200	3.48136700	-1.57376100
H	0.58120000	5.05149200	-1.15925600
H	0.48891300	4.53191100	-2.85522000
H	4.13798900	4.05841600	-1.73093400
H	3.07226100	5.36109000	-1.14693600
H	3.11838300	4.97512900	-2.87656100
H	1.34236700	1.79326000	-2.91835800
H	3.12137300	1.92698000	-2.78038000
H	2.22306400	3.04675900	-3.83424800
C	2.74019900	-1.15397400	-2.86846600
C	3.06449100	-3.39420800	-1.72528400
C	3.43649800	-1.59435600	2.20108800
C	3.37977100	-3.58059800	0.65483300
C	6.22036600	1.11450600	0.90928800
C	5.94811300	-0.81599500	-0.50989800
H	-2.10223600	-3.27121800	-3.49610400
H	-1.80364400	-1.73923700	-2.63430900
H	-3.39676300	-2.53986600	-2.50501200
H	0.10460900	-4.42553500	-0.73772400
H	-0.06799700	-4.17584600	-2.48568900
H	0.25309100	-2.79068900	-1.41115200
H	-2.43910900	-5.34426600	-2.36342900
H	-3.61002600	-4.76041700	-1.15099500
H	-2.10169900	-5.58076600	-0.63964400

H	0.16381300	-3.48181700	1.57497100
H	-0.58199100	-5.05176300	1.16049500
H	-0.48988700	-4.53201100	2.85640400
H	-4.13859200	-4.05748700	1.73176900
H	-3.07331600	-5.36050500	1.14775100
H	-3.11926000	-4.97452200	2.87738800
H	-1.34240100	-1.79303200	2.91897300
H	-3.12141500	-1.92622000	2.78077900
H	-2.22362100	-3.04610600	3.83495200
C	-2.74086600	1.15454900	2.86838500
C	-3.06447000	3.39463100	1.72461200
C	-3.43587700	1.59393300	-2.20144700
C	-3.37927600	3.58049400	-0.65561600
C	-6.22002800	-1.11460700	-0.90990800
C	-5.94813900	0.81616600	0.50898300
C	1.89261100	-1.83250900	-3.94428800
C	4.07973600	-0.69207600	-3.45597100
H	2.19747100	-0.25502100	-2.55406500
C	3.28667900	-4.17909800	-0.59575200
H	2.98740400	-3.87106400	-2.70373000
C	2.43543400	-2.17567800	3.20593700
C	4.86140400	-1.81039800	2.73239400
H	3.24808500	-0.51174400	2.12825600
H	3.55810300	-4.20125600	1.53584900
C	7.52758500	0.73085700	1.19092000
H	5.80136600	2.02436000	1.33761000
C	7.26599000	-1.18510900	-0.24348700
H	5.35886300	-1.44361400	-1.17269100
C	-1.89522800	1.83389600	3.94517700
C	-4.08054800	0.69139400	3.45453300
H	-2.19692900	0.25611900	2.55456900
C	-3.28632000	4.17928100	0.59483600
H	-2.98747400	3.87173200	2.70294100
C	-2.43466600	2.17508500	-3.20625600
C	-4.86068300	1.80989400	-2.73306300
H	-3.24746900	0.51133300	-2.12838400
H	-3.55735500	4.20096800	-1.53681500
C	-7.52716100	-0.73098900	-1.19198000
H	-5.80092200	-2.02455100	-1.33793700
C	-7.26593300	1.18525400	0.24213000
H	-5.35906400	1.44390400	1.17181700
H	0.93599800	-2.18675600	-3.53426200
H	2.41323600	-2.68615000	-4.40560900
H	1.67075800	-1.11393100	-4.74691100
H	4.71875500	-1.55375600	-3.70862500
H	4.63598500	-0.04862000	-2.75884300
H	3.90843300	-0.11463600	-4.37730600
H	3.38746600	-5.26143700	-0.69324100
H	2.59354600	-3.25703600	3.34309800
H	1.39899900	-2.01204900	2.88204900
H	2.56538800	-1.69434900	4.18681600
H	5.62837000	-1.40740200	2.05928700
H	5.06032400	-2.88519800	2.86818800
H	4.97442200	-1.32486100	3.71330200
C	8.05617700	-0.42419200	0.61509200
H	8.13744400	1.33899400	1.86052400
H	7.67107400	-2.08452700	-0.70896100
H	-0.93875000	2.18958900	3.53623000
H	-2.41749400	2.68664800	4.40617900
H	-1.67320300	1.11542900	4.74785200
H	-4.72054800	1.55248900	3.70662900
H	-4.63557700	0.04752000	2.75685400
H	-3.90962200	0.11405300	4.37600200
H	-3.38695700	5.26166300	0.69204300
H	-2.59287800	3.25639000	-3.34374800
H	-1.39826800	2.01166700	-2.88213900
H	-2.56438700	1.69347600	-4.18703200
H	-5.62779300	1.40699700	-2.06006700
H	-5.05957100	2.88467700	-2.86904700
H	-4.97349800	1.32422300	-3.71392900
C	-8.05589300	0.42418400	-0.61652400
H	-8.13684200	-1.33924600	-1.86163700
H	-7.67113100	2.08477300	0.70731400
H	9.08180100	-0.72743500	0.83132300
H	-9.08144900	0.72740600	-0.83310300

Table S5. Cartesian coordinates for **5-NMe$_2$.**
M06-2X/def2-TZVP

Atomic Number	Coordinates (Angstroms) X	Y	Z
B	-1.64225300	1.81237200	0.29169000
B	1.35645200	1.98376400	-0.29171500
Si	-0.21185500	3.10712000	0.00016400
Si	-0.07402900	0.69052700	-0.00017900
N	2.72057400	2.06222800	-0.55687700
N	-3.00642600	1.73473900	0.55678300
C	-3.85299000	2.90203000	0.70797800
C	-3.71499300	0.47881100	0.70764500
C	3.42848200	3.31844400	-0.70822900
C	3.56779800	0.89530500	-0.70739700
H	-3.26007600	3.81506600	0.58388400
H	-4.32267300	2.91719000	1.70634500
H	-4.66062500	2.89875800	-0.04389500
H	-3.02220800	-0.36100700	0.58377500
H	-4.51752000	0.39029100	-0.04448200
H	-4.18028100	0.41039600	1.70584200
H	2.73560400	4.15796500	-0.58287500
H	3.89227000	3.38750600	-1.70708200
H	4.23208800	3.40681600	0.04275300
H	2.97510000	-0.01799800	-0.58425700
H	4.37460600	0.89862300	0.04537400
H	4.03858400	0.88062100	-1.70524700

References

[S1] A. Velavan, S. Sumathi and K. K. Balasubramanian, *Eur. J. Org. Chem.*, 2014, **2014**, 5806-5815.

[S2] S. Warratz, L. Postigo and B. Royo, *Organometallics*, 2013, **32**, 893-897.

[S3] Bruker AXS Inc. SADABS. Madison, Wisconsin, USA, 2001.

[S4] G. Sheldrick, *Acta Crystallogr. C Struct. Chem.*, 2015, **71**, 3-8.

[S5] Y. Zhao, D. G. Truhlar, *J. Chem. Theory Comput.*, 2008, **4**, 1849-1868.

[S6] F. Weigend, *Phys. Chem. Chem. Phys.,* 2006, **8**, 1057-1065.

[S7] Gaussian 16, Revision C.01, M. J. Frisch, G. W. Trucks, H. B. Schlegel, G. E. Scuseria, M. A. Robb, J. R. Cheeseman, G. Scalmani, V. Barone, G. A. Petersson, H. Nakatsuji, X. Li, M. Caricato, A. V. Marenich, J. Bloino, B. G. Janesko, R. Gomperts, B. Mennucci, H. P. Hratchian, J. V. Ortiz, A. F. Izmaylov, J. L. Sonnenberg, D. Williams-Young, F. Ding, F. Lipparini, F. Egidi, J. Goings, B. Peng, A. Petrone, T. Henderson, D. Ranasinghe, V. G. Zakrzewski, J. Gao, N. Rega, G. Zheng, W. Liang, M. Hada, M. Ehara, K. Toyota, R. Fukuda, J. Hasegawa, M. Ishida, T. Nakajima, Y. Honda, O. Kitao, H. Nakai, T. Vreven, K. Throssell, J. A. Montgomery, Jr., J. E. Peralta, F. Ogliaro, M. J. Bearpark, J. J. Heyd, E. N. Brothers, K. N. Kudin, V. N. Staroverov, T. A. Keith, R. Kobayashi, J. Normand, K. Raghavachari, A. P. Rendell, J. C. Burant, S. S. Iyengar, J. Tomasi, M. Cossi, J. M. Millam, M. Klene, C. Adamo, R. Cammi, J. W. Ochterski, R. L. Martin, K. Morokuma, O. Farkas, J. B. Foresman, and D. J. Fox, Gaussian, Inc., Wallingford CT, 2016.

[S8] E. D. Glendening, J. K. Badenhoop, A. E. Reed, J. E. Carpenter, J. A. Bohmann, C. M. Morales, F. Weinhold, NBO 5.0.; Theoretical Chemistry Institute, University of Wisconsin: Madison, 2001.

[S9] F. W. Bader, Atoms in Molecules: A Quantum Theory; Oxford University Press: New York, 1994.

[S10] A. D. Becke, K. E. Edgecombe, *J. Chem. Phys.*, 1990, **92**, 5397-5403.

[S11] T. Lu, F. Chen, *J. Comput. Chem.*, 2012, **33**, 580-592.

[S12] Z. Chen, C. S. Wannere, C. Corminboeuf, R. Puchta, P. v. R. Schleyer, *Chem. Rev.*, 2005, **105**, 3842-3888.

[S13] K. Wolinski, J. F. Hinton, P. Pulay, *J. Am. Chem. Soc.*, 1990, **112**, 8251-8260.

[S14] R. Herges, D. Geuenich, *J. Phys. Chem. A.*, 2001, **105**, 3214-3220.

[S15] D. Y. Zubarev, A. I. Boldyrev, *Phys. Chem. Chem. Phys.*, 2008, **10**, 5207-5217.
